# Sport-Specific Injury Mechanisms and Situational Patterns of ACL Injuries: A Comprehensive Systematic Review

**DOI:** 10.1007/s40279-025-02271-w

**Published:** 2025-07-21

**Authors:** Axel Sundberg, Johan Högberg, Filippo Tosarelli, Matthew Buckthorpe, Francesco Della Villa, Martin Hägglund, Kristian Samuelsson, Eric Hamrin Senorski

**Affiliations:** 1 Capio Ortho Center, Gothenburg, Sweden; 2 Unit of Physiotherapy, Department of Health and Rehabilitation, Institute of Neuroscience and Physiology, Sahlgrenska Academy, University of Gothenburg, Box 455, 405 30 Gothenburg, Sweden; 3 Sahlgrenska Sports Medicine Center, Sahlgrenska Academy, Gothenburg, Sweden; 4 Sportrehab Sports Medicine Clinic, Gothenburg, Sweden; 5 Education and Research Department, Isokinetic Medical Group, FIFA Medical Centre of Excellence, Bologna, Italy; 6 Faculty of Sport, Health and Technology, St Mary’s University, Twickenham, London, UK; 7 Unit of Physiotherapy, Department of Health, Medicine and Caring Sciences, Linköping University, Linköping, Sweden; 8 Swedish Olympic Committee, Stockholm, Sweden; 9 Football Research Group, Linköping University, Linköping, Sweden; 10 Department of Orthopaedics, Institute of Clinical Sciences, Sahlgrenska Academy, University of Gothenburg, Gothenburg, Sweden; 11 Department of Orthopaedics, Sahlgrenska University Hospital, Mölndal, Sweden

## Abstract

**Background:**

Anterior cruciate ligament (ACL) injury mechanisms are linked to distinct characteristics and situational patterns inherent to each sport. Understanding ACL mechanisms and situational patterns is crucial to tailor prevention strategies and end-stage rehabilitation practices, ultimately aiming to reduce the incidence of ACL injuries in sports.

**Objectives:**

We aimed to compile and synthesize data regarding the injury mechanism and sport-specific situational patterns leading to ACL injuries across various sports.

**Methods:**

We conducted a systematic search using MEDLINE, Embase, Cochrane Library, Allied and Complementary Medicine Database, and Physiotherapy Evidence Database in December 2022 and repeated in October 2023 to identify additional published articles. English-language articles investigating ACL injury mechanism, injury situations, and sport-specific situational patterns were included, without restrictions on publication dates. Data extraction was performed independently by two authors. Article quality assessment was conducted with the Quality Appraisal for Sports Injury Video Analysis Studies checklist for video analysis studies and with the JBI Critical Appraisal Checklist for Case Series for athlete- and medical staff-reported studies. The data on ACL injury mechanism, situational patterns, and biomechanics were synthesized into qualitative tables by sport. Biomechanical data of ankle, knee, and hip angles in the sagittal plane for football and basketball were quantitatively synthesized and illustrated using box plots.

**Results:**

This systematic review included 62 articles covering 20 sports and 5612 ACL injury situations. The distribution of non-contact, indirect contact, and direct contact ACL injuries varied by sport, reflecting each sport’s unique playing patterns and characteristics. Four main ACL injury categories were identified: (1) change of direction; (2) landing after a jump; (3) direct contact to the knee; and (4) gear-induced mechanisms. In team sports, change of direction injuries ranged from 26 to 70%. Landing injuries were most prevalent in sports involving overhead play, such as volleyball and badminton, accounting for 57–82% of ACL injuries. Direct contact to the knee was the leading cause of ACL injuries in combat sports (53–83%) but also significant in aggressive contact sports such as American Football and rugby. Gear-induced ACL injuries in alpine skiing and board sports occur because of the extended lever arm attached to the feet, and present specific injury mechanisms such as ‘valgus-external rotation,’ ‘slip and catch,’ and ‘tail landing.’

**Conclusions:**

The nature of ACL injuries varies significantly between sports, influenced by injury mechanism and sport-specific situational patterns. We propose a categorization system for ACL injury situations—change of direction, landing, direct contact, and gear-induced situation—based on the findings of this systematic review. This framework aims to facilitate the development of prevention and rehabilitation strategies transferable across various sports and their sport-specific patterns.

**Clinical Trial Registration:**

Registration number: PROSPERO CRD42022355173.

**Supplementary Information:**

The online version contains supplementary material available at 10.1007/s40279-025-02271-w.

## Key Points

**Table:** 

Anterior cruciate ligament injury mechanisms and situational patterns vary significantly across sports, reflecting each sport’s unique playing characteristics and demands.
Four main anterior cruciate ligament injury situations were identified across sports: change of direction, landing, direct contact towards the knee, and gear-induced injuries.
Understanding sport-specific anterior cruciate ligament injury patterns is essential for developing targeted injury prevention and rehabilitation strategies that align with the demands of each sport.

## Introduction

Rupture of the anterior cruciate ligament (ACL) represents a devastating knee injury associated with an expected rehabilitation period of 6–12 months after ACL reconstruction [1]. While the majority of professional athletes return to sport after an ACL injury [2], the risk of a second ACL injury remains high, with approximately one in five athletes encountering a second ACL injury [3]. In addition to the increased ACL injury risk, athletes face challenges including, but not limited to, reduced career longevity and a lower competitive level [4–7]. To reduce the occurrence of ACL injuries in sports, understanding the mechanism behind ACL injury situations stands as a crucial step [8]. Differences in ACL injury mechanisms are linked to distinct characteristics and situational patterns inherent to each sport. In football (soccer), ACL injuries primarily occur in non-contact or indirect contact situations such as defensive pressing actions or regaining balance after kicking, with dynamic knee valgus being commonly reported [9]. Whereas in American Football, ACL injuries predominantly occur because of indirect contact or direct contact to the knee [10].

As outlined by Bahr and Krosshaug [11], the definition of an ACL injury mechanism in sport is multifactorial and can encompass sport-specific actions, whole-body movements at the time of injury, and biomechanical analyses of the knee joint. However, the preventive measures and rehabilitation practice after ACL injury have traditionally been directed towards biomechanical factors, including landing position, knee valgus, and heel strike [12]. To enhance comprehension beyond the biomechanical perspective, situational patterns refer to describing and contextualizing the injury situation with various factors, such as player characteristics (e.g., sex, age, position), player behavior (e.g., aggressiveness, risk taking), opponent behavior (e.g., physicality or foul play), and match characteristics (e.g., scores being level, high stakes or decisive moments) [9, 11]. Different sports manifest distinct patterns of play, including intensity levels, technical complexity, visual-spatial focus, and degree of physical contact with opponents, which all may play roles in identifying potential risk situations for ACL injuries. The divergence in ACL injury mechanisms between sports underscores the importance of sport-specific situational patterns and reinforces the necessity to tailor prevention strategies and rehabilitation practices to the individual athlete, considering the demands and situations specific to their respective sports [13–15]. The distinctions between types of sports may also elucidate the challenges faced in identifying athletes at risk.

As our understanding of the injury mechanism and situational patterns behind ACL injuries continues to expand, there is a need to summarize the existing data. Consequently, the purpose of this systematic review was to compile and synthesize data regarding the injury mechanisms and situational patterns leading to ACL injuries across various sports.

## Methods

This systematic review was reported according to The Preferred Reporting Items for Systematic Review and Meta-Analyses (PRISMA) guidelines [16] and was pre-registered at PROSPERO (CRD42022355173). Compared with the pre-registered PROSPERO protocol, the results presented deviated by not distinguishing between age and primary versus secondary ACL injuries because of insufficient information provided in the included articles.

### Search Strategy and Data Extraction

A comprehensive database search was performed by a medical librarian at the Biomedical Library at the University of Gothenburg using MEDLINE, Embase, Cochrane Library, Allied and Complementary Medicine Database, and the Physiotherapy Evidence Database databases in December 2022 and was repeated in October 2023 to identify additional published articles. The search strategy combined various search terms and their synonyms: “anterior cruciate ligament injuries, situational pattern, mechanism, biomechanical analysis, injury video, and video analysis” (Electronic Supplementary Material [ESM]). Reference lists of pertinent articles and systematic reviews were searched for additional articles by the first and second authors (AS and JH). The search was uploaded onto the Rayyan reference management platform (rayyan.ai) for manual screening of potential articles to include [17]. All articles were initially screened based on their titles and abstracts by the first and second author (AS and JH) to ensure consensus. Articles deemed eligible were further assessed in full text before inclusion. Agreement between the authors was assessed using Cohen’s kappa coefficient, which demonstrated a value of 0.81, suggesting nearly perfect agreement. No disagreement arose that needed discussion with the senior author (EHS).

Data extraction of articles was carried out by the first and second author (AS and JH) using a predesigned Excel spreadsheet (version 16; Microsoft Corporation, Redmond, WA, USA). The extracted data included: first author information, year of publication, article title, journal, study design, purpose, conclusion, number of patients, sex, age, type of sport, level of sport, player experience, player position, primary/secondary ACL injury, methodology to assess ACL injuries, injury mechanism (non-contact, indirect contact or direct contact injury), situational patterns (e.g., pressing or dribbling), movement patterns (e.g., landing or change of direction [COD]), foul committed at the time of injury, injury circumstances (e.g., first or second half, season timeframe), environmental conditions (e.g., weather, surface), and biomechanics of the injury involving ankle, knee, hip, and trunk joint angles. In cases of insufficient information, attempts were made to contact the corresponding authors for clarification.

### Eligibility Criteria

English-language articles that investigated the injury mechanism and situational patterns of ACL injuries were considered for inclusion. All types of observation methods for data collection (video analysis, questionnaires, interviews, and medical records) were accepted and further categorized into athlete- or medical staff-reported data when possible. No restrictions on publication dates were applied. Exclusion criteria comprised systematic reviews, meta-analyses, opinion pieces, editorials, and congress abstracts. Case studies were included when a video analysis was used to elucidate the ACL injury mechanism. In instances when articles included multiple sports but did not present information on the injury mechanism separately for each sport, the corresponding author was contacted by e-mail. If no response was received, articles were excluded.

### Data Synthesis

The data for ACL injury mechanisms, situational patterns, movement patterns, and biomechanics were synthesized in qualitative tables presented separately by sport. To synthesize the collected data, a set of categories within each area (article information, research methods, injury mechanism, situational pattern, movement pattern, injury circumstances, biomechanics) was predetermined. To synthesize the results without altering the reporting of the unique study outcomes, these categories were continuously updated as new data deviated from the predetermined categories. Determination of overlapping categories and descriptions of data was reached through discussion and consensus among the authors (AS, JH, and EHS). Table 1 displays the definitions of the used concepts in the present study. ‘Situational pattern’ represents a comprehensive category to investigate the context surrounding an ACL injury situation; however, several articles only reported the actual movement without the further context of the injury situation, which we presented as ‘movement pattern.’ An ACL injury situation for the same individual could therefore both be reported as ‘defensive pressing’ (situational pattern) and ‘COD’ (movement pattern). Therefore, the category of situational patterns overlaps within and with movement patterns in many of the reported articles, implying that the same individual may be included in both categories and as a result, the presented outcome may fall below or exceed 100%. Skiing, snowboarding, and wakeboarding were not presented with the same definitions of injury mechanisms (non-contact, indirect contact, and direct contact) as they involved equipment attached to the feet which excludes this division. Judo was presented with low or high impact in a contact injury mechanism and a specified judo technique in the injury situation. Whenever feasible, data were presented separately for male and female individuals. In the qualitative summarization in tables, joint angles were presented with mean and standard deviation or median with range. However, in some articles, the authors reported a descriptive term instead of specific joint angles, for example, knee valgus or hyperextension. Furthermore, for both the reported joint angle at initial contact (IC), the foot at initial ground contact, and the injury frame, an estimated timepoint of ACL injury in the subsequent video sequence (range 33–66 ms) was provided. We also performed a quantitative synthesis for common situational patterns observed in football, such as pressing, landing, and kicking. Median values with a 95% confidence interval for ankle, knee, and hip angles in the sagittal plane at the initial contact and at the injury frame for the respective situational patterns were illustrated in box plots. A sex-specific analysis was performed for ACL injuries in football regardless of situational pattern. A quantitative synthesis was also performed for ACL injuries in basketball and presented with mean values with a 95% confidence interval involving ankle, knee, and hip angles in the sagittal plane irrespective of situational pattern.

**Table 1:** Definition of used concepts, adapted from Della Villa et al. [9] with permission

Term	Definition and use
Injury mechanism	This term describes the ACL injury causation, referring to athlete-to-athlete interaction that led to the injury
Non-contact	ACL injury situation without contact from another athlete or object
Indirect contact	ACL injury situation with contact from another athlete or object, but not directly towards the injured knee
Direct contact	ACL injury situation with direct contact to the injured knee from another athlete or object
Contact unspecified	ACL injury situation with contact but not further specified location of contact
Situational pattern	This term describes the athletic action in a sport-specific situation, e.g., offensive/defensive actions and considers the action interacting with the environment in the situation
Movement pattern	This term describes the movement performed leading to an ACL injury, without further information about the situation
Biomechanics of injury	This term refers to the kinematics and the intersegmental position of body segments and joints at initial ground contact (foot at initial ground contact) and the reported frame of the ACL injury situation
Injury circumstances	This term refers to surrounding circumstances that may be significant for the ACL injury situation (e.g., weather, surface, player position, match or training, timepoint of match, period of season)

*ACL* anterior cruciate ligament

The creation of figures containing biomechanical data was performed using MedCalc Software (Acacialaan, Ostend, Belgium). To illustrate situational patterns during ACL injury events in various sports, a four-step presentation was created with Microsoft PowerPoint (Microsoft Corporation, Redmond, WA, USA), according to previous work from Della Villa et al. [9]. This visual material analyzes the situations of ACL injury situations from the initial contact to the injury frame, utilizing previously published material from co-authors (ESM) [9, 18, 19] and video footage available on YouTube (youtube.com). The video footage was downloaded and processed using Adobe Premiere Pro (Adobe Systems, San Jose, CA, USA) to enable video repetition, slow motion, and frame-by-frame navigation. The situational patterns were labeled according to the specific characteristics of each sport and categorized based on the type of contact (non-contact, indirect contact, direct contact) and the phase of play (offensive vs defensive play).

### Article Quality Assessment

The included articles using video analysis methodology were assessed using the Quality Appraisal for Sports Injury Video Analysis Studies (QA-SIVAS) scale [20]. The first and second authors (AS and JH) independently examined the included articles, resolving any discrepancies through discussion. The QA-SIVAS scale consists of an 18-item checklist addressing the article design, data source, article methodology, reporting of results, and appropriate discussion and conclusion of video analysis articles in sports injury research. Each item is to be answered with either 0 (no/not stated) or 1 (yes/present) points for each item. The maximum attainable score is 18, with the quality rating expressed as a percentage (achieved score/maximum score [%]). The QA-SIVAS scale has demonstrated excellent inter- and intra-rater reliability, with an intraclass correlation coefficient of > 0.97 [20].

The articles detailing ACL injuries through athlete/medical staff reports and medical records were subjected to a quality assessment utilizing the JBI Critical Appraisal Checklist for Case Series (JBI checklist) [21], selected for its alignment with the article’s objective. The first and second authors (AS and JH) independently evaluated the quality of the included articles. The JBI checklist comprises ten items, each evaluated as ‘yes, ‘no,’ ‘unclear,’ or ‘not applicable.’ Following discussion and agreement with the senior author (EHS), it was determined that the items ‘Reported outcomes’ and ‘Statistical analysis’ were redundant and not applicable in all instances. Accordingly, a score of ≥ 7 ‘yes’ was considered to indicate high quality, ≥ 5 ‘yes’ denoted moderate quality, and articles scoring lower were regarded as low-quality evidence.

## Results

**Fig. 1:**
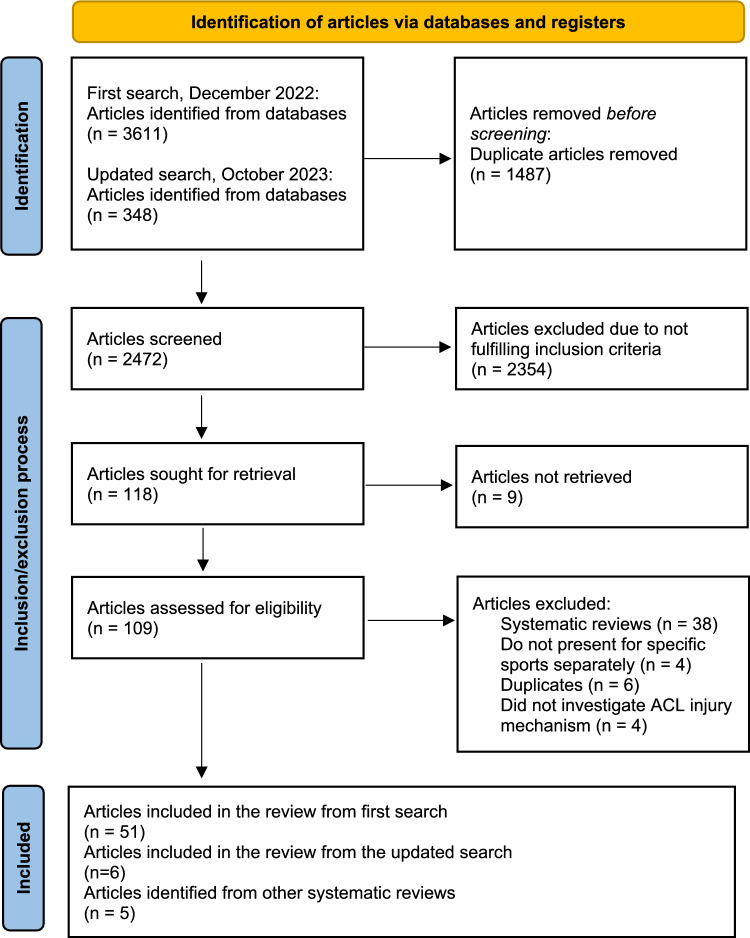
Flowchart of the study inclusion/exclusion process. *ACL* anterior cruciate ligament

Sixty-two articles that covered 20 different sports were included in the present systematic review. Among the included articles, 30 used video analysis, 25 used athlete-reported data, and 7 relied on reports from medical staff or medical records. Figure 1 demonstrates the inclusion and exclusion process.

### Article Quality Assessment

**Fig. 2:**
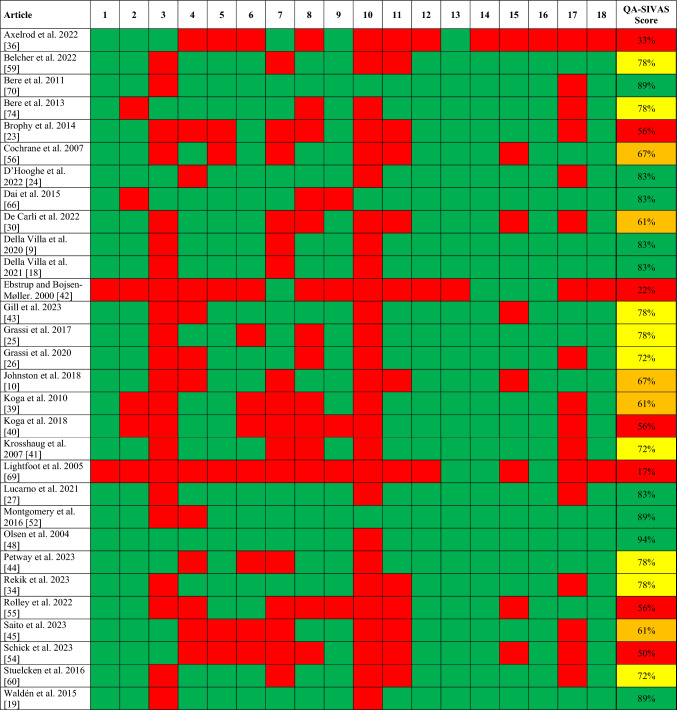
Article quality assessment by the Quality Appraisal for Sports Injury Video Analysis Studies (QA-SIVAS) scale. Item 1. Objective; 2. Sample information; 3. Sample characteristics; 4. Video quality; 5. Applied methods; 6. Systematic approach; 7. Medical information; 8. Rater expertise; 9. Number of raters; 10, Control group; 11. Quantitative biomechanical analysis; 12. Main results; 13. Numbers and proportions; 14. Injury context; 15. Example screenshot; 16. Discussion; 17. Clinical implications; 18. Limitations. QA-SIVAS scale item assessment: green, yes; red, no. QA-SIVAS scale total score: red, low quality (< 60%); amber, moderate quality (60–70%); yellow, good quality (70–80%); green, high quality (> 80%)

**Fig. 3:**
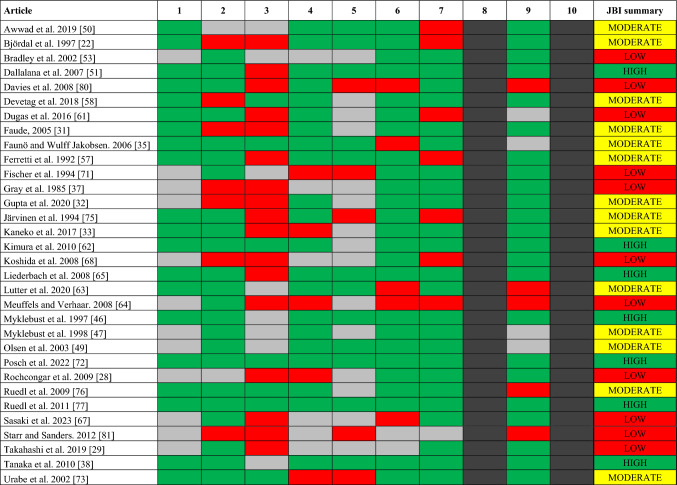
Article quality assessment with the JBI Critical Appraisal Checklist for Case Series (JBI checklist) tool. Item 1. Inclusion criteria; 2. Reliable measurement; 3. Valid measurement; 4. Consecutive inclusion; 5. Complete inclusion; 6. Participant demographics; 7. Clinical Information; 8. Outcome reporting; 9. Clinical demographics; 10. Statistical analysis. JBI checklist item assessment: green, yes; red, no; gray, unclear; black, non-applicable. JBI checklist summary: red, low quality (< 5); yellow, moderate quality (5–6); green, good quality (≥ 7)

All 30 video analysis articles were assessed for quality using the QA-SIVAS scale. Seven articles (23%) scored below 60%, indicating low quality, while five articles (17%) scored between 60 and 70%, classifying them as moderate quality. Nine articles (30%) scored between 71 and 80%, reflecting good quality, and nine articles (30%) scored between 81 and 100%, indicating high quality (Fig. 2). The most common limitations were the absence of describing the characteristics of the population (item 3) and the absence of a control group (item 10). The 32 articles documenting ACL injuries reported from medical records, athlete- and medical staff-reported data were evaluated for quality using the JBI checklist. Of the 32 articles, 7 articles (22%) were classified as high quality, 14 (44%) as moderate quality, and the remaining 11 (34%) articles were designated as low quality (Fig. 3).

### Team Ball Sport

#### Football (Soccer)

**Fig. 4:**
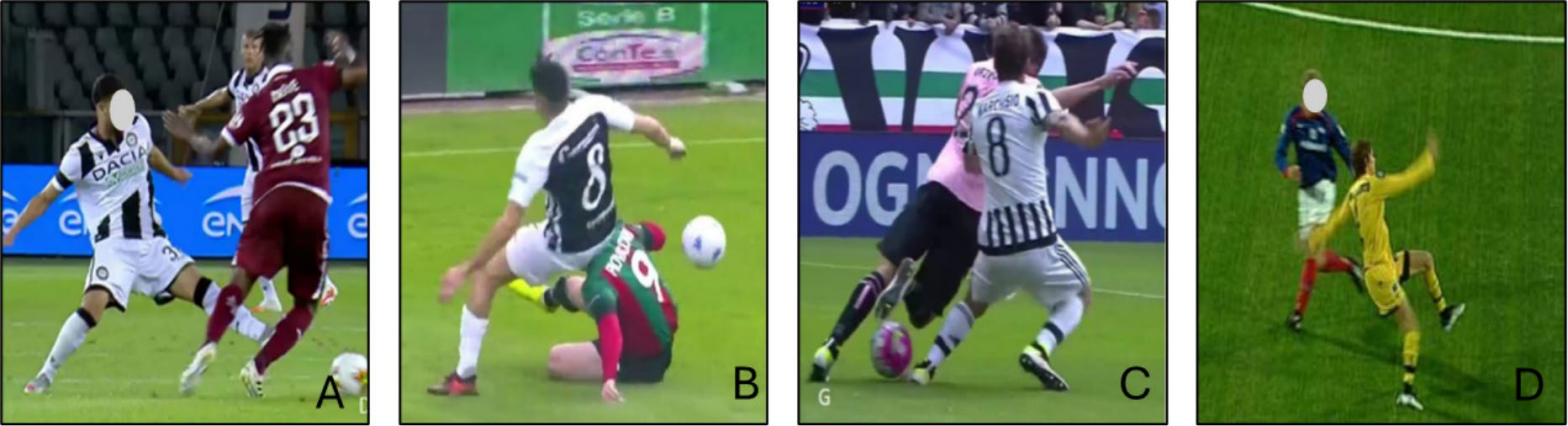
Situational patterns of ACL injuries in football. (**A**) defensive pressing, (**B**) being tackled, (**C**) tackling, and (**D**): regaining balance after kicking. Reproduced with permission from the publishers of the original articles by Della Villa et al. [9] and Waldén et al. [19]

**Fig. 5:**
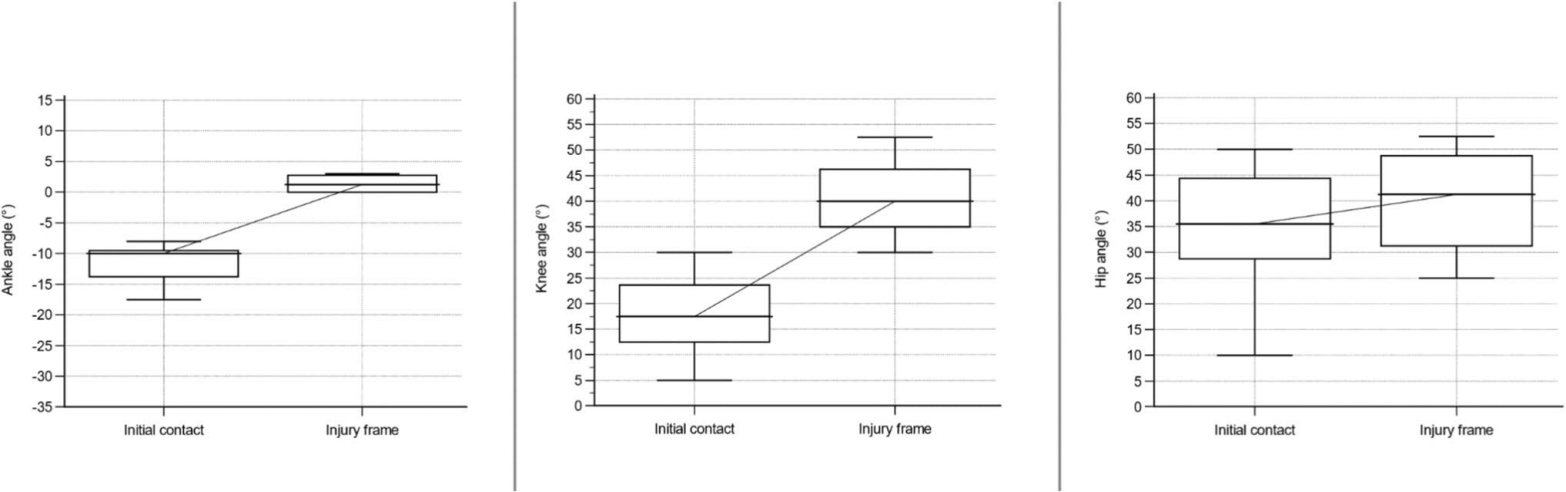
Angle of ankle, knee, and hip in a sagittal plane for sustaining an anterior cruciate ligament injury in football regardless of situation. Based on D’Hooghe et al. [24], Della Villa et al. [9], Lucarno et al.[27], Rekik et al. [34], and Waldén et al. [19]. Presented as median values with a 95% confidence interval. Positive values for the ankle indicate dorsiflexion and negative plantar flexion

**Fig. 6:**
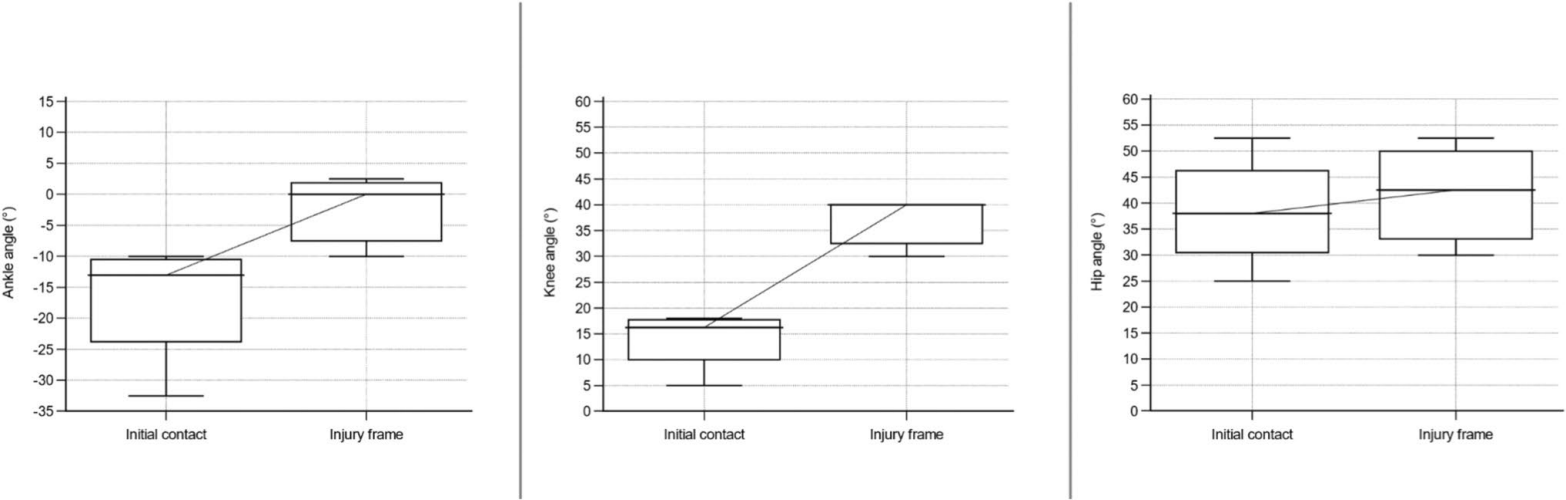
Angle of ankle, knee, and hip in a sagittal plane for sustaining an anterior cruciate ligament injury in football during a pressing situation. Based on D’Hooghe et al. [24], Della Villa et al. [9], Rekik et al. [34], and Waldén et al. [19]. Presented as median values with a 95% confidence interval. Positive values for the ankle indicate dorsiflexion and negative plantar flexion

**Fig. 7:**
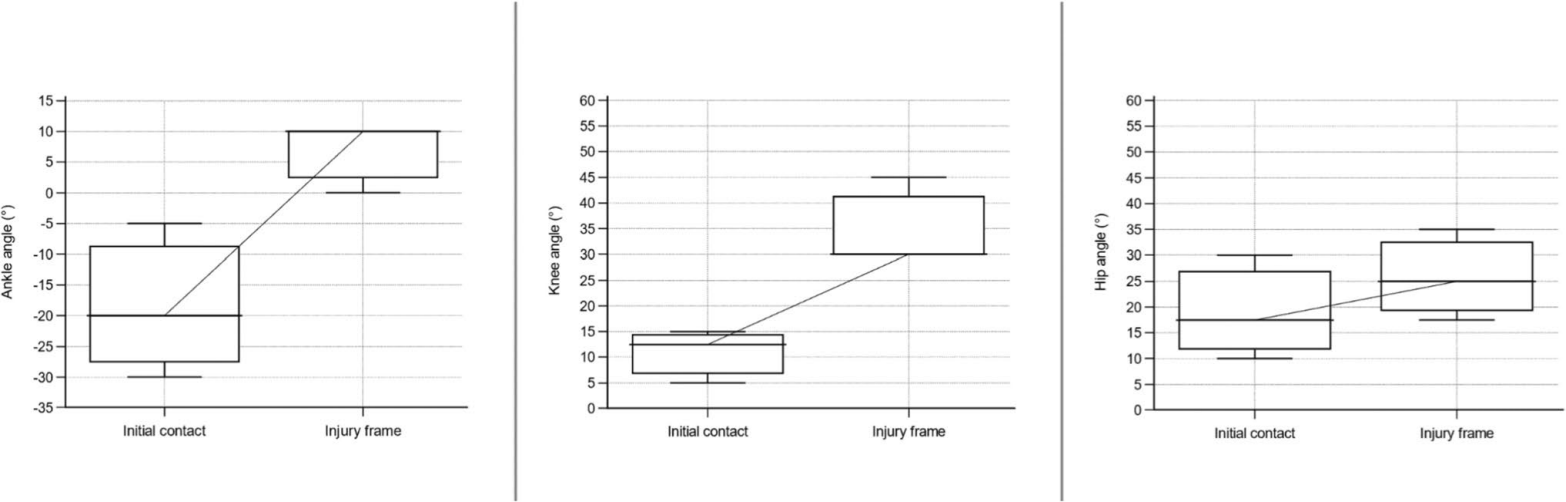
Angle of ankle, knee, and hip in a sagittal plane for sustaining an anterior cruciate ligament injury in football during a landing situation. Based on D’Hooghe et al. [24], Della Villa et al. [9], and Waldén et al. [19]. Presented as median values with a 95% confidence interval. Positive values for the ankle indicate dorsiflexion and negative plantar flexion

**Fig. 8:**
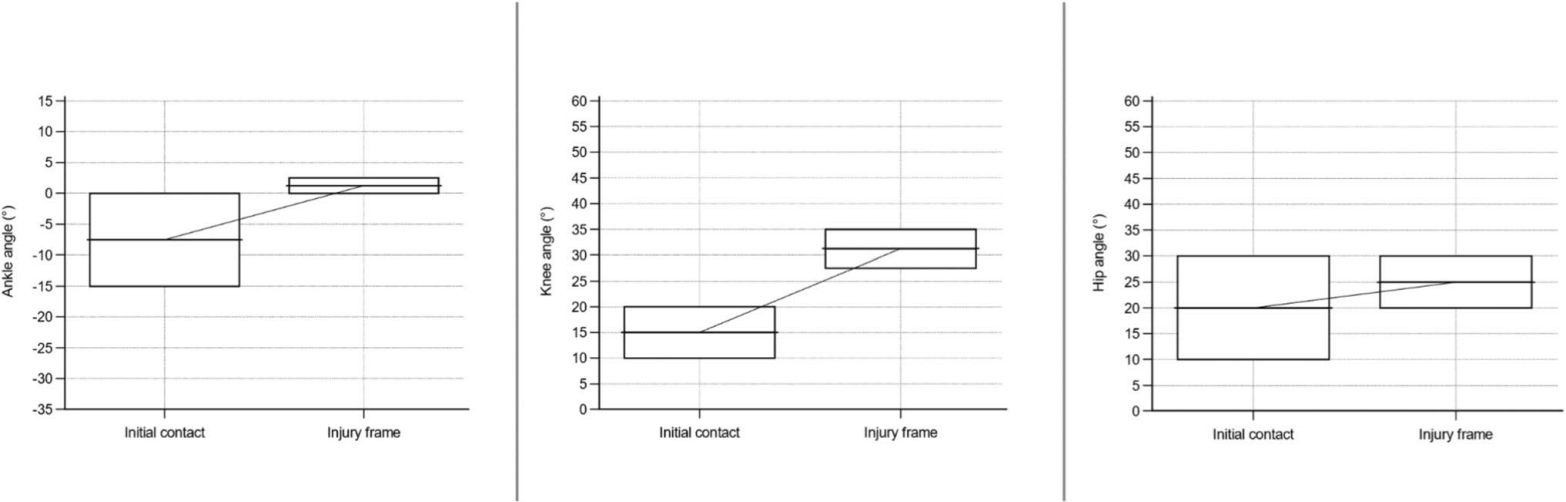
Angle of ankle, knee, and hip in a sagittal plane for sustaining an anterior cruciate ligament injury in football during a kicking situation. Based on Della Villa et al. [9] and Waldén et al. [19]. Presented as median values with a 95% confidence interval. Positive values for the ankle indicate dorsiflexion and negative plantar flexion

**Fig. 9:**
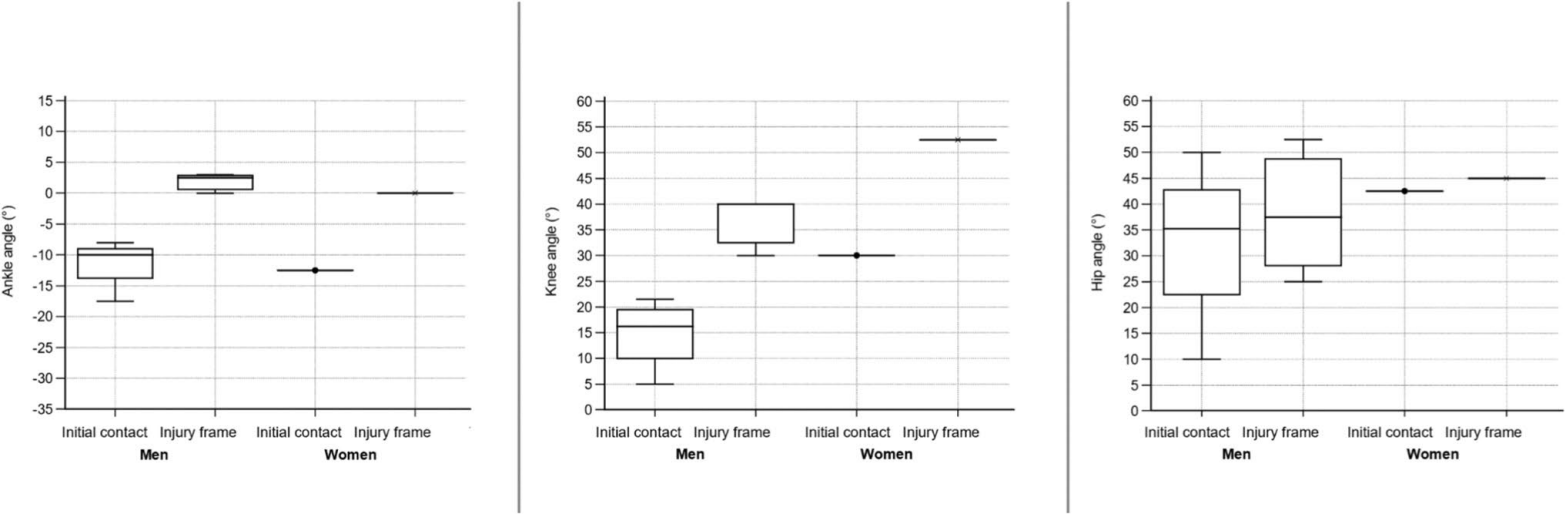
Angle of ankle, knee, and hip in a sagittal plane for sustaining an anterior cruciate ligament injury in football specified for sex. Based on D’Hooghe et al. [24], Della Villa et al. [9], Rekik et al. [34], and Waldén et al. [19] for men, and Lucarno et al. [27] for women. Presented as median values with a 95% confidence interval. Positive values for the ankle indicate dorsiflexion and negative plantar flexion

Sixteen articles investigated ACL injuries in football, including 2178 individuals, among whom 1652 were male individuals, 526 female individuals [9, 19, 22–34], and 105 individuals were unspecified [35]. The most common situational patterns for ACL injury in football involved rapid deceleration or sudden COD, particularly during defensive pressing actions, tackling and player in possession being tackled. These movements often occurred in medium- to high-speed scenarios with external attention focused on the opponent or ball (Fig. 4 and ESM). Additionally, a smaller number of ACL injuries were related to landing mechanics, especially among goalkeepers who sustained injuries while landing from a jump or during collisions [25]. The most common biomechanical pattern (68–72%) involved hip abduction, external foot rotation, and heel strike; see ESM and Figs. 5, 6, 7, 8, 9 for detailed information on the biomechanics of ACL injuries in football.

The proportion of ACL injuries during matches and training varied, where Kaneko et al. [33] reported 62% during matches and 32% during training sessions, and Gupta et al. [32] reported 13–22% of injuries during match play. Most articles indicated a higher proportion of ACL injuries in the first half compared with the second half in matches. Among the four articles that examined the timepoint of ACL injury, the distribution between the first and second half was: 62% versus 38% [9], 79% versus 21% [25], and 55% versus 45% [27], and Faunø and Wulff Jakobsen [35] reported an inverse relationship of 38% versus 62%. A foul was committed in 10–33% of ACL injury situations [6, 30]. When only direct and indirect contact ACL injuries were considered, foul play was reported in 58% of the cases—of which 73% were in favor of the injured player and 27% were against [25]. Four articles recorded the weather conditions at the time of ACL injury, with precipitation in only 3% of injury situations and dry conditions in the remaining cases [9, 19, 27, 34]. Two articles [19, 27] reported the middle third of the pitch as the most common area for ACL injury (37% and 42%, respectively), while injuries in the defensive third (29% and 33%) and offensive third (34% and 24%) were evenly distributed. Regarding player position, midfielders and strikers were most frequently affected by ACL injuries. Despite most ACL injuries occurring among offensive players, several articles reported that the most common situational pattern for ACL injury was defensive pressing actions (26–40%). Tables 2 and 3 display additional details on ACL injuries sustained in football.

**Table 2:** Video analysis articles of ACL injuries in football

	Brophy et al. 2014 [23]	Waldén et al. 2015 [19]	Grassi et al. 2017 [25]	Della Villa et al. 2020 [9]	Grassi et al. 2020 [26]	Lucarno et al. 2021 [27]	De Carli et al. 2022 [30]	D’Hooghe et al. 2023 [24]	Rekik et al. 2023 [34]
Individuals, *n* (female %)	55 (42%)	39 (0%)	34 (0%)	134 (0%)	21 (0%)	35 (100%)	128 (0%)	19 (0%)	15 (0%)
Level of sport	Various levels	Professional	Professional	Professional	Professional	Professional	Professional	Professional	Professional
ACL injuries reported from match/training	Match	Match	Match	Match	Match	Match	Match	Match	Match
Injury mechanism, *n* (%)									
Non-contact	M: 15 (47%) F: 9 (39%)	25 (64%)	15 (44%)	59 (44%)	16 (76%)	19 (54%)	50 (39%)	9 (47%)	8 (53%)
Indirect contact		8 (21%)	7 (21%)	59 (44%)	5 (24%)	12 (34%)	36 (28%)	6 (32%)	4 (27%)
Direct contact		6 (15%)	12 (35%)	16 (12%)	0 (0%)	4 (12%)	36 (28%)	4 (21%)	3 (20%)
Contact unspecified	M: 17 (53%) F: 14 (61%)						6 (5%)		
Additional details on injury mechanism, *n* (%)									
Weightbearing 1 leg		34 (87%)	20 (59%)	94 (70%)		33 (94%)		14 (74%)	
Weightbearing 2 leg		5 (13%)		29 (30%)				5 (26%)	
Horizontal velocity, high,		17 (52%)		78 (58%)		22 (63%)	77 (60%)	15 (79%)	
Horizontal velocity, low		14 (42%)		49 (37%)		12 (34%)	37 (29%)	2 (11%)	
Horizontal velocity, zero		2 (6%)		6 (5%)		1 (3%)	3 (2%)	2 (11%)	
Horizontal velocity, unknown							11 (9%)		
Vertical velocity, high		3 (9%)		9 (7%)		4 (11%)		1 (5%)	
Vertical velocity, low		3 (9%)		49 (37%)		11 (31%)		1 (5%)	
Vertical velocity, zero		27 (82%)		75 (56%)		20 (57%)		17 (90%)	
Phase of play, *n* (%)									
Attacking/ball possession	M: 12 (38%) F: 3 (13%)	9 (23%)	17 (50%)	43 (32%)		11 (31%)	35 (40%)^a^	6 (32%)	3 (20%)
Defending	M: 20 (62%) F: 20 (87%)	30 (77%)	17 (50%)	91 (68%)		24 (69%)	68 (53%)	13 (68%)	12 (80%)
Counterattack							7 (5%)		
Situational pattern, *n* (%)									
Defensive pressing		11 (28%)	9 (26%)	40 (30%)		14 (40%)	40 (31%)	7 (37%)	6 (40%)
Dribbling		1 (3%)					11 (9%)		1 (7%)
Ball protection/control							20 (16%)		
Tackling	M: 13 (41%) F: 15 (65%	6 (15%)	4 (12%)	15 (11%)		4 (13%)	16 (13%)	1 (5%)	2 (13%)
Being tackled	M: 4 (13%) F: 0 (0%)	3 (8%)	11 (32%)	24 (18%)		4 (13%)		1 (5%)	2 (13%)
Blocking									4 (27%)
Collision			3 (9%)						
Regaining balance after kicking	M: 3 (9%) F: 1 (4%)	5 (13%)	7 (21%)	19 (14%)		7 (23%)	13 (10%)	1 (5%)	
Receiving pass		2 (5%)		2 (1%)			5 (4%)		
Heading	M: 3 (9%) F: 0 (0%)	5 (13%)							
Movement pattern, *n* (%)									
Single-leg landing		4 (10%)			7 (33%)				
Bilateral landing		1 (3%)							
Landing, unspecified	M: 6 (19%) F: 3 (13%)	5 (13%)		8 (6%)		1 (3%)	31 (24%)	1 (5%)	
Changing direction/cutting	M: 9 (28%) F: 7 (30%)	10 (26%)			14 67%)		23 (18%)	10 (53%)	
Decelerating during running	M: 3 (9%) F: 8 (35%)						45 (35%)		
Planting, unspecified	M: 6 (19%) F: 3 (13%)							4 (21%)	
Direct blow to the knee		6 (15%)						4 (21%)	

Situational pattern overlaps within and with movement patterns, which means that the same individual can be included in both categories and the presented result may fall below or exceed 100%

*ACL* anterior cruciate ligament, *F* female, *M* male, *n* number of individuals

^a^Includes game construction, *n* = 16 (13%)

**Table 3:** Medical records/questionnaire/interviewing of ACL injuries in football

	Björdal et al. 1997 [22]	Faude et al. 2005 [31]	Faunø and Wulff Jakobsen 2006 [35]	Gupta et al. 2020 [32]	Kaneko et al. 2017 [33]	Rochcongar et al. 2009 [28]	Takahashi et al. 2019 [29]
Individuals, *n* (female %)	176 (24%)	11 (100%)	105 (sex unspec-ified)	277 (73%)	90 (100%)	934 (0%)	200 (50%)
Level of sport	Various levels	Professional	Professional 14%, recreational 86%	High school	University and youth teams	Various levels	High school
ACL injuries reported from match/training	Both	Both	Both	Both	Both	Both	Not reported
Injury mechanism, *n* (%)							
Non-contact	95 (54%)	7 (64%)	88 (84%)	M: 27 (35%) F: 106 (53%)	55 (61%)	603 (78%)	M: 41 (41%) F: 53 (53%)
Indirect contact					14 (16%)		M: 28 (28%) F: 26 (26%)
Direct contact					21 (23%)		M: 27 (27%) F: 10 (10%)
Contact unspecified	81 (46%)	4 (36%)	17 (16%)	M: 37 (48%) F: 61 (30%)		175 (22%)	
Phase of play, *n* (%)							
Attacking	122 (69%)		50 (48%)		36 (40%)		
Defending	54 (31%)		38 (36%)		54 (60%)		
Situational pattern, *n* (%)							
General play				M: 7 (18%) F: 26 (24%)			
Defending				M: 7 (18%) F: 23 (22%)			
Pressing					29 (32%)		
Tackling		3 (27%)				16 (2%)	
Being tackled	106 (60%)	1 (9%)				88 (11%)	
Player-player contact				M: 11 (27%) F: 30 (28%)			
Stepped on/fallen on/kicked				M: 4 (10%) F: 4 (4%)			
Loose ball				M: 7 (18%) F: 23 (22%)	6 (7%)		
Running with the ball	100 (57%)		58 (55%)				
Dribbling				M: 5 (13%) F: 10 (10%)	14 (16%)		
Receiving				M: 4 (10%) F: 7 (7%)	11 (12%)		
Kicking				M: 6 (15%) F: 12 (13%)	9 (10%)	15 (2%)	
Pass cutting					4 (4%)		
Heading				F: 3 (3%)	3 (3%)		
Goalkeeping					4 (4%)		
Sliding					2 (2%)		
Movement pattern, *n* (%)							
Changing direction/cutting		7 (64%)			14 (16%)	109 (14%)	M: 13 (13%)^a^ F: 21 (21%)
Decelerating					22 (24%)		
Pivoting				M: 17 (43%) F: 52 (48%)		267 (34%)	
Landing, unspecified			26 (25%)		12 (13%)	159 (20%)	M: 8 (8%)^a^ F: 6 (6%)
Direct trauma						71 (9%)	

Situational pattern overlaps within and with movement patterns, which means that the same individual can be included in both categories and the presented result may fall below or exceed 100%

*ACL* anterior cruciate ligament, *F* female, *M* male, *n* number of individuals

^a^Non-contact injury only considered

#### Basketball

**Fig. 10:**
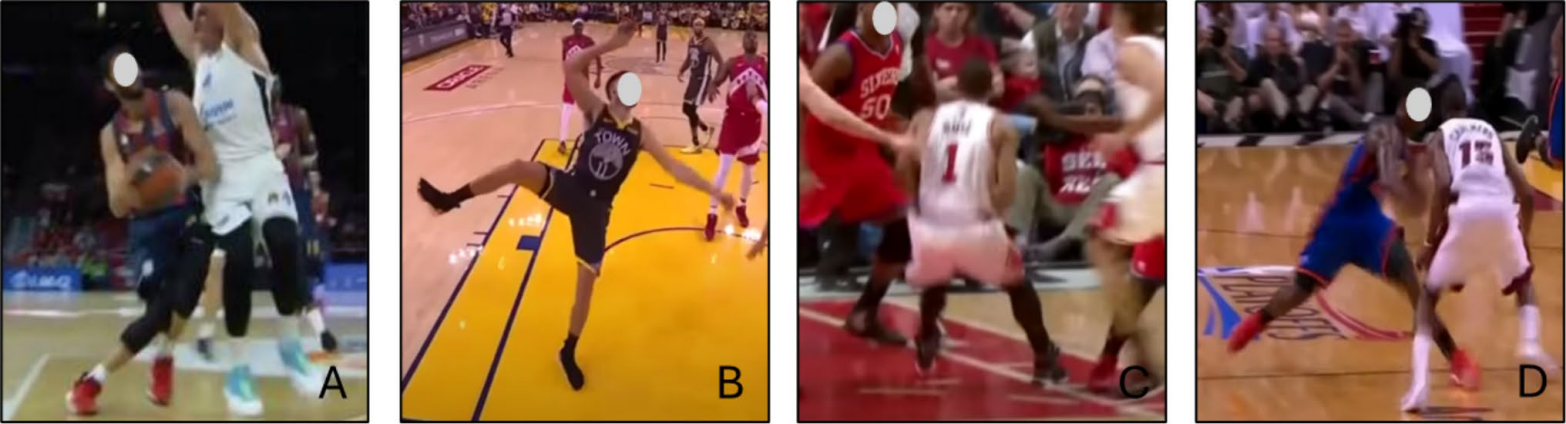
Situational patterns of anterior cruciate ligament injuries in basketball. (**A**) attacking the basket, (**B**) single-leg landing, (**C**) jump stop (bilateral landing), and (**D**) offensive change of direction. Image created by the authors from publicly available video footage (Youtube.com)

**Fig. 11:**
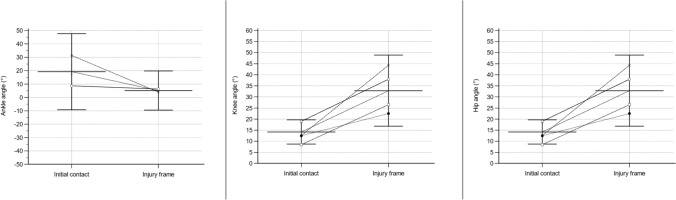
Angle of ankle, knee, and hip in a sagittal plane for sustaining an anterior cruciate injury in basketball. Presented as average values with a 95% confidence interval. Based on Koga et al. [39, 40], Krosshaug et al. [41], Petway et al. [44], and Gill et al. [43]. Positive values for the ankle indicate dorsiflexion and negative plantar flexion

Eleven articles investigated ACL injuries in basketball players, comprising 408 ACL injuries, with 199 occurring in female players and 209 in male players [29, 36–45]. Point guards and shooting guards were reported as the most ACL injury-prone positions, with a range from 54 to 74%, followed by forwards at 16–27%, while 3% of the ACL injuries were to centers [36, 43, 45]. Non-contact situations with an opponent in proximity or indirect contact during an offensive COD or landing after a jump were reported as the most common ACL injury mechanism in basketball (Table 4). The first gather step following picking up the ball and attacking the rim was the most frequent situational pattern (34%), often with indirect contact from another player to the contralateral side of the body to the injured knee. Other common situational patterns of ACL injury were ‘jump stop,’ a bilateral landing to reduce the forward momentum preceding a shot, and a single-legged landing after rebound or attacking the rim, with upper body contact and center of mass outside the base of support at landing (Fig. 10 and ESM) [43–45]. Table 4 displays additional details on ACL injuries sustained in basketball. For a detailed presentation of the biomechanics of ACL injuries in basketball, see Fig. 11 and ESM.

**Table 4:** Anterior cruciate ligament injuries in basketball

	Gray et al. 1985 [37]	Ebstrup and Bojsen-Möller 2000 [42]	Krosshaug et al. 2007 [41]	Tanaka et al. 2010 [38]	Koga et al. 2010 [39] and 2018 [40]	Takahashi et al. 2019 [29]	Axelrod et al. 2022 [36]	Gill et al. 2023 [43]	Petway et al. 2023 [44]	Saito et al. 2023 [45]
Methodology	Medical staff report	Video analysis	Video analysis	Medical records	Video analysis	Medical records	Video analysis	Video analysis	Video analysis	Video analysis
Individuals, *n* (female %)	24 (100%)	1 (100%)	39 (56%)	6 (100%)	3 (100%)	200 (50%)	10 (100%)	38 (0%)	27 (0%)	27 (0%)
Level of sport	Various levels	Professional	Various levels	Various levels	Professional	High school	Professional	Professional	Professional	Professional
Injury mechanism, *n* (%)										
Non-contact		1 (100%)	M: 9 (53%) F: 13 (59%)		1 (33%)	M: 69 (69%) F: 65 (65%)	9 (90%)	9 (27%)	5 (18%)	4 (27%)^d^
Indirect contact			M: 1 (6%) F: 6 (27%)		2 (67%)	M: 18 (18%) F: 23 (23%)		29 (73%)	21 (78%)	11 (73%)^d^
Direct contact			M: 4 (23%) F: 0 (0%)	P: 2 (33%) S: 1 (17%)		M: 10 (10%) F: 8 (8%)	1 (10%)			
Unspecified			M: 3 (18%) F: 3 (14%)	P. 4 (67%) S: 5 (83%)		M: 3 (3%) F: 4 (4%)			1 (4%)	
Phase of play, *n* (%)										
Attacking			29 (74%)					32 (84%)		22 (82%)
Defending		1 (100%)	5 (13%)					3 (8%)		2 (7%)
Rebound			2 (5%)					2 (5%)		3 (11%)
Situational pattern, *n* (%)										
Ball possession (dribble move)			28 (72%)		2 (67%)			2 (5%)		3 (11%)
Gather step								16 (42%)^b^		15 (56%)
Jump step								5 (13%)		
Turnover			1 (3%)							
Had just passed/shot the ball			3 (8%)		1 (33%)					
Defensive play or other			7 (17%)					1 (3%)		5 (18%)
Movement pattern										
Single-leg landing			M: 6 (35%) F: 4 (18%)		1 (33%				6 (23%)	
Bilateral landing			M: 4 (24%) F: 9 (41%)						8 (29%)	
Landing unspecified	14 (58%)			P: 2 (33%) S: 1 (17%)		M: 27 (27%)^a^ F: 18 (18%)	4 (40%)	14 (37%)		4 (15%)
Change of direction	9 (38%)	1 (100%)	M: 2 (12%) F: 2 (9%)	S: 3 (50%)	2 (67%)	M: 20 (20%)^a^ F: 33 (33%)	6 (60%)	23 (61%)	13 (48%)^c^	18 (67%)
Deceleration				P: 2 (33%)						
Direct blow to knee			M: 4 (24%) F: 0 (0%)	P: 1 (17%) S: 2 (33%)						

Situational pattern overlaps within and with movement patterns, which means that the same individual can be included in both categories and the presented result may fall below or exceed 100%

*F* female, *M* male, *n* number of individuals, *P* primary anterior cruciate ligament injury, *S* secondary anterior cruciate ligament injury

^a^Non-contact injuries considered only

^b^Including first step 13 (34%) [‘traditional drive’ *n* = 8, ‘euro step’ *n* = 4, ‘unclear’ *n* =]) and second step 3 (8%) [‘traditional drive’ *n* = 2, ‘unclear’ *n* = 1]

^c^During single-leg casting

^d^Only presented for the offensive two-step action

#### Handball

**Fig. 12:**
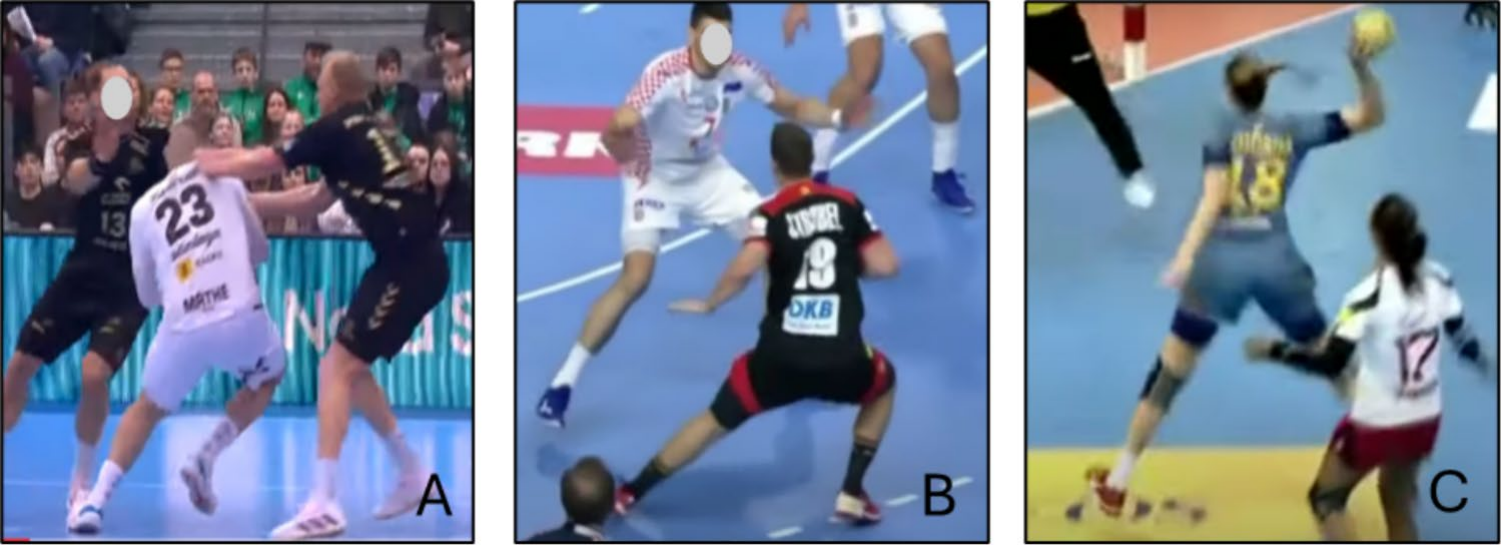
Situational patterns of anterior cruciate ligament injuries in handball. (**A**) Offensive breakthrough, (**B**) step cut maneuver, and (**C**) landing. Images created by the authors from publicly available video footage (Youtube.com)

Eight articles investigated ACL injuries in handball and included 404 individuals, of which 147 were male and 257 were female [29, 39, 40, 42, 46–49]. The most common situational pattern for suffering an ACL injury in handball was a non-contact breakthrough or step cut situation while attacking (71–100%), typically involving a COD with the ball to evade an opponent (Fig. 12 and ESM). The second most common situation reported was a single-leg landing after shooting (14–50%). Table 5 displays additional details on ACL injury mechanism in handball and the ESM presents details of biomechanics.

**Table 5:** ACL injuries in handball

	Myklebust et al. 1997 [46]	Myklebust et al. 1998 [47]	Ebstrup and Bojsen-Möller 2000 [42]	Olsen et al. 2003 [49]	Olsen et al. 2004 [48]	Koga et al. 2010 [39] and 2018 [40]	Takahashi et al. 2019 [29]
Methodology	Athlete reported	Athlete reported	Video analysis	Athlete reported	Video analysis	Video analysis	Medical records
Individuals, *n* (female %)	87 (62%)	28 (82%)	2 (100%)	53 (83%)	20 (100%)	7 (100%)	200 (50%)
Level of sport	Semi-professional	Professional	Professional	Various levels	Professional	Professional	High school
ACL injuries reported from match/training	Both	Both	Match	Match	Match	Match	Not reported
Injury mechanism, *n* (%)							
Non-contact	83 (95%)	25 (89%)	1 (50%)	37 (70%)	13 (65%)	3 (43%)	M: 68 (68%) F: 69 (69%)
Indirect contact			1 (50%)		6 (30%)	4 (57%)	M: 20 (20%) F: 17 (17%)
Direct contact		3 (11%)			1 (5%)		M: 12 (12%) F: 10 (10%)
Contact unspecified				16 (30%)			
Additional details on injury mechanism, *n* (%)							
Speed of movement, very high					Very high 12 (60%)		
Speed of movement, high	46 (53%)	17 (61%)			High 4 (20%)		
Speed of movement, moderate/slow	29 (33%)				Moderate 4 (20%)		
Speed of movement, standing still	12 (14%)						
Phase of play, *n* (%)							
Attacking	78 (90%)	26 (93%)		41 (77%)			
Defending	9 (10%)	2 (7%)		12 (23%)			
Situational pattern, *n* (%)							
Attacking with ball possession	68 (78%)	26 (93%)	2 (100%)	37 (70%)	19 (95%)	5 (71%)	
Regaining balance after shooting						2 (29%)	
Offensive running without ball				4 (8%)			
Defending					1 (5%)		
Movement pattern, *n* (%)							
Single-leg landing			1 (50%)		4 (20%)	2 (29%)	
Landing, unspecified	26 (30%)	4 (14%)		16 (30%)			M: 25 (25%)^a^ F: 30 (30%)
Change of direction	48 (55%)	19 (68%)	1 (50%)	15 (28%)	12 (60%)	5 (71%)	M: 29 (29%)^a^ F: 13 (13%)
Deceleration					2 (10%)		
Direct blow to the knee					1 (5%)		

Situational pattern overlaps within and with movement patterns, which means that the same individual can be included in both categories and the presented result may fall below or exceed 100%

*ACL* anterior cruciate ligament, *F* female,* M* male,* n n*umber of individuals

^a^Only non-contact injuries considered

Studies that used athlete or medical staff reports presented a higher percentage of non-contact ACL injuries (68–95%) compared with studies that used the video analysis where the ratio between non-contact and indirect contact ACL injuries was more evenly distributed (43–65% vs 30–57%). Myklebust et al. [46, 47] reported that ACL injuries occurred more frequently during match play than training with 75% versus 25%, and 86% versus 14%, respectively. Myklebust et al. [46] also specified that 53% of ACL injuries occurred during the first half, and 47% during the second half. Most ACL injuries were sustained by back players at 54%, followed by wing players 30%, line players 5%, and goalkeepers 11% [46]. Olsen et al. [48, 49] reported similar results, where all ACL injuries were sustained by either back or wing players. Three articles reported the type of floor with 58–77% ACL injuries on an artificial floor and 25–42% on a wooden floor (53, 55, 56).

#### Rugby

**Fig. 13:**
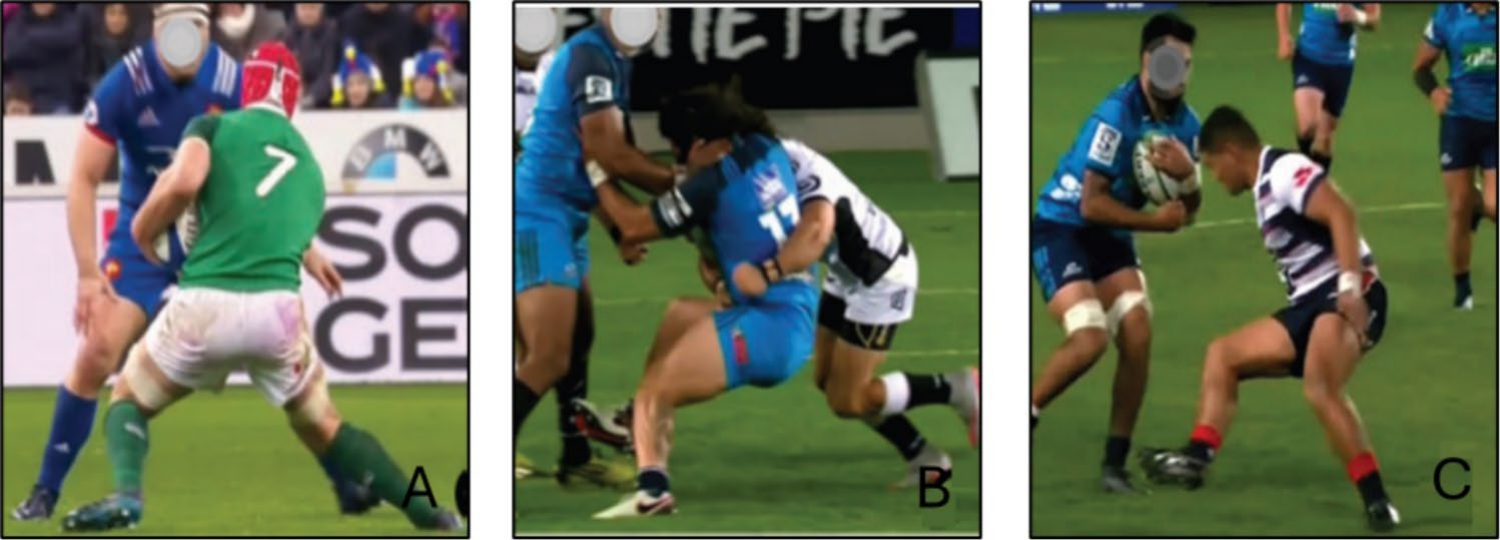
Situational patterns of anterior cruciate ligament injuries in rugby. (**A**) Offensive change of direction, (**B**) being tackled, and (**C**) defensive pressing. Reproduced with permission from the publisher of the original article by Della Villa et al. [18]

Four articles investigated ACL injuries in rugby and included 105 professional male players. [18, 50–52]. Most non-contact ACL injuries were a result of an offensive COD maneuver, such as side-stepping (Fig. 13 and ESM). Concerning contact injuries, rucking and scrummaging are unique to rugby, and these two situational patterns accounted for 8% of ACL injuries (Table 6). Articles that analyzed medical staff reports [50, 51] reported a greater extent of direct contact ACL injuries (67–86%), in comparison with the articles that used a video analysis that reported that non-contact ACL injuries were the most common (42–43%) [18, 52]. Della Villa et al. [18] reported that 57% of ACL injuries occurred during the first half, while 43% occurred during the second half. In contrast, Dallalana et al. [51] reported the second half as the part of the match with the highest incidence of ACL injuries (28% in the first half vs 72% in the second half), while Montgomery et al. [52] reported an equal distribution between the two halves (47% and 53%, respectively). Montgomery et al. [52] reported that the center and hooker were the most affected player positions. Table 6 displays additional details on ACL injury mechanism in rugby and the ESM presents details of the biomechanics of ACL injuries in rugby.

**Table 6:** ACL injuries in rugby

	Dallalana et al. 2007 [51]	Montgomery et al. 2018 [52]	Awwad et al. 2019 [50]	Della Villa et al. 2021 [18]
Methodology	Medical staff reported	Video analysis	Medical staff reported	Video analysis
Individuals, *n* (female %)	9 (0%)	36 (0%)	3 (0%)	57 (0%)
Level of sport	Professional	Professional	Professional	Professional
ACL injuries reported from match/training	Both	Match	Both	Match
Injury mechanism, *n* (%)				
Non-contact	1 (14%)	15 (43%)	1 (33%)	24 (42%)
Indirect contact		8 (23%)		15 (26%)
Direct contact		10 (29%)		18 (32%)
Contact unspecified	8 (86%)	2 (6%)	2 (67%)	
Unknown		1 (3%)		
Additional details on injury mechanism, *n* (%)				
Weightbearing one leg only		16 (44%)		35 (61%)
Horizontal high speed		19 (54%)		27 (47%)
Horizontal low speed		11 (31%)		11 (19%)
Phase of play, *n* (%)				
Attacking		22 (63%)		41 (72%)
Defending		14 (37%)		16 (28%)
Situational pattern (non-contact and indirect contact), *n* (%)				
Offensive change of direction	1 (14%)	8 (22%)		18 (32%)
Defensive pressing		3 (8%)		6 (11%)
Tackling (indirect)		3 (8%)		2 (4%)
Being tackled		3 (8%)		10 (18%)
Landing from a jump				3 (5%)
Rucking		2 (6%)		
Jumping/kneeling/kicking			1 (33%)	
Situational pattern (direct contact), *n* (%)				
Being tackled	4 (43%)	5 (14%)	1 (33%)	10 (18%)
Tackling	3 (29%)	1 (3%)	1 (33%)	5 (9%)
Rucking		2 (6%)		3 (5%)
Scrummaging		1 (3%)		
Set play		1 (3%)		
Kicking		1 (3%)		
Collision	1 (14%)			

*ACL* anterior cruciate ligament, *n* number of individuals

#### American Football

**Fig. 14:**
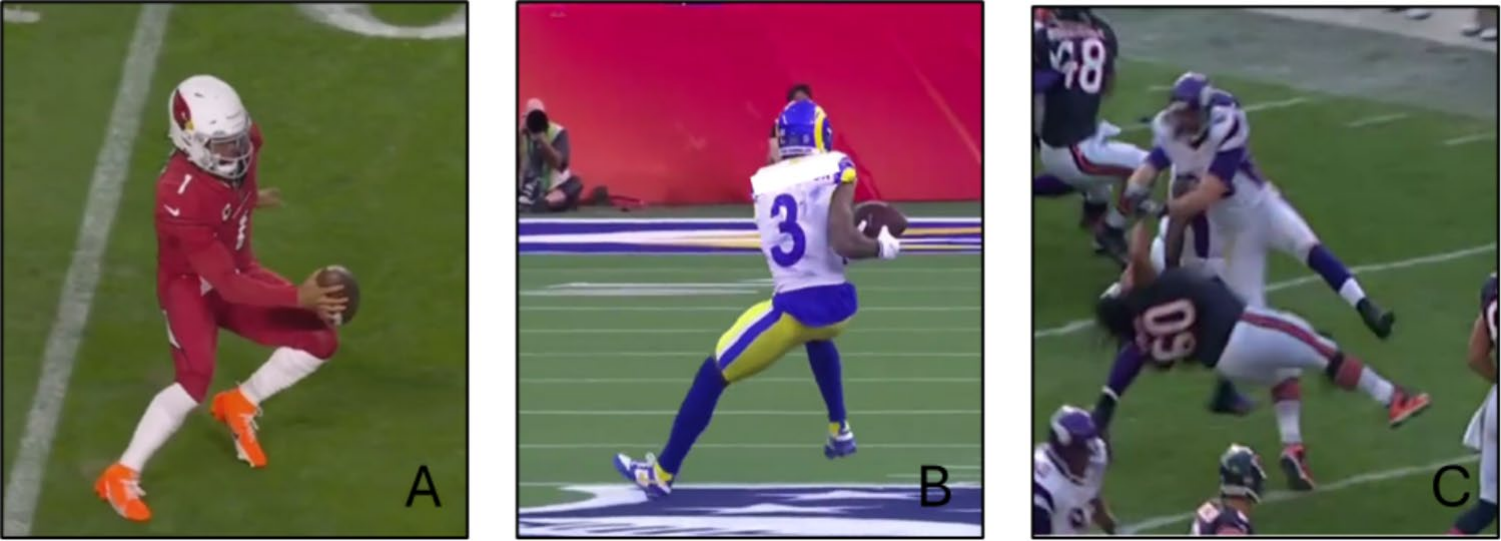
Situational patterns of anterior cruciate ligament injuries in American Football. (**A**) offensive change of direction, (**B**) deceleration from sprinting, and (**C**) being blocked. Images created by the authors from publicly available video footage (Youtube.com)

Three articles investigated ACL injuries in American Football [10, 53, 54], and included 418 male professional athletes. Of the ACL injuries investigated with video analysis (*n* = 122) [10, 54], 83 (68%) occurred as non-contact or indirect contact injuries, with 38 (31%) occurring without any contact during open field play. Bradley et al. [53] did not further distinguish between non-contact and indirect contact injury but reported that 22% occurred as non-contact ACL injuries. The most prevalent non-contact ACL injury situation identified from a video analysis involved a COD maneuver preceded by deceleration (70–73%) [Fig. 14, Table 7 and ESM]. Descriptions of the ACL injury mechanism suggested an abducted and flexed hip, and dynamic knee valgus with the foot externally rotated and abducted. Furthermore, Schick et al. [54] reported a heel-strike mechanism during ground contact in the majority of ACL injuries (Biomechanics of ACL injuries in American Football, ESM). Bradley et al. [53] reported that 32% of ACL injuries occurred during training, whereas Johnston et al. [10] reported that 51% of ACL injuries occurred in the preseason without further specification of whether injuries occurred in training or games. During the competitive season, ACL injuries were evenly distributed throughout the four quarters of a game and the game weeks [10]. With regard to player positions, offensive positions appeared to be exposed for a greater risk than defensive positions, particularly offensive linemen, running backs, and receivers among the offensive positions, and defensive linemen among the defensive positions [53]. There were no differences in ACL injury frequency between natural grass and artificial turf [10].

**Table 7:** Anterior cruciate ligament injuries in American Football

	Bradley et al. 2002 [53]	Johnston et al. 2018 [10]	Schick et al. 2023 [54]
Methodology	Medical staff reported	Video analysis	Video analysis
Individuals, *n* (female %)	209 (0%)	69 (0%)^a^	53 (0%)
Level of sport	Professional	Professional	Professional
Injury mechanism, *n* (%)			
Non-contact	44 (21%)	16 (23%)	22 (42%)
Indirect contact		34 (49%)	11 (21%)
Direct contact		19 (28%)	20 (37%)
Contact unspecified	148 (71%)		
Other or unknown	17 (8%)		
Situational pattern, *n* (%)			
Offensive change of direction with the ball	48 (23%)	10 (20%)	
Offensive running without the ball		4 (8%)	
Defensive running without the ball		13 (26%)	
Blocking	42 (20%)	5 (10%)	
Tackling	33 (16%)		
Defense rushing at line of scrimmage		9 (18%)	
Being blocked	36 (17%)		
Being tackled	38 (18%)	4 (8%)	
Landing from a jump		5 (10%)	
Movement pattern, *n* (%)			
Landing		6 (12%)	11 (21%)
Land and step			24 (45%)
Change of direction		30 (60%)	15 (28%)^b^
Deceleration		5 (10%)	3 (6%)
Running		5 (10%)	
Running backwards		3 (6%)	

Situational pattern overlaps within and with movement patterns, which means that the same individual can be included in both categories and the presented result may fall below or exceed 100%

*n* number of individuals

^a^Only cases with a video analysis included

^b^Includes ‘side-step’ *n* = 13 and ‘crossover’ *n* = 2

#### Australian Rules Football

**Fig. 15:**
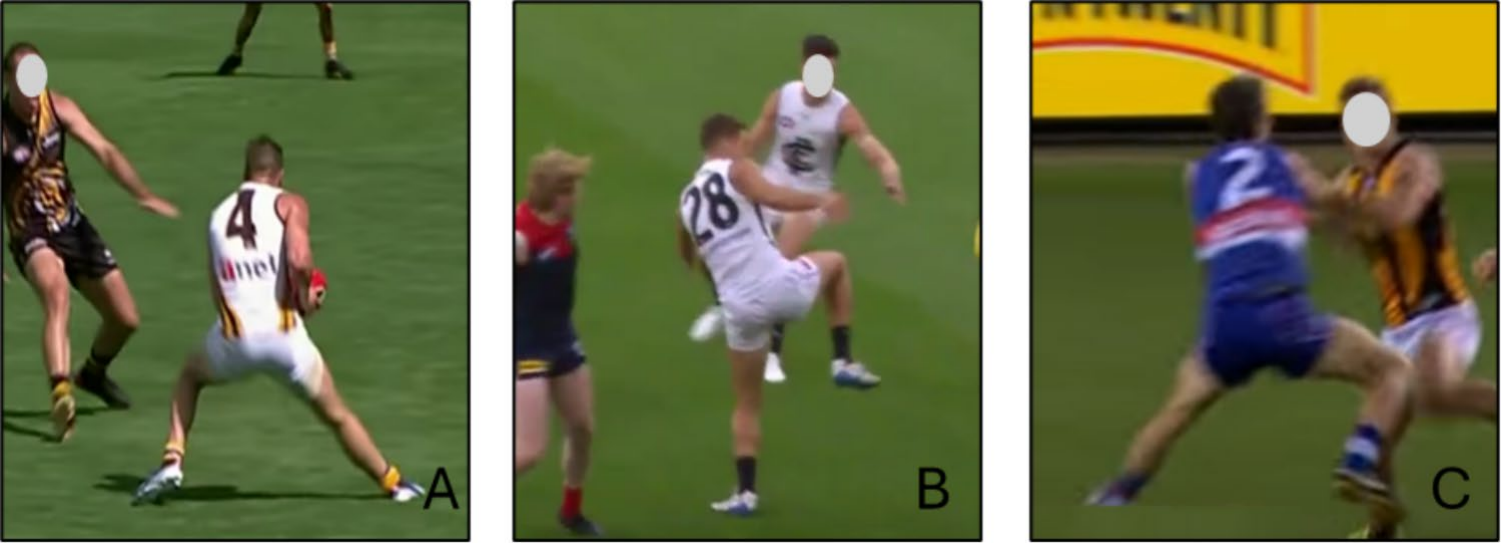
Situational patterns of anterior cruciate ligament injuries in Australian Rules football. (**A**) Offensive change of direction, (**B**) landing, and (**C**) defensive pressing. Images created by the authors from publicly available video footage (Youtube.com)

Two articles using a video analysis investigated 55 ACL injuries in professional matches of Australian Rules football [55, 56], with 21 involving female athletes [55] and 34 male athletes [56]. Cochrane et al. [56] reported a non-contact ACL injury definition for 19 ACL injuries (56%), indirect contact for 4 (12%), and direct contact for 11 (32%). Rolley et al. [55] reported a non-contact ACL injury mechanism for 13 (62%), indirect for 10 (48%), and no direct contact injuries. Rolley et al. [55] also observed that 62% of the ACL injuries were sustained during the first half of the match (quarters 1 and 2 out of four quarters). The primary movement pattern for ACL injury was related to a COD action, with situational patterns such as defensive pressing or evading the opponent (Fig. 15, Table 8, and ESM). The biomechanics of ACL injury were predominantly a knee flexion position of 0–30° followed by an excessive valgus movement with trunk rotation and lateral flexion toward the non-involved leg (ESM).

**Table 8:** Anterior cruciate ligament injuries in Australian Rules football

	Cochrane et al. 2007 [56]	Rolley et al. 2023 [55]
Methodology	Video analysis	Video analysis
Individuals, *n* (female %)	34 (0%)	21 (100%)
Level of sport	Professional	Professional
Injury mechanism, *n* (%)		
Non-contact	19 (56%)	13 (62%)
Indirect contact	4 (12%)	8 (38%)
Direct contact to injured knee	11 (32%)	
Additional details on injury mechanism, *n* (%)		
Speed of movement	Slow jog 3 (27%)^a^ Medium jog 5 (45%)^a^ Run 3 (27%)^a^	Jogging 1 (5%) Running 13 (62%) Sprinting 7 (33%)
Landing pattern		Unilateral 18 (86%) Bilateral asymmetrical 3 (14%) Bilateral symmetrical 0 (0%)
Phase of play, *n* (%)		
Offensive		7 (33%)
Defensive		14 (67%)
Situational pattern, *n* (%)		
Evading defender		4 (19%)
Defensive pressing		7 (33%)
Ground ball contest		6 (29%)
Spoiling		2 (10%)
Kicking		1 (5%)
Marking		1 (5%)
Movement pattern, *n* (%)		
Landing	9 (39%)^b^	3 (14%)
Change of direction	11 (48%)^b^	11 (52%)
Deceleration	3 (13%)^b^	

Situational pattern overlaps within and with movement patterns, which means that the same individual can be included in both categories and the presented result may fall below or exceed 100%

*n* number of individuals

^a^11 cases included for analysis

^b^23 cases included for analysis

#### Volleyball

Three articles investigated ACL injuries in volleyball and included 286 individuals, comprising 110 male individuals and 176 female individuals [29, 57, 58]. All articles reported landing or take-off during jumping actions as the major situational pattern leading to ACL injuries, with a higher prevalence during offensive attacks (spiking) in comparison to defensive actions (blocking) [57, 58] (Table 9). Ferretti et al. [57] reported that 38% of ACL injuries were sustained during training and 62% during matches. Devetag et al. [58] investigated the distribution of ACL injuries throughout the season and documented that most ACL injuries occurred during the midterm of the regular season (47% compared with preseason 26%, second round 12%, and playoffs 15%). Among player positions, spikers were identified as the most injury-prone (41–54%), followed by middle blockers (27–29%) and setters (17–19%).

**Table 9:** Anterior cruciate ligament injuries in volleyball

	Ferretti et al. 1992 [57]	Devetag et al. 2018 [58]	Takahashi et al. 2019 [29]
Methodology	Athlete reported	Athlete reported	Medical records
Individuals, *n* (female %)	52 (81%)	34 (100%)	100 (0%)
Level of sport	Professional 15 (29%) Amateur 37 (71%)	Professional	High school
Injury mechanism, *n* (%)			
Non-contact	50 (96%)	33 (97%)	M: 83 (83%) F: 90 (90%)
Indirect contact			M: 8 (8%) F: 5 (5%)
Direct contact			M: 7 (7%) F: 1 (1%)
Contact unspecified	2 (4%)	1 (3%)	M: 2 (2%) F: 4 (4%)
Situational pattern, *n* (%)			
Spiking	38 (73%)	21 (62%)	
Blocking	10 (19%)	3 (9%)	
Defensive situation, unspecified	4 (8%)		
Other landing		9 (26%)	
Movement pattern, *n* (%)			
Landing	38 (73%)	33 (97%)	M: 57 (57%)^a^ F: 73 (73%)
Jumping, take-off	7 (13%)		
Changing direction			M: 6 (6%)^a^ F: 4 (4%)
Biomechanics of injury, *n* (%)			
Valgus, external rotation valgus	22 (42%)		
Valgus, internal rotation	21 (40%)		
Unknown	9 (17%)		

Situational pattern overlaps with movement patterns, which means that the same individual can be included in both categories and the presented result may fall below or exceed 100%

*F* female, *M* male, *n* number of individuals

^a^Only non-contact injuries considered

#### Netball

Two articles investigated ACL injuries in netball and included 37 female professional netball players [59, 60]. A horizontal deceleration action, such as a bilateral landing during an offensive attack, without contact or with indirect contact from an opponent, was the most commonly reported situational pattern for an ACL injury (62–69%) (Table 10). Belcher et al. [59] reported that 38% of ACL injuries occurred during the first quarter, 19% during the second quarter, 24% during the third quarter, and 19% during the fourth quarter. Stuelcken et al. [60] reported that 38% occurred during the first quarter compared with 25% during the fourth quarter. Concerning player positions, Stuelcken et al. [60] reported that wing attackers sustained 63% of ACL injuries, centers 19%, goal shooters 13%, and wing defenders 6%. Belcher et al. [59] reported that wing attackers sustained 33% of all reported ACL injuries, followed by centers 24%, goal shooters 14%, wing defenders 14%, goal attackers 10%, and goal defenders 5%. The biomechanics of ACL injuries in netball are presented in the ESM.

**Table 10:** Anterior cruciate ligament injuries in netball

	Stuelcken et al. 2016 [60]	Belcher et al. 2022 [59]
Methodology	Video analysis	Video analysis
Individuals, *n* (female %)	16 (100%)	21 (100%)
Level of sport	Professional	Professional
Injury mechanism		
Non-contact	8 (50%)	14 (67%)
Indirect contact	8 (50%)	7 (33%)
Direct contact	0 (0%)	0 (0%)
Additional details on injury mechanism, *n* (%)		
Weightbearing one leg		4 (19%)
Weightbearing two leg		17 (81%)
Weightbearing predominantly injured leg	13 (81%)	
Speed: medium to high	13 (81%)	14 (67%)
Phase of play, *n* (%)		
Attacking	11 (69%)	16 (77%)
Defending	4 (25%)	3 (14%)
Situational pattern, *n* (%)		
Ball contest	1 (6%)	2 (9%)
Receiving pass	10 (63%)	16 (77%)
Blocking pass	3 (19%)	
Movement pattern, *n* (%)		
Single-leg landing	1 (6%)	4 (19%)
Bilateral landing		13 (62%)
Landing, specified	Split landing 6 (38%) Leap landing 5 (31%) Hop landing 1 (6%)	
Change of direction	1 (6%)	2 (10%)
Deceleration	4 (25%)	17 (81%)
Leaning too far back		11 (52%)
Leaning too far forward		1 (5%)

Situational pattern overlaps within and with movement patterns, which means that the same individual can be included in both categories and the presented result may fall below or exceed 100%

*n* number of individuals

#### Baseball

One article investigated ACL injuries in baseball [61], and included 31 male players. Data collection was conducted through a telephone survey with the injured athlete, complemented by data from medical records. The average follow-up time after ACL injury was 4.2 years, with a range from 1 to 10 years. Players were at various levels, including 3 (10%) professional, 11 (35%) collegiate, 13 (42%) high school players, youth league (*n* = 1), recreational (*n* = 2) or no reported level of play (*n* = 1). The most common playing positions to suffer an ACL injury were outfielders (*n* = 10, 32%) and infielders (*n* = 10, 32%), followed by pitchers (*n* = 9, 29%). The most common situational patterns were fielding (*n* = 21, 68%), base running (*n* = 9, 29%), and one reported ACL injury during batting (*n* = 1, 3%). For professional players, all injuries were sustained while fielding.

### Individual Sports

#### Badminton

**Fig. 16:**
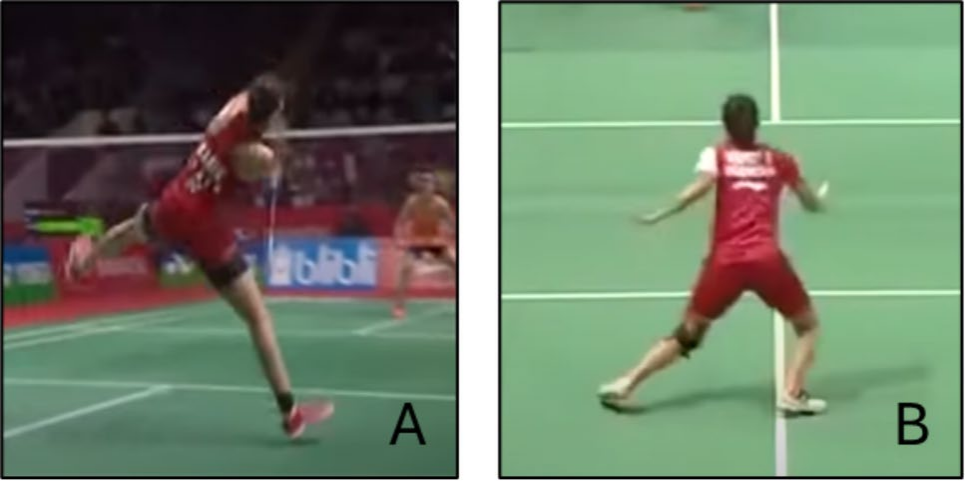
Situational patterns of anterior cruciate ligament injuries in badminton. (**A**) Landing and (**B**) change of direction. Images created by the authors from publicly available video footage (Youtube.com)

One article investigated primary ACL injuries in badminton [62]. The article included 21 badminton players of various levels, consisting of 15 female individuals and six male individuals. Information on ACL injuries was collected through athlete interviews within 1 month of the injury and supplemented by data from medical records. A total of 14 (67%) occurred during matches while seven (33%) occurred during training sessions. The mechanism of all ACL injuries was defined as non-contact with two distinct situational patterns identified. The most prevalent situation was a single-leg landing after an overhead stroke following a backward step, accounting for 48% of ACL injuries. Nine out of ten ACL injuries in this situational pattern were sustained on the knee opposite to the racket hand. The second most common situational pattern was COD, accounting for 38% of ACL injuries, where all these injuries occurred on the same knee as the racket hand during a plant or cut while moving backward or side-stepping in the forehand side of the court (Fig. 16). With regard to court location at the time of ACL injury, the rear row accounted for 71%, followed by the middle row with 24% cases, while only one injury was reported at the net. The single-leg landing ACL injury occurred predominantly in the backhand side of the court while the COD ACL injury occurred in the forehand side of the court in all reported cases. The remaining three ACL injuries (14%) lacked information about the injury situation and were categorized as others.

#### Climbing

One article investigated acute knee injuries in rock climbing and bouldering [63], and gathered injury data over a 4-year period from medical records sourced from two outpatient sports medicine clinics and complemented by a questionnaire answered by the injured climber. Among the reported injuries, 11 cases were ACL injuries, where five injuries occurred in competitive climbers and six in non-competitive climbers. The level of climbing experience differed from beginners to World Cup competitors. Ten out of 11 ACL injuries were sustained during uncontrolled falls to the ground during bouldering, while one partial ACL tear was sustained during the heel hook position.

#### Ballet and Modern Dance

Two articles investigated ACL injuries in dancers [64, 65]. Meuffels and Verhaar [64] performed a retrospective investigation with questionnaires that surveyed professional ballet dancers at an average of 5 years after an ACL injury (range 2–10 years). Liederbach et al. [65] prospectively followed 298 professional ballet and modern dancers over 5 years and documented primary ACL injuries. A total of 18 ACL injuries were recorded in the two studies, with 12 injuries occurring in female dancers and six injuries in male dancers. Seventeen out of 18 (94%) cases were non-contact situations, and one dancer suffered an ACL injury through contact during a lift by another dancer. A single-leg landing following a jump was the most common situational pattern for ACL injury, accounting for 11 (92%) cases. Specific ballet moves performed at the time of the ACL injury were ‘grand jeté’ and ‘cabriole.’ The reported biomechanics of injury was external rotation of the hip, knee valgus, external rotation of the lower leg, and pronation of the foot. Anterior cruciate ligament injuries most frequently occurred during competition (58%), while 42% were sustained at rehearsal [65]. The majority of ACL injuries (75%) were reported during the second half of a performance season [65].

#### Javelin Throwing

One article investigated a case of ACL injury in a professional female javelin thrower with a video analysis [66]. The ACL injury occurred during a competition within the initial 25% of the delivery phase, i.e., beginning with the heel-strike landing of the front leg and ending with the release of the javelin. The landing was in shallow knee flexion angle (< 10°) with a prolonged time to go through the knee flexion moment (i.e., stiff landing). From the landing, an excessive anterior tibial translation was induced on the knee joint, where the anterior edge of the tibial plateau was in front of the patella when the knee flexion angle was < 13°. The authors suggested that the increased knee valgus and internal rotation motions presented after the initial 25% of the delivery phase appeared to be the outcomes of the ACL injury, rather than being the primary cause. For further information on the ACL injury situation in javelin throwing, see ESM.

### Combat Sports

#### Judo

Three articles investigated judo, and included 1888 individuals, which consisted of 1157 male individuals, 728 female individuals, and three cases that were unspecified. Sasaki et al. [67] reported that 71% of ACL injuries occurred during training, while 29% occurred during competition. The majority of ACL injuries were the result of contact with opponents and involved either direct contact to the knee or an indirect contact mechanism. Indirect and direct contact-induced ACL injuries often occurred when a judoka was attacked by an opponent using the Kenka-yotsu grip style and executed techniques such as Osoto-gari or Kosoto-gari (Table 11).

**Table 11:** Anterior cruciate ligament injuries in judo

	Koshida et al. 2008 [68]	Takahashi et al. 2019 [29]	Sasaki et al. 2023 [67]
Methodology	Athlete reported	Medical records	Medical records
Individuals, *n* (female %)	43 (40%)	200 (50%)	1645 (37%)
Level of sport	College, high school	High school	Junior and high school
Injury mechanism, *n* (%)			
Non-contact	0 (0%)	M: 0 (0%) F: 2 (2%)	M: 271 (26%) F: 183 (30%)
Indirect contact	7 (16%)	M: 47 (47%) F: 53 (53%)	
Direct contact	36 (84%)	M: 53 (53%) F: 37 (37%)	
High-impact direct contact			M: 324 (31%) F: 166 (27%)
Low-impact direct contact			M: 439 (43%) F: 262 (43%)
Contact unspecified		F: 8 (8%)	
Situational pattern, *n* (%)			
Attempting attack	6 (14%)		M: 265 (26%) F: 163 (27%)
Being attacked	29 (67%)		M: 711 (69%) F: 423 (69%)
Counterattacked	8 (19%)		
Landing, unspecified		F: 1 (1%)^a^	
Direct contact on the involved knee after being thrown			M: 59 (6%) F: 27 (4%)
Change of direction to attack/defense		F: 1 (1%)^a^	M: 34 (3%) F: 34 (6%)
Sparring (kumite)			M: 24 (2%) F: 15 (2%)
Judo technique, *n* (%)			
Osoto-gari^b^ or Kosoto-gari^c^	8 (19%)^b^ 9 (21%)^c^		M: 439 (43%) F: 262 (43%)
Seoi-nage			M: 165 (16%) F: 104 (17%)
Tai-othoshi	6 (14%)		M: 94 (9%) F: 48 (8%)
Harai-goshi or harai-makikomi	5 (12%)		M: 106 (10%) F: 59 (9%)
Kouchi-gari	4 (9%)		M: 24 (2%) F: 15 (3%)
Ura-nage	4 (9%)		
Ne-waza			M: 31 (3%) F: 23 (4%)
Hopping backward in Ouchi-gari or Kouchi-gari			M: 24 (2%) F: 15 (3%)
Grip styles, *n* (%)			
Kenka-yotsu grip	28 (65%)		
Ai-yotsu grip	15 (35%)		

Sasaki et al. [67] divided direct contact anterior cruciate ligament injuries into low-impact contact and high-impact contact

*F* female, *M* male, *n* number of individuals

^a^Non-contact injuries considered only

^b^Major outer reap throw

^c^Minor outer reap throw

#### Wrestling

One article investigated primary ACL injuries in wrestling and included six professional athletes (unspecified sex) [69]. Video recordings were accessible for four out of the six ACL injuries. Two (33%) wrestlers competed in heavyweight, while four (67%) competed in middleweight categories. Five (83%) out of six ACL injuries occurred during competition, and one (17%) occurred during training. Five ACL injuries were sustained during the competitive season while one occurred after the season. Of the five ACL injuries suffered during competition, one (17%) occurred during the first period, three (50%) during the second period, and one (17%) during the third (last) period. Takedowns accounted for five out of six injuries, where four (67%) were sustained in a neutral position (standing facing each other) and one (17%) in an advantage position (standing above the opponent). One (17%) ACL injury occurred while the wrestler’s leg was lifted in the disadvantaged position (lying on the floor with the opponent above), where the wrestler had a flexed knee position and varus stress was applied by the opponent. In five out of six (83%) ACL injuries, the reported mechanism for ACL injury involved external tibial rotation, combined with either valgus or varus knee stress, and in one (17%) ACL injury through hyperextension.

### Ski and Board Sports

#### Skiing

**Fig. 17:**
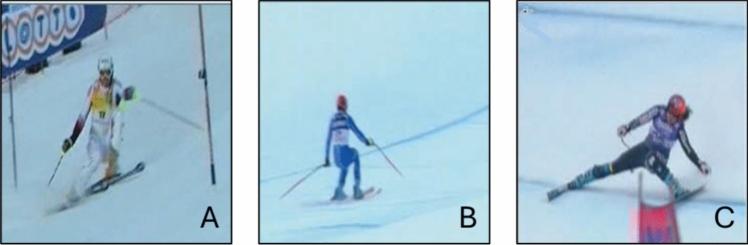
Situational patterns of anterior cruciate ligament injuries in alpine skiing. (**A**) Slip and catch, (**B**) back weighted landing, and (**C**) dynamic snowplow. Reproduced with permission from the publisher of the original article by Bere et al. [70]

Five articles investigated ACL injuries in alpine skiing [70–74], with one article covering alpine skiing and cross-country skiing [75], and two articles investigated alpine skiing when using carving skis [76, 77]. Situations resulting in ACL injuries in alpine skiing have previously been identified and categorized into five biomechanical patterns: ‘Phantom foot,’ ‘Valgus-external rotation forward fall,’ ‘Slip and Catch,’ ‘Anterior drawer,’ and the ‘Dynamic snowplow’ (Fig. 17 and ESM) [78, 79]. The most frequently reported ACL injury mechanism in recreational skiers was the ‘Valgus-external rotation’ mechanism, followed by forward fall and upper body rotation, and for professional skiers the ‘Slip and catch’ mechanism. One video analysis reported that half of the skiers had limited vision at the time of ACL injury, and 85% had no release of ski bindings [70]. Regarding snow conditions, hard and grippy snow were predominantly reported followed by either icy conditions or wet and bumpy conditions [70, 71, 75, 76]. For recreational skiers, ACL injuries most often occurred when skiing on a slope with moderate degree of difficulty (red difficulty) [76, 77]. Table 12 displays additional details on ACL injuries sustained in skiing.

**Table 12:** Anterior cruciate ligament injuries in skiing

	Fischer et al. 1994 [71]	Järvinen et al. 1994 [75]	Urabe et al. 2002 [73]	Ruedl et al. 2009 [76]	Bere et al. 2011 [70]	Ruedl et al. 2011 [77]	Bere et al. 2013 [74]	Posch et al. 2022 [72]
Methodology	Athlete reported	Athlete reported	Athlete reported	Athlete reported	Video analysis	Athlete reported	Video analysis	Athlete reported
Sport	Alpine skiing	Alpine skiing (*n* = 32), cross-country skiing (*n* = 19)	Alpine skiing	Alpine skiing (carving skis)	Alpine skiing	Alpine skiing (carving skis)	Alpine skiing	Alpine skiing
Individuals, *n* (female %)	6 (17%)	51 (76%)	80 (46%)	65 (100%)	20 (35%)	220 (73%)	2 (50%)	392 (58%)
Level of sport	Expert 50%, advanced 33%, intermediate 17%	Competitive 3 (6%), recreational 48 (94%)	Recreational	Recreational	Professional	Recreational	Professional	Recreational
Situational pattern, *n* (%)								
Turning			68 (85%)	34 (52%)	12 (60%)	T: 135 (61%) M: 41 (76%) F: 94 (64%)	2 (100%)	231 (59%)
Landing after jump				5 (8%)	4 (20%)	T: 4 (2%) M: 3 (6%) F: 1 (1%)		0 (0%)
Injury characteristics, *n* (%)								
No binding release	6 (100%)	15 (47%)	77 (96%)	53 (82%)		169 (77%)		
Ski caught an edge				26 (40%)		T: 62 (28%) M: 25 (46%) F: 37 (25%)		254 (65%)
Lost balance/ski slid away				27 (42%)		T: 105 (48%) M: 19 (35%) F: 86 (59%)		125 (32%)
Collision/avoiding collision			3 (4%)					
Traversing					2 (10%)			
Tumbling after lost control					1 (5%)			
Hooked the gate					1 (5%)			
Type of fall at injury, *n* (%)								
Forward fall with rotation				33 (51%)		T: 108 (49%) M: 30 (55%) F: 78 (54.4%)		T: 247 (63%) M: 102 (62%) F: 145 (64%)
Forward fall without rotation				4 (6%)		T: 21 (10%) M: 6 (11%) F: 15 (10%)		T: 36 (9%) M: 18 (11%) F: 18 (8%)
Backward fall with rotation				19 (29%)		T: 58 (26%) M: 14 (26%) F: 44 (31%)		T: 96 (24%) M: 39 (24%) F: 57 (25%)
Backward fall without rotation				9 (14%)		T: 12 (5%) M: 5 (9%) F: 7 (5%)		T: 13 (3%) M: 6 (4%) F: 7 (3%)

Situational pattern categories overlap, which means that the same individual can be included in two categories and the presented result can exceed 100%

*AS* alpine skiing, *CCS* cross-country skiing, *F* female, *M* male, *n* number of individuals, *T* total

#### Snowboarding

One article investigated ACL injuries in snowboarding, and included 37 individuals, which consisted of 26 male individuals and 11 female individuals [80]. Data were collected through a questionnaire answered by the ACL injured snowboarders. Among the snowboarders, 22% identified as professional riders, 68% as expert-level riders, and 10% as intermediate-level riders. Landing after a jump was the dominating situational pattern for ACL injuries reported in 35 (95%) cases, while two riders (5%) sustained the injury during a crash. The jump height leading to ACL injuries at landing was 0–10 feet (*n* = 9, 26%), 10–20 feet (*n* = 11, 31%), and > 20 feet (*n* = 10, 29%), and the remaining cases were unsure about the jumping height (*n* = 5, 14%). The ACL injury predominantly affected the front leg, observed in 34 (92%) of cases. When landing, the knee was fully extended in 6 (16%) cases, partially flexed in 12 (32%) cases, fully flexed in 2 (5%), and 17 (46%) were unsure of the knee position at the time of injury. In 20 (54%) cases, compression upon landing was reported, and 5% reported a clear sense of twisting.

#### Wakeboarding

One article investigated ACL injuries in wakeboarding, and included 52 individuals, comprising 43 male individuals and 9 female individuals [81].The wakeboarders had various levels of experience categorized as beginners (*n* = 3, 6%), intermediates (*n* = 35, 67%), and professionals (*n* = 14, 27%) [81]. A questionnaire answered by the injured athlete was used for the data collection. Forty-nine out of the 52 injured wakeboarders provided information on the specific trick they were attempting at the time of ACL injury. The most commonly reported trick associated with sustaining an ACL injury was a high jumping trick referred to as “big-air” or “double-up,” accounting for 13 out of 52 injuries (25%), the “wake-to wake” (jumping from wave to wave) was the second most prevalent trick at the time of ACL injury (21%) and the “backroll” (sideflip) and “tantrum” (backflip) were tied as the third most frequent, resulting in seven (14%) ACL injuries each. Axial loading while landing with the board against the water was the most common situational pattern for ACL (76%), 14% sustained a rotational force created by catching the edge or tip of the board against the water with a fixed foot in the binding, while the remaining cases were categorized as ‘other.’

## Discussion

This systematic review, covering 20 different sports, revealed that ACL injuries manifest specific situational patterns and diverse injury mechanisms associated with playing and movement patterns inherent in each sport (Table 13). The distribution between non-contact, indirect contact, and direct contact ACL injuries, as well as the sport-specific situational pattern during offensive or defensive actions, appears dependent on the unique playing pattern and characteristics of the specific sport. A non-contact or indirect contact ACL injury during a COD maneuver in sport-specific situational patterns was consistently among the most frequently reported injury scenarios in team ball sports (e.g., football, rugby, handball) with a range from 26 to 71%. Another distinct category of ACL injury was landing situations, predominantly reported in sports played overhead (netball, volleyball, badminton), with frequencies that ranged from 48 to 97%. Furthermore, specific situational patterns for ACL injuries involving direct contact mechanisms were identified in combat sports and physical team sports such as American Football and rugby. Additionally, unique injury scenarios related to equipment use were observed in winter sports.

**Table 13:**
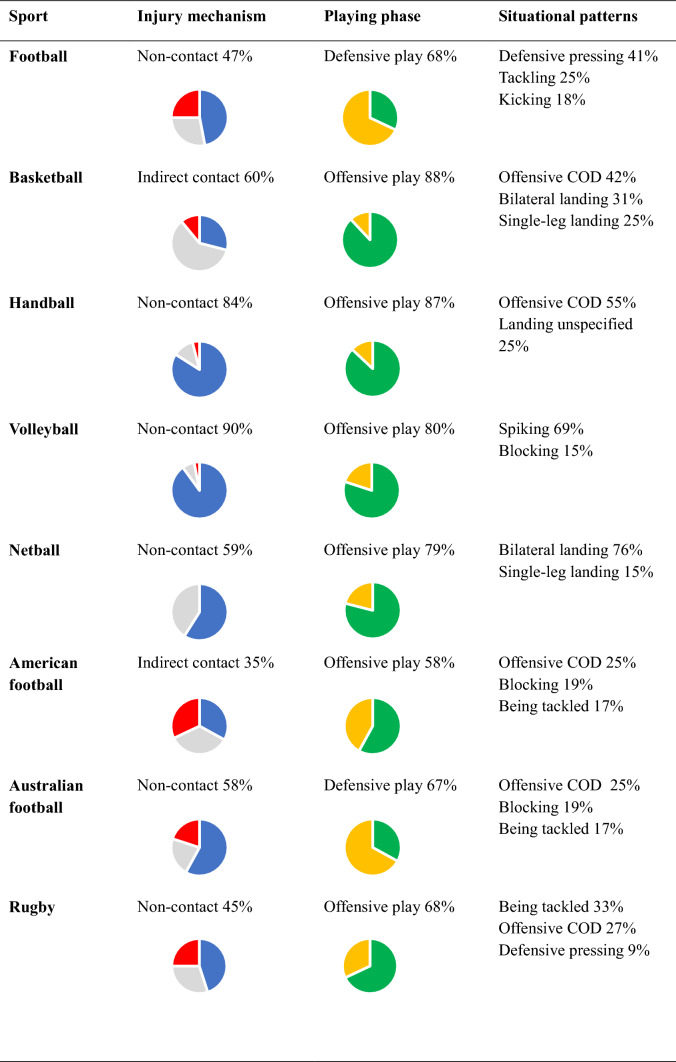
Distribution of injury mechanisms and situational patterns of anterior cruciate ligament injuries in team ball sports

*COD* change of direction

Injury mechanism: red = direct contact, gray = indirect contact, blue = non-contact. Playing phase: green = offensive play, yellow = defensive play

Enhanced understanding of the contextual factors and underlying sport-specific mechanisms is imperative to implement tailored strategies aimed at mitigating the risk of ACL injuries and to improve the efficacy of preventive strategies. Knowledge of sport-specific situations and ACL injury mechanisms also provides a foundation for re-evaluating general ACL injury prevention programs, to improve on the notion of a ‘one-size-fits-all’ approach. Consequently, we propose a categorization system for ACL injury mechanisms—COD, landing, direct contact, and gear-induced mechanisms—derived from the findings of this systematic review. This framework, summarized in Table 14, aims to facilitate the development of prevention strategies transferable across different sports and sport-specific patterns.

**Table 14:** Risk factors and recommended intervention according to categorization of ACL injury mechanisms

ACL injury category	Risk factors	Recommended interventions
Change of direction mechanism	Insufficient horizontal deceleration High-risk cutting biomechanics (e.g., limited knee and hip flexion, dynamic knee valgus) External focus of attention (e.g., on opponent or ball) Delayed or poor decision-making under time constraint	Ensure good quadriceps strength capacity and RFD to support safe deceleration strategies Train horizontal deceleration and COD technique to reduce high-risk biomechanics Use cognitive motor drills to improve decision-making under match-like conditions
Landing mechanism	Neuromuscular fatigue Stiff landing techniques Insufficient quadriceps and calf muscle strength Trunk instability and lateral trunk tilt	Strengthen ankle and calf muscles to improve shock absorption Train landing techniques and proper body positioning under fatigue, enhance core stability for upper body control Implement neuromuscular warm-ups to improve readiness and joint stability during landings
Direct contact mechanism	Tackles with direct valgus or hyperextension forces to the knee Insufficient body control in high-impact situations Improper trunk or hip positioning in combat situations	Use trunk control drills to maintain posture under contact situations, strengthen core and hip muscles to stabilize the body during collisions Refine contact technique (e.g., tackling and blocking mechanics) Consider the appropriate use of knee braces or orthoses in high-risk positions to mitigate valgus and hyperextension forces
Gear-induced mechanism	Improper landing and fall technique High-speed turns with excessive edge pressure Limited awareness of equipment function and environmental conditions	Implement neuromuscular and core training to improve balance and control Educate athletes on proper ski handling and fall techniques, and promote awareness of equipment setup (e.g., ski length, sidecut) and risk conditions Encourage routine evaluation of ski settings to align with individual skill level and terrain

*ACL* anterior cruciate ligament, *COD* change of direction, *RFD* rate of force development

### Sport-Specific Situational Patterns and Mechanisms for ACL Injury

#### COD Mechanism

The ability to perform a COD quickly is of paramount importance for performance in sports, for example, the basketball player who creates separation from an opponent before the three-point shot, the rugby player side-stepping to penetrate the defensive line during an attack, or the handball player evading the defensive player before an attempt to score. Unfortunately, these actions are also the most commonly reported situational patterns for ACL injury [10, 18, 24, 36, 48, 55]. The environment in sport is unpredictable by nature and includes highly variable, spontaneous, and unanticipated movements, i.e., the ‘chaos’ of sports. Externally directed attention to nearby opponents and the ball, as well as unexpected turns in the playing pattern can potentially increase hazardous knee-joint loads, owing to the increased complexity of the task and the time constraint imposed during sport scenarios [82]. In such sport situations, the opportunity for preparatory postural adjustments and horizontal deceleration is limited, which potentially can increase high-risk kinematics (e.g., less knee flexion, increased lateral trunk flexion, and dynamic knee valgus), thereby increasing knee-joint loading during the decelerative steps or the final foot contact at the COD maneuver [83]. However, there are clear differences in the COD ACL injury situation between sports depending on sport-specific situational patterns of the inherent sport. In football, the most common situational pattern for ACL injuries (26–40%) is defensive pressing actions when the player approaches an opponent, usually at high running speed and with external attention on the player in possession [9, 23, 34]. Interestingly, studies have reported a higher proportion of non-contact COD injuries in women’s football compared with men’s football [27, 32], which may relate to sex-specific differences in neuromuscular control and movement patterns [84–86]. In contrast, sports where the ball is carried by the hands such as rugby [18], American Football [10], Australian Rules football [55], basketball [39], and handball [48], the player who most frequently (58–87%) sustains the ACL injury is the ball carrier concentrating on the COD maneuver to get past an opponent or attempting to score. This divergency may be because of the difficulty of being in possession of the ball, where the football player requires a visual-spatial focus on keeping the ball in control, which implies a reduced speed of movement and a preparatory advantage at the time of the COD maneuver. In contrast, athletes carrying the ball in their hands can maintain a higher moving speed with a simultaneously external focus on the opponent’s next step, and thereby postpone their decision making and limit the timeframe for preparatory whole-body adjustments preceding the steps of the COD maneuver; this also applies to the football player in a defensive action [87]. In addition, COD-related ACL injuries often occur as indirect contact events. For example, in basketball, the most frequently reported situational pattern (42%) involves an offensive player attacking the rim while protecting the ball or absorbing contact from the side opposite to the knee that is subsequently injured. Contact to the contralateral side of the upper body can lead the attacking player to rotate and tilt the upper body ipsilaterally, which shifts the center of mass and results in a knee abduction moment that ultimately increases the risk of a vulnerable knee position (9).

When ACL injuries occur during a COD maneuver, the knee is usually reported in shallow angles of flexion (0–30°) in which the strain of the ACL is increased [88]. A key feature of the preceding deceleration phase of the COD maneuver is the horizontal braking force mainly induced by the quadriceps muscles in the steps before the COD maneuver (antepenultimate and penultimate foot contact). A powerful horizontal breaking maneuver allows greater knee flexion ranges of motion and lower center of mass, which can reduce the subsequent mechanical load on the knee joint and ACL strain [89]. Nevertheless, according to ‘the performance-injury conflict’ by Dos’Santos et al. [90], there is a potential conflict between the execution of a safer COD maneuver and the movement technique and biomechanics associated with faster cutting performance (e.g., greater braking force in the final foot contact at a shorter ground contact time, and lower flexion angles in the hip and knee joints). However, quadriceps strength alone is not sufficient for performing an unanticipated movement that will require the ability to react to an external stimulus, i.e., agility. In addition to muscular strength, other abilities such as a refined COD technique [91] and effective decision making [92] may enhance agility performance while concurrently reducing knee valgus loading [93]. Consequently, as a strategy to reduce COD-related ACL injuries in sports, it is recommended that coaches and clinicians ensure sufficient quadriceps strength and ability for rapid force development [94–96], work with movement analysis to improve execution of multiplanar COD tasks, [97, 98] and utilize cognitive decision-making training in the actual execution of COD maneuvers in a sport-specific context [99, 100]. Future longitudinal research is warranted to investigate how these factors, COD technique, neuromuscular capacity, and decision making, individually and collectively influence ACL injury risk over time.

#### Landing Mechanism

Injuries to the ACL sustained during landing are dominated by sports where the ball is mostly or partially played overhead in the air such as volleyball, netball, and badminton, with 57–82% of injuries suffered at landing after a jump [29, 57, 59, 60, 62]. Landing ACL injury sport situations commonly occur in offensive play when the attacking player approaches with speed and lands after perturbations or indirect contact from an opponent (netball, basketball) [43–45]. Non-contact or indirect contact during offensive play was also a typical sport situation for ACL injury reported in handball but with a lower proportion sustained during landing (14–30%) in comparison to other jumping sports such as volleyball (84%) and netball (91%). In football, landing mechanism ACL injuries commonly occurred because of a non-contact or indirect contact single-leg landing after heading the ball (3–33%).

Notably, in modern dance and ballet, most of the injuries occurred later in a performance session after several hours of dancing and later in the season, which may suggest that fatigue can be a contributing factor to the occurrence of ACL injuries in dancers [65]. Acute fatigue has been shown to alter landing biomechanics by reducing peak vertical ground reaction forces, decreasing hip and knee flexion angles, and impairing neuromuscular control during single-leg landings—factors that may increase ACL loading and injury risk [101]. However, the research is ambiguous about whether fatigue contributes to ACL injuries [102]. In netball [59], handball [46], and football [9, 19, 25] the ACL injury is reported to occur more frequently in the first half of the match play. Cumulative fatigue, caused by periods of intense training and match play with insufficient recovery, may increase the overall risk of injury and could also contribute to ACL injury risk early in a match [103]. However, we speculate that additional factors such as (1) players being rested and fueled for high-intensity actions (‘performance-injury conflict’) [104], (2) high motivation due to scores being level, (3) aggressive and risky behavior to gain respect from opponents and support from the crowd [105], and (4) inadequate neuromuscular preparation and readiness [106, 107] also play a part in the explanation of the high proportion of ACL injuries early in matches and thereby the difficulty in interpreting the significance of fatigue. This area requires further research to fully understand the interplay of these factors and their impact on ACL injury risk. Whether the situational pattern of ACL injury is different depending on the time in a match or sports competition has to our knowledge not been studied and warrants further investigation. Nevertheless, in addition to performing warm-up and neuromuscular prevention programs to improve readiness, muscular coordination, and dynamic joint stability [106, 108, 109], focus on the landing technique in a fatigue state may be advantageous for reducing landing-related ACL injuries in dancers as well as in athletes performing pivoting sports [110, 111].

Interestingly, badminton, football, and handball reported single-leg landings as more common for ACL injuries while netball reported a bilateral landing as more frequent [59, 60]. A greater proportion of landing-related ACL injuries were reported in women compared with men, particularly in bilateral landings [29, 36, 41]. While stiff landings have previously been highlighted in female athletes [93, 131, 132], recent video analyses show that female football and basketball players often demonstrate greater knee flexion angles at initial contact and injury frame compared with their male counterparts [9, 27, 41]. Instead, a more flat-footed landing with increased ankle dorsiflexion has been observed in female players, suggesting that ankle-joint stiffness and plantar flexor strength should also be considered in preventive strategies [27].

The ‘footwork rule’ in netball does not permit players to take more than one step at landing after catching the ball. This rule forces players to decelerate their body momentum with one step after landing, which causes a high ground reaction force that can increase strain on the ACL induced by the quadriceps [112], or if insufficient quadriceps strength increases the probability of the knee entering into a valgus position, with an increased risk of ACL injury [113]. Consequently, reviewing the ‘footwork rule,’ increasing quadriceps muscle strength and landing technique may be a strategy to reduce ACL injuries in netball. In handball, basketball and football ACL injuries occur during single-leg landing after perturbation or an aerial duel, and in badminton when reaching for the shuttlecock in the rear part of the court. Video analysis of the landing in football, basketball, and handball displays that players lands with lateral trunk tilt and rotation, with a heel-strike and dorsiflexed ankle-joint position at ground contact [19, 40, 44], which has been suggested to reduce the calf muscle complex contribution to absorb ground reaction forces at initial contact, resulting in greater forces being transferred to the knee [114]. Therefore, it is imperative to specify perturbation training [85], landing techniques [115, 116], and core strength [117] to stabilize upper body position during aerial duels or when overhead strikes are performed as crucial components in a comprehensive neuromuscular prevention program.

#### Direct Contact Mechanism

In multiple sports, direct contact ACL injuries are often considered unfortunate accidents and difficult to prevent. However, in sports such as American Football, Australian Rules football, and rugby, approximately one third of ACL injuries occur as a direct trauma to the knee. This underscores the imperative need to address this specific ACL injury mechanism. Notably, offensive linemen in American Football face a higher likelihood of sustaining ACL injuries because of their blocking positioning [10], which exposes them to direct contact mechanism situations from both opponents and teammates, including inadvertent impacts to the knee. Similarly, in rugby, a common situation leading to ACL injuries involves direct contact during breakdown situations such as tackles and ruck formations [52]. While the traditional practice of ‘rucking,’ where players used their feet to contest possession, has been outlawed in rugby union for many years because of player safety concerns, the term is still sometimes used to describe contested phases following a tackle. Most direct-contact ACL injuries typically occur when a player is tackled, although injuries can also occur, albeit less frequently, in players executing the tackle. Mitigating these direct-contact ACL injuries in open-field sports demands a continuous reassessment of game regulations. For instance, the National Football League’s implementation of the “Chop-Block rule change” serves as an example in American Football. The Chop-Block rule change deemed the maneuver in which an offensive player blocks an opponent around the thigh while another offensive player engages the same opponent above the waist as illegal [118]. In addition, there are disparities in the usage of prophylactic braces among different sports. American Football linemen frequently use braces made of metal and plastic, while Australian Rules football and rugby only permit soft braces. Whether braces are effective in preventing ACL injury remains a subject of debate, often based on the difficulty of counteracting the rotational mechanism implicated in non-contact ACL injuries [119]. However, stable braces may potentially reduce the effects of direct contact forces causing valgus/varus or hyperextension, offering a means to diminish injury risks associated with such forces [120].

In combat sports, such as wrestling and judo, direct contact ACL injuries are a significant part of the ACL injury panorama [68, 69]. Typically, these injuries occur when the foot remains planted while the knee receives a direct lateral or anterior force, leading to valgus collapse often coupled with rotational forces or, in certain cases, hyperextension of the knee. In judo, the authors [29, 67, 68] suggested that factors affecting ACL injury prevention are primarily related to the trunk and hip positioning imbalance and improper throwing technique rather than the sport-specific situation itself. Specifically, fixed internal rotation of the hip joint and ipsilateral trunk flexion have been identified as injury mechanisms underpinning ACL injury [18, 40]. Mitigating these factors can reduce the valgus moment on the knee joint, thereby preventing knee injuries. Therefore, implementing systematic neuromuscular training that targets core muscles and emphasizes proper hip positioning techniques is warranted for combat sports athletes to reduce the risk of contact ACL injury and optimize postoperative rehabilitation strategies [121].

#### Gear-Induced ACL Injuries

In the event of a fall or accident, external objects such as skis or boards attached to the feet can extend the external moment arm, generating excessive rotational forces at the knee joint, which can result in an ACL rupture [122]. In this category, there is an obvious difference between board and skis, in terms of stance (bilateral or unilateral) and body position (riding with side or front first), which also form the ACL injury mechanism landscape. Approximately six ACL injuries per 100 competitive skiers per season are reported [123]. In snowboarders, the reported risk of ACL injuries is lower [124] and almost every case occurs through compression forces against the knee of the front leg when landing after a jump, with the center of mass too far behind the front foot. This forces the quadriceps to quickly perform an eccentric action to keep the body upright and counteract the ground contact forces, which ultimately leads to a rapid and excessive anterior tibial translation that likely causes the ACL injury [125]. A similar situational pattern has also been described in alpine skiing during a back-weight ‘tail landing’ or a backward fall (ESM) but is less common than the biomechanical patterns of the ‘valgus-external rotation’ and the ‘slip and catch’ when executing a turn. The biomechanics of the ‘slip and catch’ implies a skier out of balance backwards and/or inwards and in the next turn cutting the inside edge of the ski and generating a considerable valgus/rotational moment to the knee. This ACL injury situation arises within 60 ms when skiing at high speeds and is predominantly reported in professional skiers [70], while for recreational skiers the riding speed has not been able to be determined; however a relationship between the ‘slip and catch’ mechanism and a higher risk-taking profile has been identified [72].

To reduce ACL injuries in skiing, knowledge of ski geometry should account for the balance between the development of the fastest possible ski and a safe ski with the least possible risk of injury. Longer and wider skis contribute to more stability and shock absorption, which improve the possibility of higher speeds. The self-steering effect of carving skis (hourglass shaped) facilitates turning more aggressively at a higher speed, which increases the force against the edge on the inside of the ski and thereby the forces upon the knee joint [126]. The wide ski phenomenon is believed to have contributed to the fact that the amount of ‘slip and catch’ injuries (ESM) has increased among competitive skiers, while the ‘phantom-foot’ mechanism was reported more frequently when longer and more straight skis were used and seems to be on the decline with the introduction of carving skis [72]. The regulations for racing skis were updated in 2012 by the International Ski Federation in an attempt to reduce the increased rate of severe knee injuries in professional alpine skiing. Ski length and sidecut radius were increased to make the skis less aggressive, but whether this effect has been protective against ACL injuries has not been established [127]. With regard to recreational skiers, there is a lack of knowledge about ski geometry [128], which may negatively contribute to the risk of ACL injury. Last, as restricted vision and changing snow conditions also have been identified as a potential contributor to ACL injuries in skiing [70], avoiding skiing in a snowfall may be an important environmental aspect as well [129]. To summarize, gear-induced ACL injuries are multifactorial and as there probably is no single solution that will eliminate ACL injuries, preventive training strategies need to be established and promoted in addition to the continuous development of safe ski equipment and improvements in ski settings and usage. Recommendations for these preventive training strategies include: (1) active neuromuscular training, which includes motor control exercises, lower limb strength symmetry, and core strength training [78, 130, 131] and (2) understanding and awareness to recognize risk situations when skiing and act upon these with proper fall technique [132].

### Limitations

There was considerable heterogeneity in the scientific literature on the ACL injury mechanism regarding the study design and methodologies used for data collection: 49% of the included studies used a video analysis, 40% used athlete reported-questionnaires or interviews, and 11% used medical staff reports or data from medical records. Articles based on reports from medical staff or medical records have limitations in detailing ACL injury mechanisms and circumstances. The information derived from medical records or application documents in many cases proved inadequate to provide a nuanced understanding of the ACL injury mechanism, with the absence of a biomechanical analysis as an additional limitation. The athlete-reported data collection emphasizes the athlete’s subjective experience and interpretation of a rapid and sudden injury situation, crucial for understanding the ACL injury mechanism. In addition, the wide range of follow-up times in athlete-reported methods (from 2 days to 4 years post-ACL injury) poses an evident risk of recall bias. However, Olsen et al. [48] conducted a comparison of ACL injury mechanism video analysis and athlete questionnaires, and reported strong agreements for movement pattern and injury mechanism. To assess the quality of articles with athlete- or medical staff-reported ACL injury situations, the JBI checklist (20) was chosen because of the absence of an ideal quality appraisal tool. Unfortunately, the reliability and validity of the JBI checklist have not been evaluated, and moreover, two items were omitted from our quality assessment as they were deemed redundant. Nevertheless, the structure and content of the JBI checklist were deemed the most appropriate for the study objectives and utilized as a framework to evaluate the quality of the included articles.

If video analysis articles were considered only, articles on alpine skiing, wrestling, javelin throwing, and seven different team-sports would be included. While a video analysis offers more precise insights into the situational patterns of ACL injuries, there is variability among these articles in terms of systematic approaches, descriptions of injury mechanisms and biomechanics, as well as factors such as the number of camera angles and video quality. A difference was observed in the interpretation of the QA-SIVAS scale, where 18 articles in this systematic review overlapped with the validation study for the tool [20]. In our study, six articles were rated as high quality compared with one in the validation study, five versus seven as good quality, four versus eight as moderate quality, and three versus two as low quality, highlighting discrepancies in the interpretation of individual studies. Additionally, several video analysis articles note the difficulty of verifying the exact moment of ACL injury when conducting biomechanical analyses. Consequently, the reported joint angles and body motions may not represent the actual mechanisms causing the ACL injury, but rather be a result of the injury [39].

Furthermore, a limitation of this systematic review is that all included articles were published in English only, potentially introducing a language bias and excluding relevant research published in other languages.

In summary, the incorporation of several different methodologies to examine ACL injury mechanisms may restrict the internal validity of our results. Moreover, other considerable factors such as the age of athletes, level of sport, and timing of the injury should be observed when comparing ACL injury mechanisms and situational patterns for the various included sports. Additionally, the included studies encompassed athletes with vastly different performance levels that ranged from young recreational athletes to professionals, which may influence sport-specific movement patterns and limit the generalizability of overarching conclusions. Approximately one third of the included studies investigated both female and male athletes, but few presented results of differences between sexes for comparable groups (e.g., sport, playing level), which limits opportunities to provide conclusions based on sex differences.

### Future Research

To further improve our understanding of ACL injuries and to develop targeted ACL injury prevention and sport-specific rehabilitation programs, future research is warranted on (1) potential sex-based differences in ACL injury mechanisms, (2) potential differences between primary and secondary ACL injury mechanisms, and (3) potential differences in ACL injury mechanisms during the spectrum of a match and timepoints of season.

## Conclusions

The nature of ACL injuries varies significantly between sports based on the injury definition, with a range from non-contact to indirect and direct contact injuries in different situational patterns such as COD or landing, as well as specific athletic actions related to the playing or movement pattern of the sport. To effectively mitigate the risk of ACL injury, a thorough understanding of the underlying situations and mechanisms within various sports is imperative. Increased understanding may allow for the implementation of tailored strategies in each sport to reduce the likelihood of ACL injuries. As a proposed approach, we suggest categorizing ACL injury mechanisms into distinct groups: COD mechanism, landing mechanism, direct contact mechanism, and gear-induced mechanism. This categorization aims to facilitate intervention strategies that can be adapted across different sports, irrespective of variations in the playing and movement pattern specific to each sport, optimizing their applicability across diverse sports contexts.

## Supplementary Information

Below is the link to the electronic supplementary material.

Supplementary file1 (DOCX 60 KB)

Supplementary file2 (DOCX 70 KB)

Supplementary file3 (DOCX 25672 KB)

## Declarations

### Availability of data and material

The data that support the findings of this study are available from the corresponding author upon reasonable request.
