# Supplementary material for: Sport-Specific Injury Mechanisms and Situational Patterns of ACL Injuries: A Comprehensive Systematic Review
Source: Sports Med. 2025 Jul 21;55(10):2489–527. doi: 10.1007/s40279-025-02271-w (PMC12513954; doi:10.1007/s40279-025-02271-w)
Supplement: Supplementary file 1 — Supplementary file1 (DOCX 60 KB) [file 40279_2025_2271_MOESM1_ESM.docx]

**Supplemental file 1**

**Search strategy**

The initial search strategy in December 2022.

**Database:** Medline (Ovid), *MEDLINE(R)* *ALL* *(OvidSP)*

**Date:** 2022-12-07

**No** **of** **results:** 1363 ref

| **#** | **Searches** | **Results** |
| --- | --- | --- |
| 1 | exp Anterior Cruciate Ligament Injuries/ | 12502 |
| 2 | Anterior Cruciate Ligament/ | 12035 |
| 3 | (injuries or injury or injured or tear or tears or rupture or ruptures or ruptured).ab,kf,ti. | 1128321 |
| 4 | 2 and 3 | 5508 |
| 5 | ((ACL or (anterior adj5 cruciate adj5 ligament*)) adj4 (injuries or injury or injured or tear or tears or rupture or ruptures or ruptured)).ab,kf,ti. | 10195 |
| 6 | 1 or 4 or 5 | 17254 |
| 7 | (injur* adj3 (pattern* or cause* or situation*)).ab,kf,ti. | 50395 |
| 8 | (situational pattern* or mechanism* or biomechanical analysis or injury video* or video analysis).ab,kf,ti. | 2628760 |
| 9 | 7 or 8 | 2668886 |
| 10 | 6 and 9 | 1618 |
| 11 | limit 10 to english language | 1487 |
| 12 | (comment or editorial).pt. | 1419801 |
| 13 | 11 not 12 | 1470 |
| 14 | (animals not (animals and humans)).sh. | 5038960 |
| 15 | (animal or animals or rat or rats or mouse or mice or rodent or rodents or dog or dogs or cat or cats or hamster or hamsters or rabbit or rabbits or swine or murine or porcine or horses or horse).ti. | 2073890 |
| 16 | 14 or 15 | 5436105 |
| **17** | **13** **not** **16** | **1363** |

**Database:** Embase (Ovid), *1974* *to* *2022* *December* *06*

**Date:** 2022-12-07

**No** **of** **results:** 1907 ref

| **#** | **Searches** | **Results** |
| --- | --- | --- |
| 1 | exp anterior cruciate ligament injury/ | 12580 |
| 2 | anterior cruciate ligament/ | 13289 |
| 3 | (injuries or injury or injured or tear or tears or rupture or ruptures or ruptured).ab,kf,ti. | 1434139 |
| 4 | 2 and 3 | 6119 |
| 5 | ((ACL or (anterior adj5 cruciate adj5 ligament*)) adj4 (injuries or injury or injured or tear or tears or rupture or ruptures or ruptured)).ab,kf,ti. | 12674 |
| 6 | 1 or 4 or 5 | 19801 |
| 7 | (injur* adj3 (pattern* or cause* or situation*)).ab,kf,ti. | 64407 |
| 8 | (situational pattern* or mechanism* or biomechanical analysis or injury video* or video analysis).ab,kf,ti. | 3157950 |
| 9 | 7 or 8 | 3209144 |
| 10 | 6 and 9 | 2192 |
| 11 | limit 10 to english language | 2022 |
| 12 | limit 11 to (article or article in press or conference abstract or conference paper or erratum or letter or note or "preprint (unpublished, non-peer reviewed)" or "review" or short survey) | 2007 |
| 13 | (animal not (animal and human)).sh. | 1169578 |
| 14 | (animal or animals or rat or rats or mouse or mice or rodent or rodents or dog or dogs or cat or cats or hamster or hamsters or rabbit or rabbits or swine or murine or porcine or horses or horse).ti. | 2216438 |
| 15 | 13 or 14 | 3113636 |
| **16** | **12** **not** **15** | **1907** |

**Database:** The Cochrane Library (Wiley)

**Date:** 2022-12-07

**No of results: 103**

*Trials:* *103*

*Editorials:* *0*

*Special* *collections:* *0*

*Clinical* *answers:* *0*

| **ID** | **Search** | **Hits** |
| --- | --- | --- |
| #1 | MeSH descriptor: [Anterior Cruciate Ligament Injuries] explode all trees | 937 |
| #2 | MeSH descriptor: [Anterior Cruciate Ligament] explode all trees | 688 |
| #3 | (injuries OR injury OR injured OR tear OR tears OR rupture OR ruptures OR ruptured):ti,ab,kw (Word variations have been searched) | 83095 |
| #4 | #2 AND #3 | 548 |
| #5 | (((ACL OR (anterior NEAR/4 cruciate NEAR/4 ligament*)) NEAR/3 (injuries OR injury OR injured OR tear OR tears OR rupture OR ruptures OR ruptured))):ti,ab,kw (Word variations have been searched) | 1906 |
| #6 | #1 OR #4 OR #5 | 1949 |
| #7 | (injur* NEAR/2 (pattern* OR cause* OR situation*)):ti,ab,kw (Word variations have been searched) | 1186 |
| #8 | (situational NEXT pattern* OR mechanism* OR "biomechanical analysis" OR injury NEXT video* OR "video analysis"):ti,ab,kw (Word variations have been searched) | 65585 |
| #9 | #7 OR #8 | 66633 |
| **#10** | **#6** **AND** **#9** **in** **Cochrane** **Reviews,** **Cochrane** **Protocols,** **Trials,** **Clinical** **Answers,** **Special** **Collections** | **103** |

**Database:** Amed (Ebsco)

**Date:** 2022-12-08

| **No** **of** **results:** 204 ref**#** | **Query** | **Results** |
| --- | --- | --- |
| **S11** | **S6** **AND** **S9**  **Expanders** **-** **Apply** **related** **words;** **Apply** **equivalent** **subjects** **Narrow** **by** **Language:** **-** **english** | **204** |
| S10 | S6 AND S9 | 206 |
| S9 | S7 OR S8 | 16,307 |
| S8 | TI ( situational pattern* OR mechanism* OR biomechanical analysis OR injury video* OR video analysis ) OR AB ( situational pattern* OR mechanism* OR biomechanical analysis OR injury video* OR video  analysis ) OR KW ( situational pattern* OR mechanism* OR biomechanical analysis OR injury video* OR video analysis ) | 15,691 |
| S7 | TI ( (injur* N2 (pattern* OR cause* OR situation*) ) OR AB ( (injur* N2 (pattern* OR cause* OR situation*) ) OR KW ( (injur* N2 (pattern* OR cause* OR situation*) ) | 765 |
| S6 | S1 OR S4 OR S5 | 1,495 |
| S5 | TI ( ((ACL OR (anterior N4 cruciate N4 ligament*)) N3 (injuries OR injury OR injured OR tear OR tears OR rupture OR ruptures OR ruptured)) ) OR AB ( ((ACL OR (anterior N4 cruciate N4 ligament*)) N3 (injuries OR injury OR injured OR tear OR tears OR rupture OR ruptures OR ruptured)) ) OR KW ( ((ACL OR (anterior N4 cruciate N4 ligament*)) N3 (injuries OR injury OR injured OR tear OR tears OR rupture OR  ruptures OR ruptured)) ) | 984 |
| S4 | S2 AND S3 | 1,210 |
| S3 | TI ( injuries OR injury OR injured OR tear OR tears OR rupture OR ruptures OR ruptured ) OR AB ( injuries OR injury OR injured OR tear OR tears OR rupture OR ruptures OR ruptured ) OR KW ( injuries  OR injury OR injured OR tear OR tears OR rupture OR ruptures OR ruptured ) | 36,008 |
| S2 | (ZU "anterior cruciate ligament") | 1,671 |
| S1 | (ZU "anterior cruciate ligament injuries") | 11 |

**Database:** Pedro

**Date:** 2022-12-09

**No** **of** **results:** 49 references in total, out of which 15 are duplicates. There are 34 unique references.

**Comment:** Pedro has limitations combining search terms. Search was conducted in “Simple search”. 18 separate searches have been conducted to cover the search.

| **#** | **Search** **terms** | **Resultat** |
| --- | --- | --- |
| **#** | anterior cruciate ligament injur* pattern* | **10** |
| **#** | anterior cruciate ligament injur* situation* | **2** |
| **#** | anterior cruciate ligament injur* cause* | **10** |
| **#** | anterior cruciate ligament injur* mechanism* | **7** |
| **#** | anterior cruciate ligament injur* biomechanical analysis | **7** |
| **#** | anterior cruciate ligament injur* video analysis | **0** |
| **#** | anterior cruciate ligament tear* pattern* | **2** |
| **#** | anterior cruciate ligament tear* situation* | **0** |
| **#** | anterior crucate ligament tear* cause* | **2** |
| **#** | anterior cruciate ligament tear* mechanism* | **3** |
| **#** | anterior cruciate ligament tear* biomechanical analysis | **2** |
| **#** | anterior cruciate ligament tear* video analysis | **0** |
| **#** | anterior cruciate ligament rupture* pattern* | **0** |
| **#** | anterior cruciate ligament rupture* situation* | **1** |
| **#** | anterior cruciate ligament rupture* cause* | **3** |
| **#** | anterior cruciate ligament rupture* mechanism* | **0** |
| **#** | anterior cruciate ligament rupture* biomechanical analysis | **0** |
| **#** | anterior cruciate ligament rupture* video analysis | **0** |

**Supplemental file 2**

The updated search in October 2023 used the same search strategy with the following results.

Medline

(n =132)

Amed

(n =7)

Embase

(n =188)

Cochrane Library

(n =7)

Pedro
(n = 14)

Total
(n =348)

New articles
(n =251)

Duplicates
(n =97)

Articles included (n=6)
