# Supplementary material for: Sport-Specific Injury Mechanisms and Situational Patterns of ACL Injuries: A Comprehensive Systematic Review
Source: Sports Med. 2025 Jul 21;55(10):2489–527. doi: 10.1007/s40279-025-02271-w (PMC12513954; doi:10.1007/s40279-025-02271-w)
Supplement: Supplementary file 2 — Supplementary file2 (DOCX 70 KB) [file 40279_2025_2271_MOESM2_ESM.docx]

**Supplemental file 3**

**Biomechanics**

Biomechanics of ACL injuries in football based on video analysis articles.

|  | **Brophy, 2014 [24]** | **Waldén, 2015 [20]** | **Della Villa, 2020 [9]** | **Grassi, 2020 [27]** | **Lucarno, 2021 [28]** | **De Carli, 2022 [31]** | **Rekik, 2023 [35]** |
| --- | --- | --- | --- | --- | --- | --- | --- |
| **Biomechanics of injury** |  |  |  |  |  |  |  |
| *Foot strike heel, n (%)* |  | 11 (33%) | 51 (48%) |  | 8 (32%) |  |  |
| *Foot strike flat, n (%)* |  | 7 (21%) | 30 (28%) |  | 12 (48%) |  |  |
| *Foot strike toe, n (%)* |  | 9 (27%) | 15 (14%) |  | 2 (8%) |  |  |
| *Ankle flexion, n (%)* |  |  |  |  |  |  |  |
| *Ankle rotation – External, n (%)* |  | 6 (18%) | 68 (66%) |  | 17 (68%) |  | 14 (93%) |
| *Ankle rotation – Internal, n (%)* |  | 8 (24%) | 6 (6%) |  | 1 (4%) |  |  |
| *Ankle rotation – Neutral, n (%)* |  | 11 (33%) | 10 (10%) |  | 1 (4%) |  |  |
| *Knee flexion 0-30°, n (%)* | M: 25 (78%) F: 16 (70%) |  |  |  |  |  | 13 (87%) |
| *Knee flexion 30-60°, n (%)* | M: 6 (19%) F: 5 (22%) |  |  |  |  |  | 1 (7%) |
| *Knee flexion 60-90°, n (%)* | M: 0 (0%) F: 1 (4%) |  |  |  |  | 2 (2%) ** |  |
| *Knee valgus, n (%)* | M: 16 (50%) F: 10 (43%) |  | 28 (27%) |  | 13 (52%) | 80 (63%) *** |  |
| *Knee varus, n (%)* | M: 1 (3%) F: 0 (0%) |  | 2 (2%) |  | 5 (20%) | 2 (2%) **** |  |
| *Knee neutral, n (%)* | M: 14 (44%) F: 12 (52%) |  | 66 (63%) |  | 7 (28%) |  |  |
| *Knee hyperextension, n (%)* |  |  |  |  |  | 6 (5%) |  |
| *Knee pivot shift, n (%)* |  |  |  |  |  | 20 (16%) |  |
| *Knee position unknown, n (%)* |  |  |  |  |  | 11 (9%) | 1 (7%) |
| *Hip flexion, n (%)* | M: 28 (88%) F: 20 (87%) |  |  |  |  |  | 14 (93%) |
| *Hip abduction, n (%)* | M: 24 (75%) F: 16 (70%) |  | 74 (72%) |  | 18 (72%) |  |  |
| *Hip adduction, n (%)* | M: 2 (6%) F: 0 (0%) |  | 8 (8%) |  | 0 (0%) |  |  |
| *Hip neutral, n (%)* | M: 5 (16%) F: 6 (26%) |  | 15 (15) |  | 1 (4%) |  |  |
| *Trunk toward injured limb, n (%)* |  |  |  | 2 (8%) |  |  |  |
| *Trunk toward uninjured limb, n (%)* |  |  |  | 12 (48%) |  |  |  |
| *Trunk neutral, n (%)* |  |  |  | 9 (36%) |  |  |  |

**Supplemental file 4**

Biomechanics of ACL injuries in basketball based on video analysis articles.

|  | **Krosshaug, 2007 [44]** | **Koga, 2010 [42] and 2018 [43]** | **Gill, 2023 [46]** | **Petway, 2023 [47]** |
| --- | --- | --- | --- | --- |
| **Biomechanics of injury** |  |  |  |  |
| *Ankle sagittal IC, n (%)* |  |  | Flat 14 (37%)  Toe 8 (21%)  Heel 7 (18%)  Unclear 9 (24%) |  |
| *Ankle eversion IC, n (%)* |  |  | Eversion 1 (3%)  Inversion 2 (5%)  Neutral 23 (60%)  Unclear 12 (32%) |  |
| *IF 66ms, n (%)* |  |  | Eversion 1 (3%)  Inversion 1 (3%)  Neutral 20 (52%)  Unclear 16 (42%) |  |
| *Foot rotation IC, n (%)* |  |  | External 9 (24%)  Internal 2 (5%)  Neutral 11 (29%)  Unclear 16 (42%) |  |
| *IF 66ms, n (%)* |  |  | External 6 (16%)  Internal 3 (8%)  Neutral 10 (26%)  Unclear 19 (50%) |  |
| *Knee sagittal IC* |  |  | 12.6 ± 8.8 |  |
| *IF 66ms* |  |  | 44.3 ± 26.0 |  |
| *Knee frontal IC* |  |  | 5.1 ± 6.1 |  |
| *IF 66ms* |  |  | 19.1 ± 13.9 |  |
| *Hip sagittal IC* |  |  | 39.3 ± 25.2 |  |
| *IF 66ms* |  |  | 50.8 ± 23.6 |  |
| *Hip abduction IC, n (%)* |  |  | Abduction 30 (79%)  Adduction 1 (3%)  Neutral 1 (3%)  Unclear 6 (15%) |  |
| *IF 66ms, n (%)* |  |  | Abduction 26 (68%)  Adduction 1 (3%)  Neutral 1 (3%)  Unclear 10 (26%) |  |
| *Trunk rotation IC, n (%)* |  |  | Towards injured 13 (34%)  Towards uninjured 8 (21%)  Neutral 11 (29%)  Unclear 6 (16%) |  |
| *IF 66ms, n (%)* |  |  | Towards injured 13 (34%)  Towards uninjured 8 (21%)  Neutral 7 (19%)  Unclear 10 (26%) |  |
| *Trunk tilt IC, n (%)* |  |  | Towards injured 23 (60%)  Towards uninjured 4 (11%)  Neutral 6 (16%)  Unclear 5 (13%) |  |
| *IF 66ms, n (%)* |  |  | Towards injured 25 (66%)  Towards uninjured 2 (5%)  Neutral 4 (11%)  Unclear 7 (18%) |  |
| *Dorsiflexion at bilateral landing (°) IC* |  |  |  | 18 ± 15 |
| *Dorsiflexion at single leg casting (°) IC* |  |  |  | 19 ± 14 |
| *Knee valgus, n (%)* |  |  |  |  |
| *Knee valgus at single leg landing (°)* |  |  |  |  |
| *IC* | M: 3 ± 1  (0-5)  F: 4 ± 1  (0-5) | 0 **** |  |  |
| *IF* | M: 6 ± 3  (2-10) F: 8 ± 0 (8-9) ****** | 12 **** |  |  |
| *Knee valgus at bilateral landing (°)* |  |  |  |  |
| *IC* | M: 2 ± 1  (1-4) F: 5 ± 2  (3-8) |  |  |  |
| *IF* | M: 3 ± 2  (1-7) F: 7 ± 3  (4-14) ****** |  |  |  |
| *Knee valgus at change of direction (°)* |  |  |  |  |
| *IC* | M: 2 ± 2  (1-4) F: 6 ± 0 (5-6) | 1.5 ± 2 ***** |  |  |
| *IF* | M: -2 ± 12  (-11-7)  F: 10 ± 3  (9-12) ****** | 11.5 ± 2 *****  (40 ms) |  |  |
| *Knee flexion at single leg landing (°)* |  |  |  |  |
| *IC* | M: 8 ± 6 (3-16) F: 10 ± 4 (5-14) | 19 **** |  | 34 ± 8 |
| *IF* | M: 18 ± 6 (10-28) F: 18 ± 4 (13-23)  ****** | 38 **** (40 ms) |  |  |
| *Knee flexion at bilateral landing (°)* |  |  |  | 21 ± 15 |
| *IC* | M: 9 ± 7 (3-19)  F: 15 ± 4 (10-22) |  |  |  |
| *IF* | M: 17 ± 6 (11-23) F: 27 ± 7 (18-40) ****** |  |  |  |
| *Knee flexion at change of direction (°)* |  |  |  | 16 ± 8 ***** |
| *IC* | M: 12 ± 2 (11-13) F: 14 ± 11 (7-22) | 29 ± 2 ***** |  |  |
| *IF* | M: 23 ± 7 (18-28) F: 27 ± 4 (24-29) ****** | 51 ± 4 ***** (40 ms) |  |  |
| *Hip flexion at single leg landing (°)* |  |  |  |  |
| *IC* | M: 16 ± 8 (5-27) F: 20 ± 10 (5-27) | 42 **** |  |  |
| *IF* | M: 22 ± 7 (17-31) F: 20 ± 7 (14-30) ****** | 44 **** |  |  |
| *Hip flexion at bilateral landing (°)* |  |  |  |  |
| *IC* | M: 18 ± 4 (13-22) F: 25 ± 8 (17-44) |  |  |  |
| *IF* | M: 18 ± 5 (14-26)  F: 32 ± 11 (21-54) ****** |  |  |  |
| *Trunk extension at bilateral landing (°)*  *IC* |  |  |  | -6.9 ± -11.4 |
| *Landing from COM at bilateral landing (°)*  *IC* |  |  |  | 53 ± 8 |
| *Hip flexion at change of direction (°)* |  |  |  |  |
| *IC* | M: 29 ± 6 (25-33) F: 37 ± 7 (32-42) | 72 ± 20 ***** |  |  |
| *IF* | M: 22 ± 1 (21-22) F: 45 ± 6 (41-49) ****** | 76 ± 23 *****  (40ms) |  |  |
| *Hip abduction at single leg landing (°)* |  |  |  |  |
| *IC* | M: 12 ± 5 (6-20) F: 15 ± 21 (1-46) | 24 **** |  |  |
| *IF* | M: 12 ± 3 (9-16) F: 15 ± 22 (-1-48) ****** | 21 ****  (40ms) |  |  |
| *Hip abduction at bilateral landing (°)* |  |  |  |  |
| *IC* | M: 19 ± 13 (9-38) F: 15 ± 9 (6-33) |  |  |  |
| *IF* | M: 17 ± 16 (6-41) F: 14 ± 10 (5-36) ****** |  |  |  |
| *Hip abduction at change of direction (°)* |  |  |  |  |
| *IC* | M: 11 ± 20  (-3-25) F: 19 ± 6  (14-23) | 33 ± 3 ***** |  |  |
| *IF* | M: 8 ± 21  (-7-23) F: 14 ± 6  (10-19) ****** | 25 ± 5 *****  (40ms) |  |  |
| *Contralateral hip abduction (swing leg) at single leg landing (°)*  *IC* |  |  |  | 105 ± 17 |
| *Trunk lateral flexion at single leg landing (°)*  *IC* |  |  |  | 22 ± 5 |
| *Trunk lateral flexion at single leg casting (°)*  *IC* |  |  |  | 18 ± 8 |

*SD=Standard deviations; IC=Initial contact; IF=Injury frame; IF 66ms=Injury frame estimated 66ms after initial contact;.
**=1 case
***= 2 cases
**** = Injury frame was predefined as 50 or 33 ms after initial contact
***** = During single-leg casting*

**Supplemental file 5**

Biomechanics of ACL injuries in handball based on video analysis articles.

|  | **Ebstrup, 2000 [45]** | **Olsen, 2004 [51]** | **Koga, 2010 [42] and 2018 [43]** |
| --- | --- | --- | --- |
| **Biomechanics of injury** |  |  |  |
| *Forceful valgus close to extension with tibial rotation, n (%)* |  | 19 (95%) |  |
| *Valgus with internal rotation, n (%)* | 1 (50%) |  |  |
| *Varus with external rotation, n (%)* | 1 (50%) |  |  |
| *Foot strike heel, n (%)* |  |  | 7 (100%) |
| *Knee valgus at single leg landing (°)* |  |  | * |
| *IC* |  |  | 2 ± 0 |
| *IF* |  | 13 ± 3 | 12 ± 4 |
| *Knee valgus at change of direction (°)* |  |  | ** |
| *IC* |  |  | 0 ± 2 |
| *IF* |  | 14 ± 4 | 11 ± 3 |
| *Knee flexion at single leg landing (°)* |  |  | * |
| *IC* |  |  | 26 ± 2 |
| *IF* |  | 19 ± 5 | 58 ± 4 |
| *Knee flexion at change of direction (°)* |  |  | ** |
| *IC* |  |  | 20 ± 8 |
| *IF* |  | 13 ± 5 | 43 ± 14 |
| *Hip flexion at single leg landing (°)* |  |  | * |
| *IC* |  |  | 54 ± 7 |
| *IF* |  |  | 58 ± 4 |
| *Hip flexion at change of direction (°)* |  |  | ** |
| *IC* |  |  | 45 ± 18 |
| *IF* |  |  | 45 ± 14 |
| *Hip abduction at single leg landing (°)* |  |  | * |
| *IC* |  |  | 4 ± 13 |
| *IF* |  |  | -2.5 ± 16 |
| *Hip abduction at change of direction (°)* |  |  | ** |
| *IC* |  |  | 23 ± 10 |
| *IF* |  |  | 17 ± 12 |

*MS=Milliseconds; IC=Initial contact; IF=Injury frame (40 ms after IC).
*=2 cases
**= 7 cases*

**Supplemental file 6**

Biomechanics of ACL injuries in rugby based on video analysis articles.

|  | **Montgomery, 2018 [55]** | **Della Villa, 2021 [19]** |
| --- | --- | --- |
| **Biomechanics of injury  (non-contact and indirect contact)** |  |  |
| *Foot strike at:* | *** | ** |
| *IC, n (%)* | Heel 10 (67%)  Flat 3 (20%) Toe 2 (13%) | Heel 18 (51%) Flat 15 (43%) Toe 2 (6%) |
| *IF, n (%)* |  | Heel 0 (0%) Flat 34 (100%) Toe 0 (0%) |
| *Ankle flexion at:*  *IC, median with maximum and minimum values* |  | * -10° (10 to -40°) |
| *IF, median with maximum and minimum values* |  | -10° (15 to -50°) |
| *Ankle rotation at:* | *** | ** |
| *IC, n (%)* | External 11 (73%)  Neutral 1 (7%) Internal 1 (7%) Unsure 1 (7%) | External 15 (49%) Neutral 11 (35%) Internal 5 (16%) |
| *IF, n (%)* |  | External 17 (53%) Neutral 6 (19%) Internal 9 (28%) |
| *Knee flexion at:*  *IC, median with maximum and minimum values* |  | * 15° (50 to 0°) |
| *IF, median with maximum and minimum values* |  | 30° (65 to -20° |
| *Knee valgus/varus at:* |  | ** |
| *IC, n (%)* |  | Valgus 13 (37%) Neutral 22 (63%) Varus 0 (0%) |
| *IF, n (%)* |  | Valgus 33 (94%) Neutral 1 (3%) Varus 1 (3%) |
| *Hip flexion at:* |  | * |
| *IC, median with maximum and minimum values* |  | 40° (65 to 20°) |
| *IF, median with maximum and minimum values* |  | 37.5° (70 to 5°) |
| *Hip abduction/adduction at:* |  | ** |
| *IC, n (%)* |  | Abduction 32 (91%) Neutral 3 (9%) Adduction (0%) |
| *IF, n (%)* |  | Abduction 30 (86%) Neutral 5 (14%) Adduction 0 (0%) |
| *Trunk flexion at:* |  | * |
| *IC, median with maximum and minimum values* |  | 15° (50 to -25°) |
| *IF, median with maximum and minimum values* |  | 10° (95 to -20°) |
| *Trunk lateralflexion at:* |  | ** |
| *IC, median with maximum and minimum values  n (%)* |  | 5° (30 to -15°)  Toward injured leg 20 (67%) Neutral 6 (20%) Toward uninjured leg 4 (13%) |
| *IF, median with maximum and minimum values*  *n (%)* |  | 10° (25 to -20°)  Toward injured leg 24 (80%) Neutral 3 (10%) Toward uninjured leg 3 (10%) |
| *Trunk rotation at:* |  | ** |
| *IC, n (%)* |  | Toward injured leg 8 (22%) Neutral 14 (39%) Toward uninjured leg 14 (39%) |
| *IF, n (%)* |  | Toward injured leg 3 (8%) Neutral 5 (14%) Toward uninjured leg 28 (78%) |
| **Biomechanics of injury (direct contact)** |  |  |
| *External force + valgus loading, n (%)* | 7 (19%) | 9 (16%) |
| *Hyperextension, n (%)* |  | 4 (7%) |
| *Posterior tibial force application, n (%)* |  | 3 (5%) |

*Positive values indicate flexion and negative values indicate extension.* *N=Number of individuals, IC = Initial contact, IF= Injury frame.
*= Only non-contact and indirect contact injuries considered, 28 cases included
**= Only non-contact and indirect contact injuries considered, 35 cases included
***= Only non-contact injuries considered, 15 cases included*

**Supplemental file 7**

Biomechanics of ACL injuries in American football based on video analysis articles.

|  | **Johnston, 2018  [11]** | **Schick, 2023 [57]** |
| --- | --- | --- |
|  | Video analysis | Video analysis |
| **Biomechanics of injury** |  |  |
| *Foot strike, n (%)* |  | Heel: 32 (60%) Forefoot: 21 (40%) |
| *Ankle position (frontal plane), n (%)* | Abducted: 45 (90%)  Neutral: 3 (6%)  Adducted: 2 (4%) |  |
| *Ankle position (sagittal plane), n (%)* |  | Plantarflexion: 31 (58%) 0-30°: 28 (52%) 30-60°: 3 (6%)  Dorsiflexion: 22 (42%) 0-30°: 21 (40%) 60-90°: 1 (2%) |
| *Ankle rotation (coronal plane), n (%)* | External: 45 (90%)  Neutral: 5 (10%)  Internal: 0 (0%) |  |
| *Knee position (sagittal plane), n (%)* | 0-45°: 41 (82%)  45-90°: 8 (16%)  >90°: 1 (2%) | 0-30°: 33 (62%) 30-60°: 9 (17%) 60-90°: 9 (17%) 90-120°: 2 (4%) |
| *Knee position (frontal plane), n (%)* | Neutral: 2 (4%)  Abducted: 46 (92%)  Adducted: 2 (4%) | Neutral: 20 (38%) Valgus: 28 (53%) Varus: 5 (9%) |
| *Knee position (coronal plane), n (%)* |  | Neutral: 26 (49%) Internal: 12 (23%) External: 15 (28%) |
| *Hip position (sagittal plane), n (%)* | Extension: 0 (0%)  Neutral: 2 (4%)  Flexion: 48 (96%) | 0-30°: 26 (49%) 30-60°: 19 (36%) 60-90°: 8 (15%) |
| *Hip position (frontal plane), n (%)* | Abducted: 43 (86%)  Neutral: 5 (10%)  Adducted: 2 (4%) |  |

**Supplemental file 8**

Biomechanics of ACL injuries in Australian rules football based on video analysis articles.

|  | **Cochrane, 2007 [59]** | **Rolley, 2023 [58]** |
| --- | --- | --- |
| **Biomechanics of injury** |  |  |
| *Knee position, n (%)* | Valgus 9 (47%) ** Internal rotation 8 (42%) ** Varus 2 (11%) ** External rotation 1 (5%) ** | Valgus 17 (94%)*** |
| *Foot strike, n (%)* |  | Rearfoot 14 (74%)  Midfoot 3 (14%)  Forefoot 2 (9%) Unable to determine 2 (9%) |
| *Degree of knee flexion, n (%)* | ≤30° 15 (65%) ** ≥30° 1 (4%) ** Unknown 7 (30%) ** |  |
| *Trunk position sagittal, n (%)* |  | Flexed trunk 11 (52%) Neutral trunk 9 (43%) Trunk hyperextended 1 (5%) |
| *Trunk position frontal, n (%)* |  | Lateral flexion toward the uninjured leg  9 (45%) Lateral flexion toward the injured leg 8 (38%) Neutral 3 (14%) Unable to determine 1 (5%) |
| *Trunk position coronal, n (%)* |  | Rotation toward the uninjured leg 14 (70%) Rotation toward the injured leg 4 (19%) Neutral 2 (9%) Unable to determine 1 (5%) |

**** =*23 cases included for analysis*
*** =*18 cases included for analysis*

**Supplemental file 9**

Biomechanics of ACL injuries in netball based on video analysis articles.

|  | **Stuelcken, 2016 [63]** | **Belcher, 2022 [62]** |
| --- | --- | --- |
| **Biomechanics of injury** |  |  |
| *Knee close to extension, n (%)* | 19 (90%) | 2 (13%) |
| *Valgus collapse at: Initial contact, n (%) Injury frame, n (%)* | 2 (10%) 7 (33%) | 12 (75%) |
| *Hip internal rotation, n (%)* |  | 7 (43%) |
| *Foot flat, n (%)* | 21 (100%) |  |
