# Supplementary material for: Sport-Specific Injury Mechanisms and Situational Patterns of ACL Injuries: A Comprehensive Systematic Review
Source: Sports Med. 2025 Jul 21;55(10):2489–527. doi: 10.1007/s40279-025-02271-w (PMC12513954; doi:10.1007/s40279-025-02271-w)
Supplement: Supplementary file 3 — Supplementary file3 (DOCX 25672 KB) [file 40279_2025_2271_MOESM3_ESM.docx]

**Supplemental file 10**

**Bibliography of situational patterns of ACL injuries in sports**

**Football**

***Defensive pressing***

1.
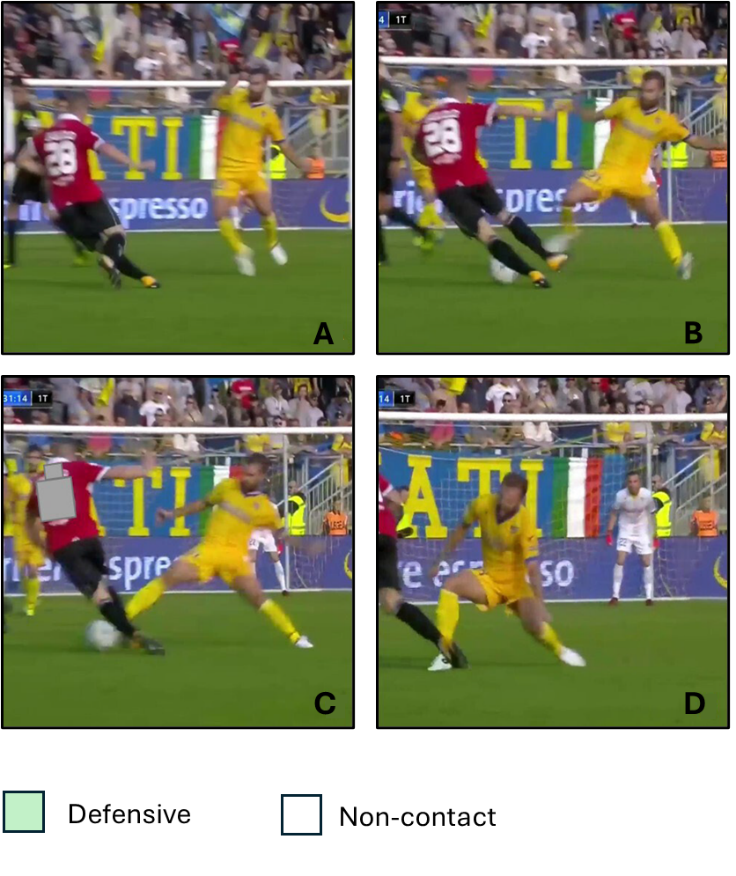
Opponent in possession of the ball. Defending player (yellow jersey) approaching the opponent at high speed.
2. Initial contact, right foot. The player makes a rapid deceleration and a sidestep cut to reach the ball or to tackle the opponent, but no player contact.
3. Injury frame, left knee ACL injury. Wide foot plant with ipsilateral trunk lean to maintain balance, rotating the trunk towards his right leg and placing the entire load on his left leg.
4. Left hip and knee are in abducted positions and the ankle joint is in eversion.

*
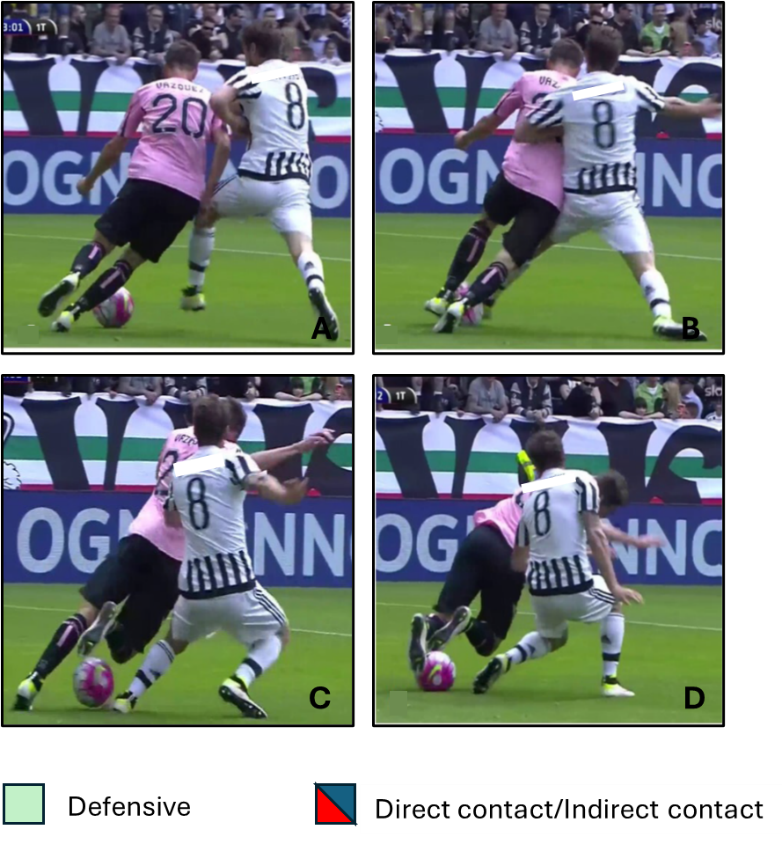
****Tackling***

1. Defensive player (white and black jersey) approaching the opponent from the side to make a tackle.
2. Initial contact with the left foot while tackling. Wide foot plant and contact to left side at the waist.
3. Injury frame, left knee ACL injury. Ipsilateral trunk lean, abducted hip, shallow knee flexion and planted foot in eversion.

D. Loss of balance after injury.

*
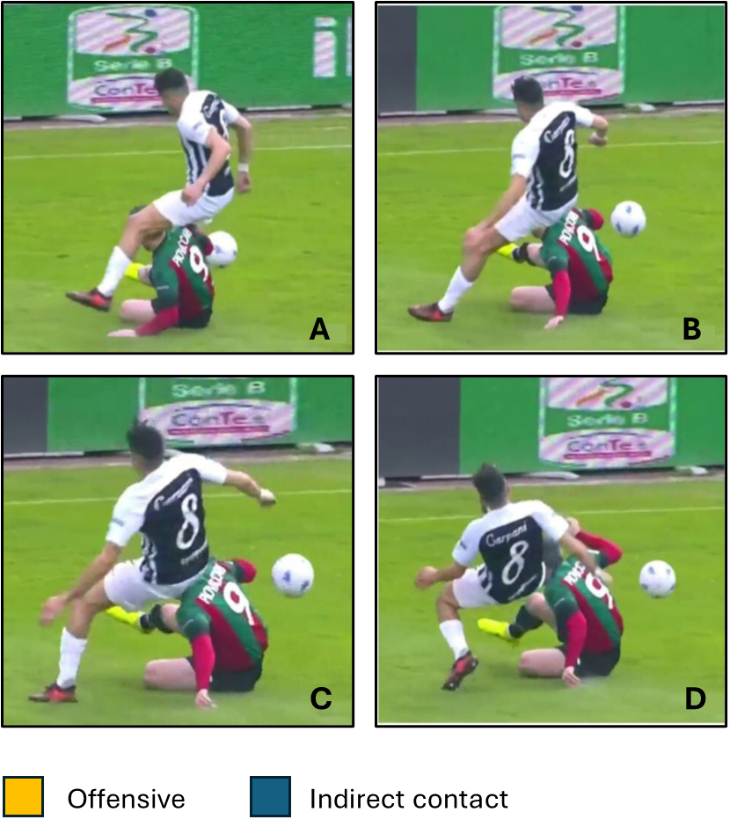
****Being tackled***

1. Medium-speed running chasing the ball. The opponent tackles from the right side to reach ball from the right leg.
2. Initial contact, heel landing left leg and mechanical perturbational due to tackle at right leg.
3. Injury frame, left knee ACL injury. The player is back weighted with upper body rotated to the right, hip in abduction and planted foot in external rotation.
4. Loss of balance after injury.

***Regain balance after kicking***


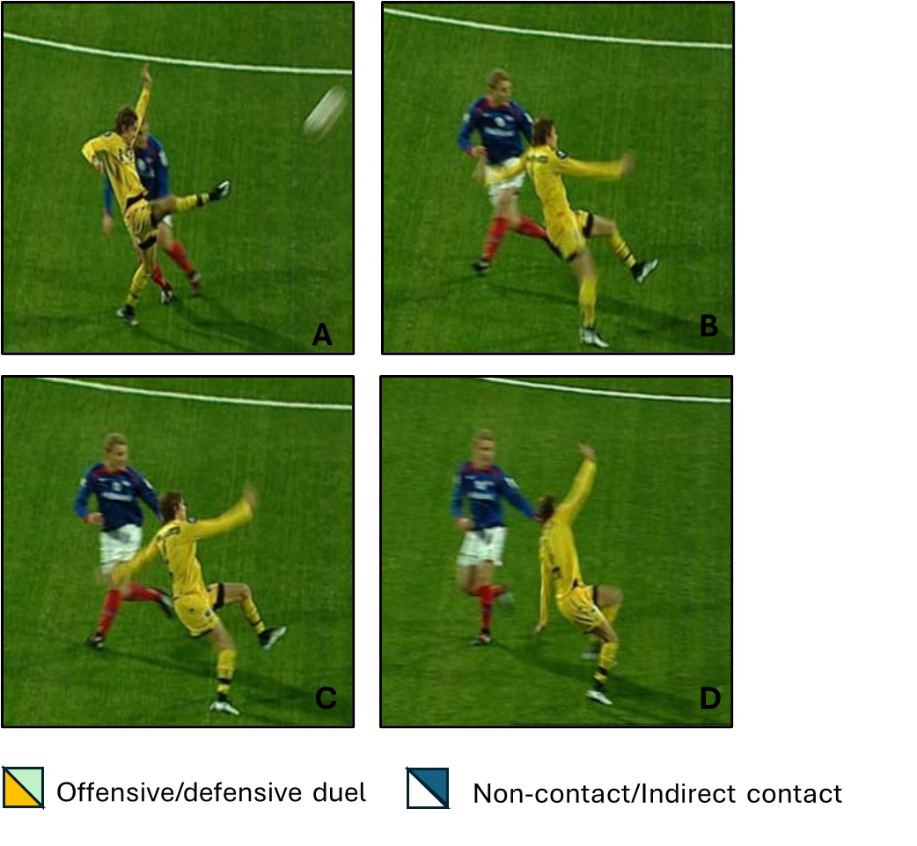
A. Player clearing the ball with the right foot.

B. Initial contact, right foot. Strikes the pitch with the forefoot of right leg and rotates the trunk to the left.

C. Injury frame, right knee ACL injury. The player is out of balance backwards and puts the entire load on his right leg.

D. The knee is abducted, and the foot is in eversion (dynamic valgus).

All images are reproduced with permission from the publishers of the original articles by Della Villa et al. [9] and Waldén et al. [20]

**Basketball**

*
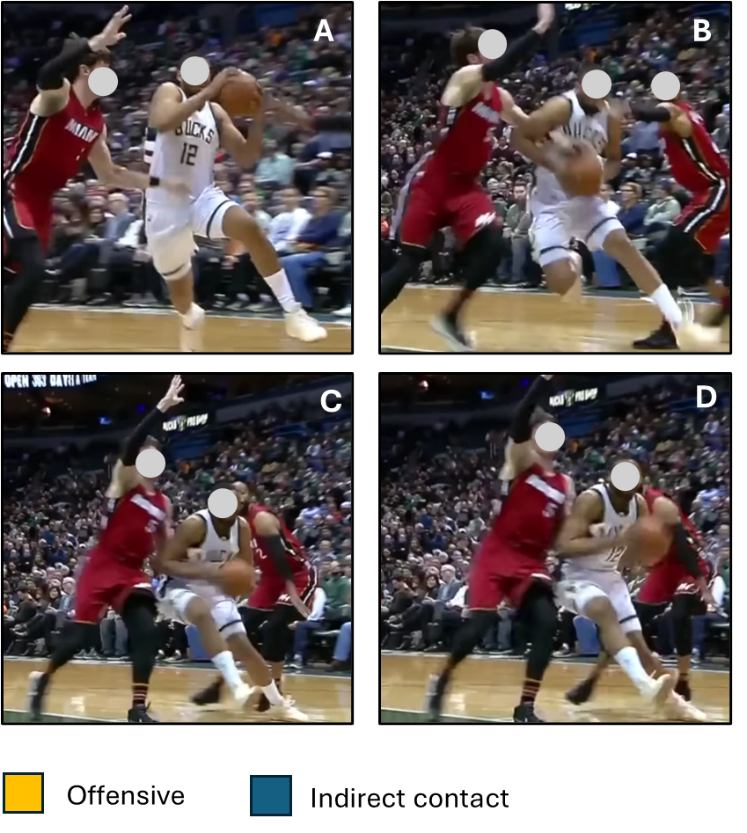
****Attacking the basket***

A. The offensive player drives to the basket and protects the ball from the defensive player on his right side.

B. Initial contact, left leg heel strike and wide foot plant with upper body mechanichal perturbation to the right side from the defensive player.

C. Injury frame, left knee ACL injury. Ipsilateral trunk lean, left hip in flexion and abduction, foot planted in external rotation and eversion.

D. Dynamic knee valgus.

*
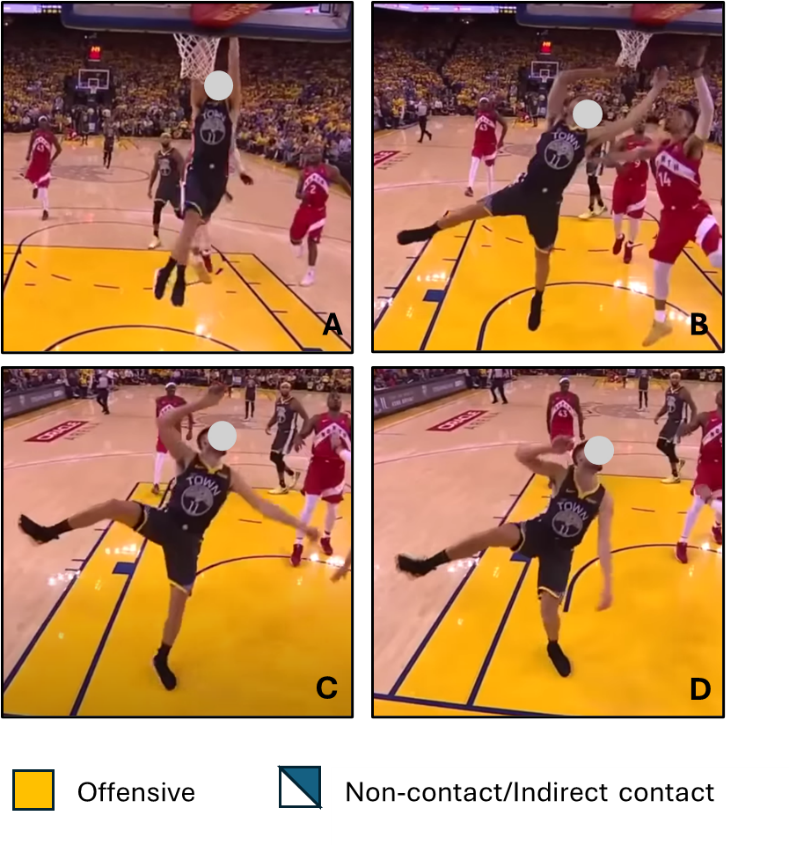
****Landing***

1. The offensive player dunks.
2. Mechanical perturbation from a defensive player from the left side of the upper body while airborne, right leg swings in abduction to counterbalance.
3. Injury frame, left knee ACL injury. The player is out of balance, single-leg landing with ipsilateral trunk lean and externally rotated foot with axial compression upon ground reaction force.

D. Hip internal rotation and dynamic knee valgus.

*
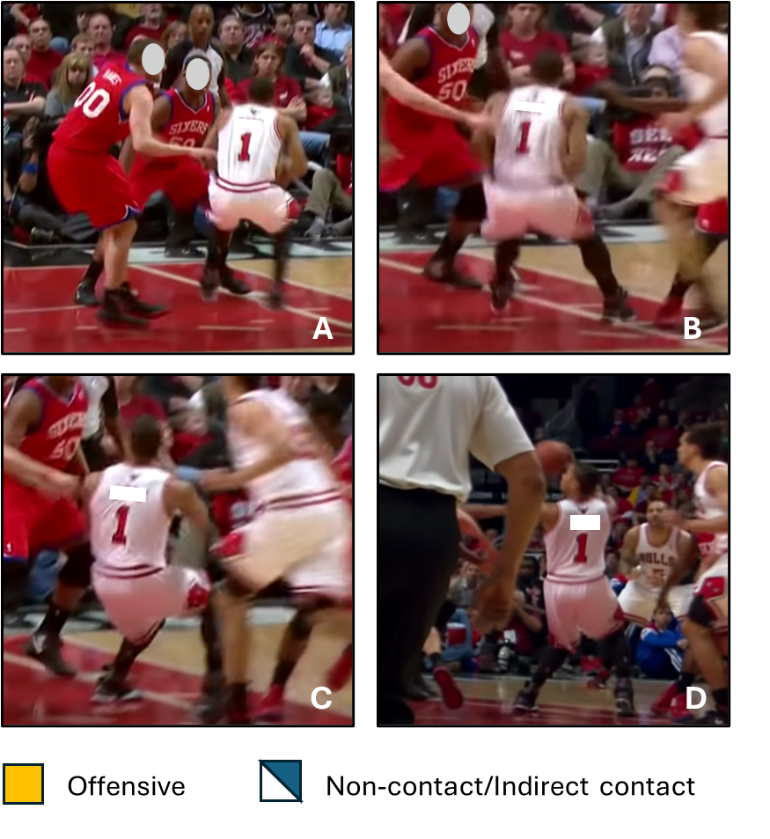
****Jump stop***

1. The offensive player performs a bilateral jump stop (bilateral landing) to create separation from the opponent. No contact from the opponent.
2. Initial contact, left leg with a wide foot plant and heel strike ahead of the athlete’s center of mass coupled with trunk lean.
3. Injury frame, left knee ACL injury. Ipsilateral trunk lean and flexion, left hip in flexion and abduction, foot planted in external rotation and eversion
4. Dynamic knee valgus

***Offensive change of direction***

1.
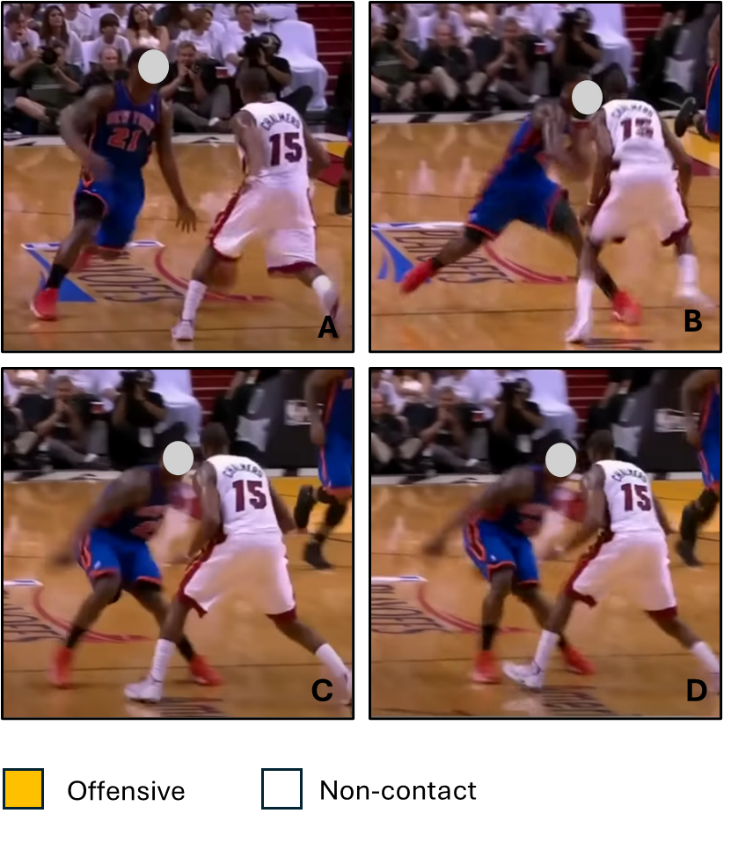
The offensive player performs a cutting maneuver in the build-up play. No contact from the opponent.
2. Initial contact, left leg. The player change direction with a wide foot plant and heel strike ahead of the athlete’s center of mass coupled with trunk lean.
3. Injury frame, left knee ACL injury. Ipsilateral trunk lean and flexion, left hip in flexion and abduction, foot planted in external rotation and eversion
4. Dynamic knee valgus.

All images created by the authors from publicly available video footage (Youtube.com)

**Handball**

*
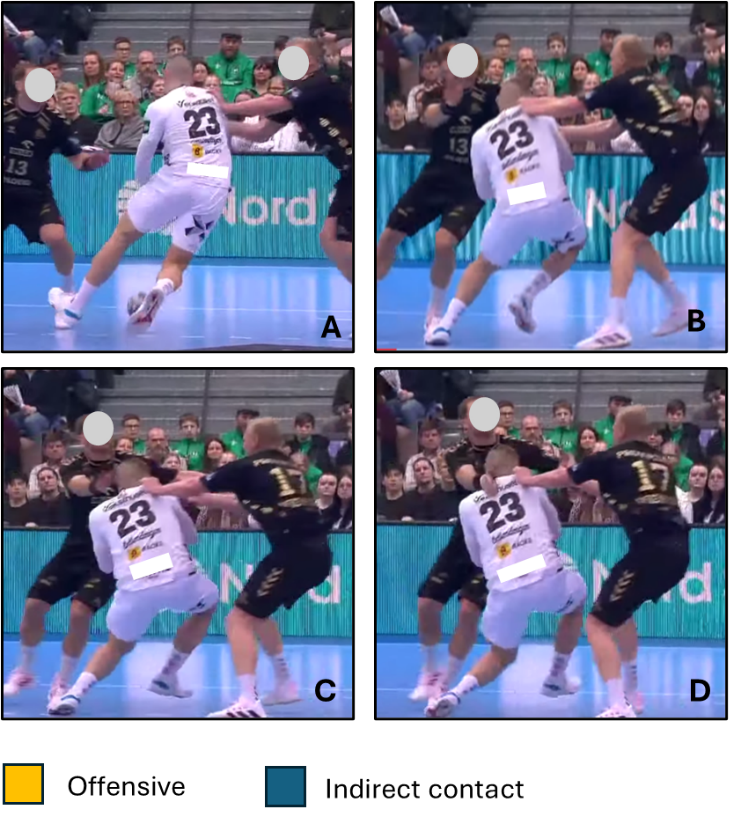
****Offensive breakthrough***

1. The offensive player attempts an offensive breakthrough.
2. Initial contact left foot. Mechanical perturbation to the right side of the upper body when the defensive player pushing the upper body into lateral flexion.
3. Injury frame, left knee ACL injury. Ipsilateral trunk lean, hip abduction and foot planted in external rotation and eversion.
4. Dynamic knee valgus.

***Step cut maneuver***

1.
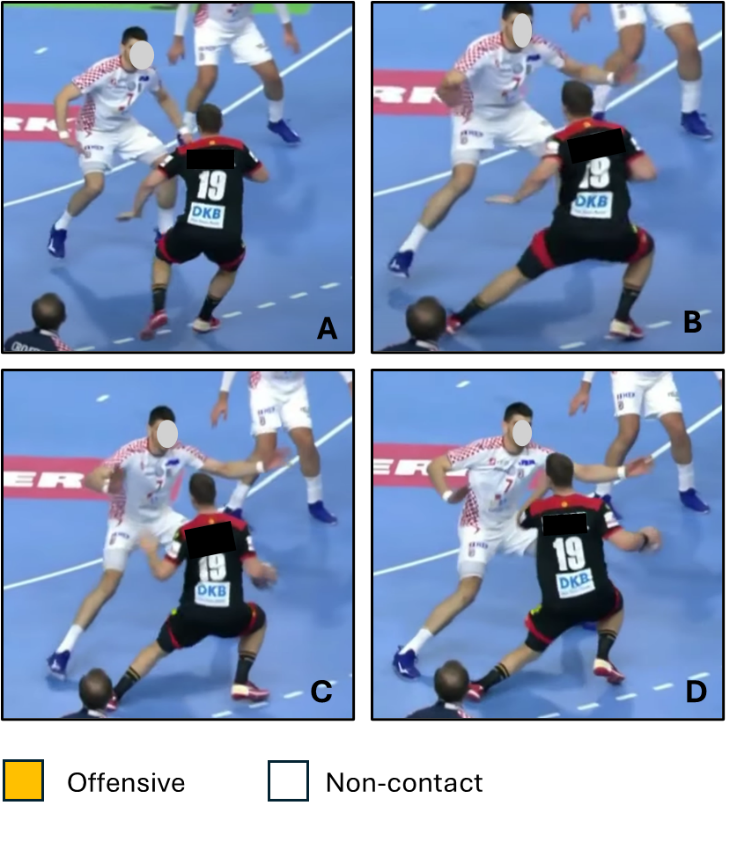
The offensive player pushing off from the right leg to the left leg, to perform a step-cut maneuver. No contact to the defensive player.
2. Initial contact, left foot. Hip abduction (wide foot plant) and extended knee position with upper body moment towards the left.
3. Injury frame, left knee ACL injury. Hip abduction, foot planted in external rotation and eversion.
4. Dynamic knee valgus.

*
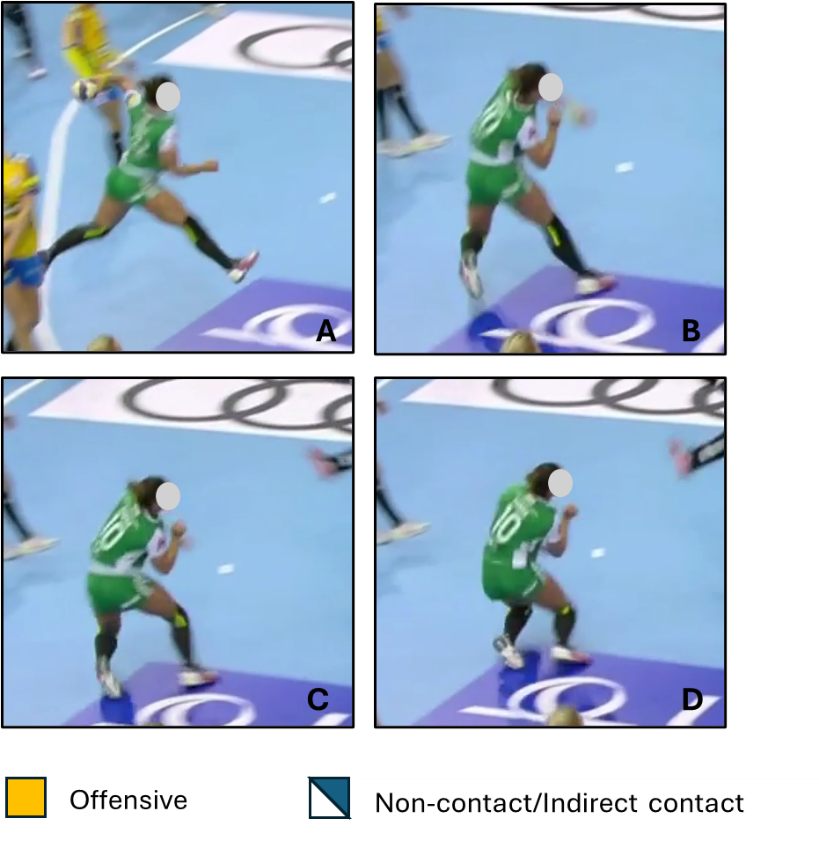
****Landing***

1. The offensive player finishes an attack with a jump shot with left hand. No contact from the opponent.
2. Initial contact, right foot strikes the ground in front of the athlete’s center of mass. Horizontal speed is high, coupled with axial compression at landing.
3. Injury frame, right knee ACL injury. The upper body continues to travel to the right side to counterbalance the upper body moment of the left-handed throw. Ipsilateral trunk lean, hip abduction and foot plant in front of center of mass.
4. Loss of balance after injury.

All images created by the authors from publicly available video footage (Youtube.com)

**American football**

***
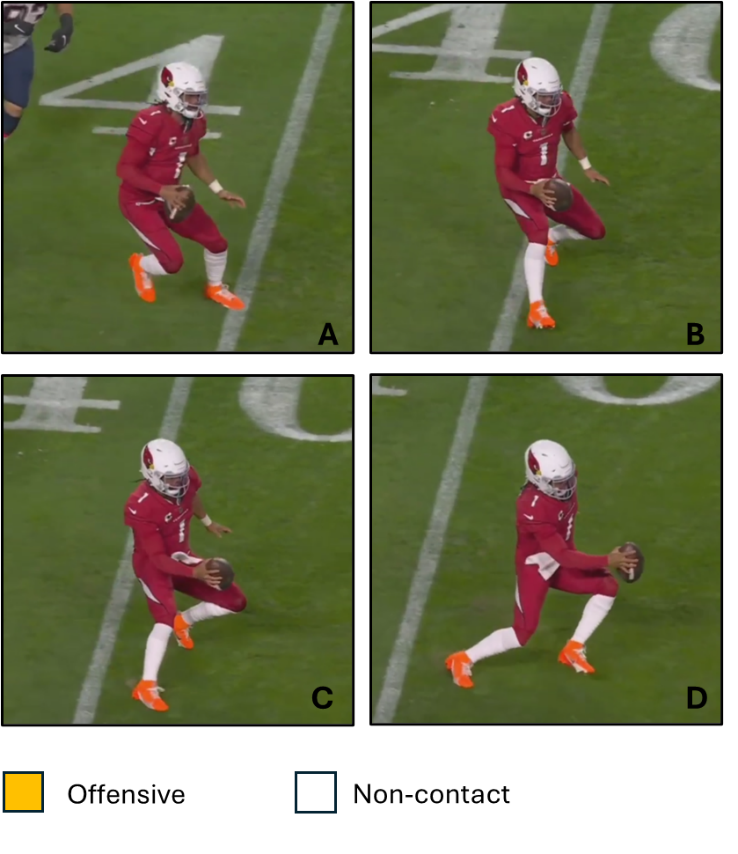
Offensive change of direction***

1. The ball carrier performs a cutting maneuver from the left leg to the right leg. No contact with the opponent.
2. Initial contact, right foot. Heel strike planting ahead of the athlete’s center of mass.
3. Injury frame, right knee ACL injury. The upper body travels towards the right side, right hip in flexion and abduction, foot planted in external rotation and eversion.
4. Loss of balance after injury.

***Deceleration from sprinting***

1.
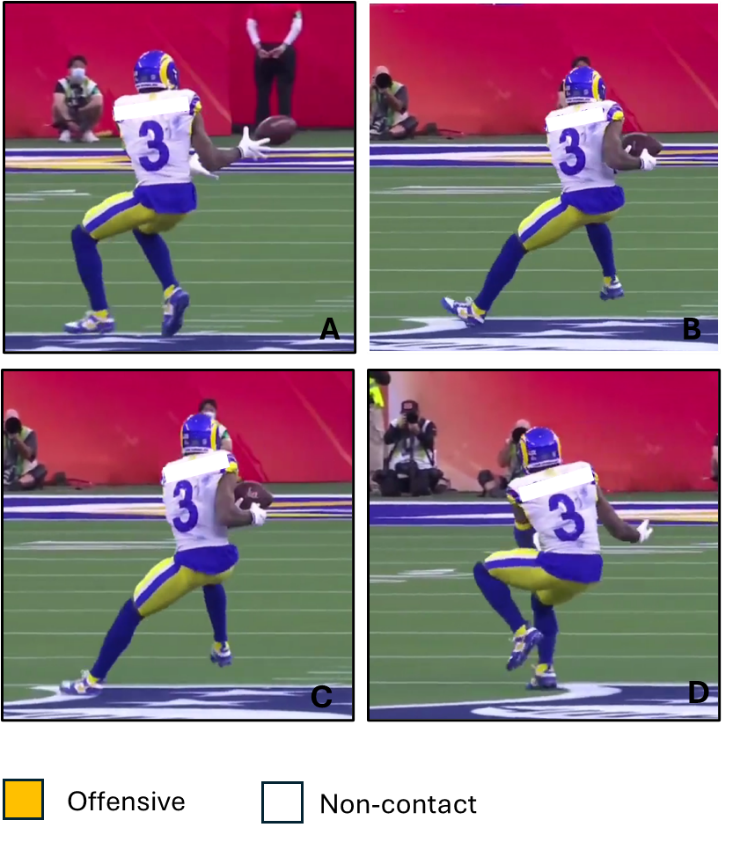
 The offensive player performs a rapid deceleration to catch the ball thrown behind the intended direction, and from the right side of his body. No contact with an opponent.
2. Initial contact left foot. Heel strike planting ahead of the athlete’s center of mass. Upper body rotated to the right.
3. Injury frame, left knee ACL injury. The player continues upper body rotation to the right with shallow knee flexion and foot planted to the ground.
4. Reaction and loss of balance after injury.

***Being blocked***

1. **
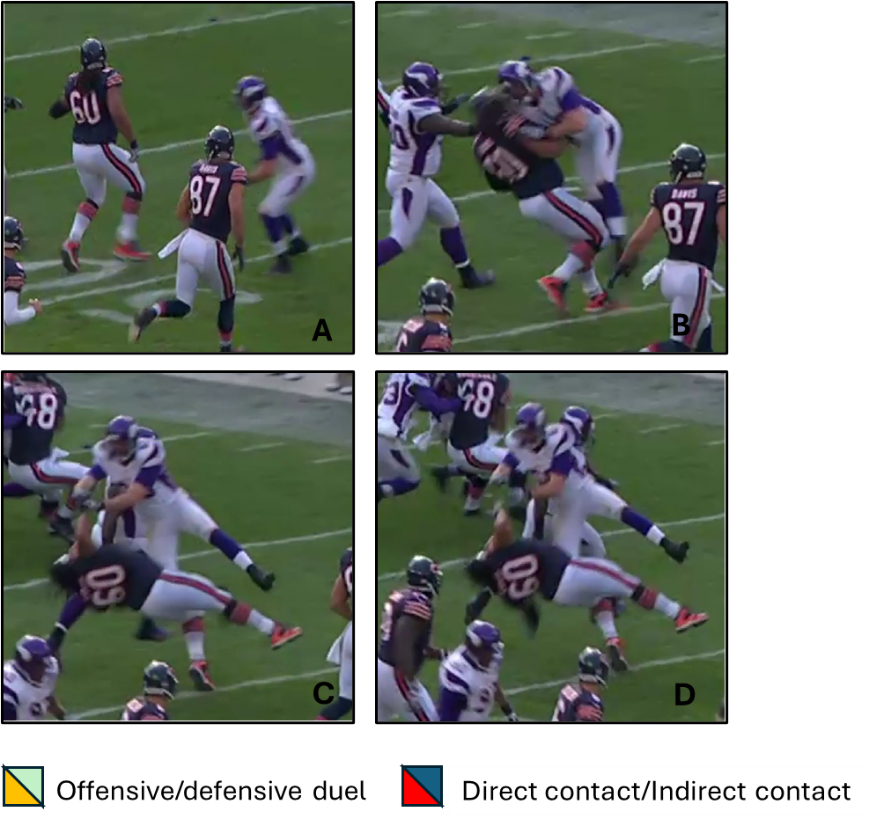
**The offensive player (navy blue jersey) approaching the defensive blocker with attention to the ball in play to his left.
2. Initial ground contact, left foot strikes the simultaneously as the upper body is hit by defensive blocker resulting in a back-weighted position on a planted foot.
3. Injury frame, left knee ACL injury. Foot planted in external rotation, upper body back weighted and tilted to the left while falling.

D. Forceful knee valgus, before the foot lets go off the ground and player falls.

All images created by the authors from publicly available video footage (Youtube.com)

**Rugby**

***Offensive change of direction***

1. **
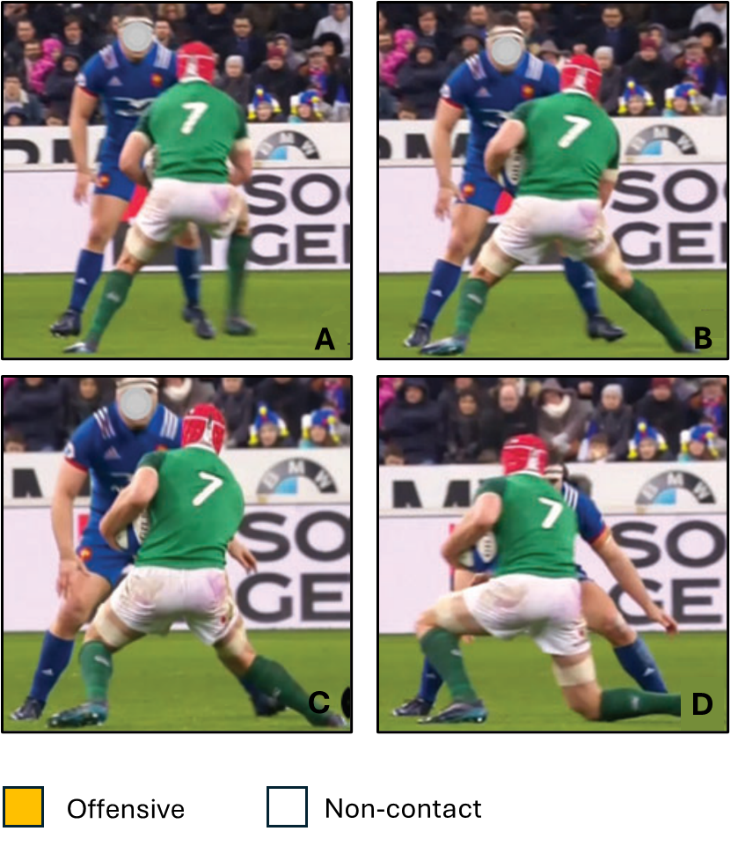
**The offensive player performs a sidestep cutting maneuver from the left to the right to deceive the opponent player.
2. Initial ground contact, right foot. Wide cut with a straight knee and upper body moment to the right.
3. Injury frame, right knee ACL injury. Hip abduction and shallow knee flexion with the right foot planted in external rotation and eversion.
4. Loss of balance after injury.

***
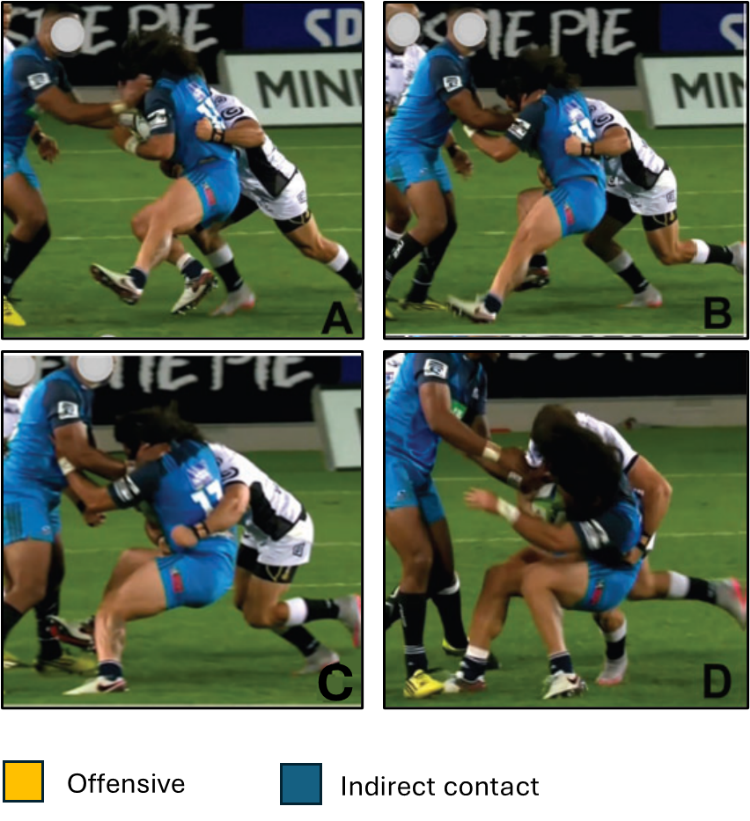
Being tackled***

1. Player contact to the upper body while in possession of the ball.
2. Initial ground contact, left leg. Heel strike ahead of center of mass, hip abduction and continued horizontal pressure to upper body from behind.
3. Injury frame, left knee ACL injury. Contralateral upper body rotation and foot planted in external rotation.
4. Loss of balance after injury.

*
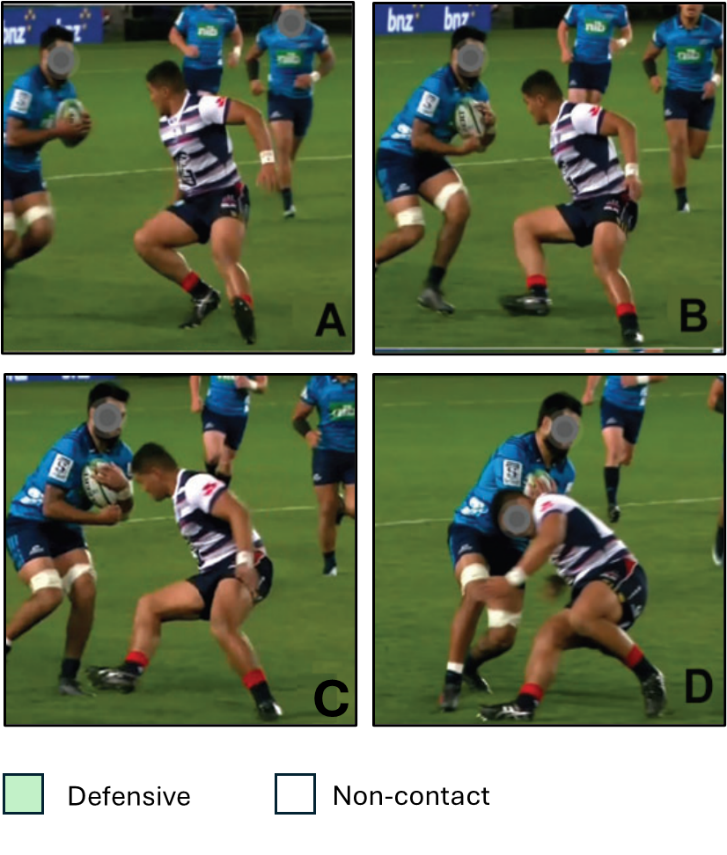
****Defensive pressing***

A. The defensive player tracks (jersey with stripes) the offensive player for a pressing action.

B. Initial ground contact, left leg. Heel-strike planting to decelerate and perform a change of direction movement to the right.

C. Injury frame, left knee ACL injury. Contralateral upper body rotation, hip abduction and foot planted in external rotation.

D. Loss of balance after injury.

All images are reproduced with permission from the publisher of the original article by Della Villa et al. [19].

**Australian rules football**

***Offensive change of direction***

**
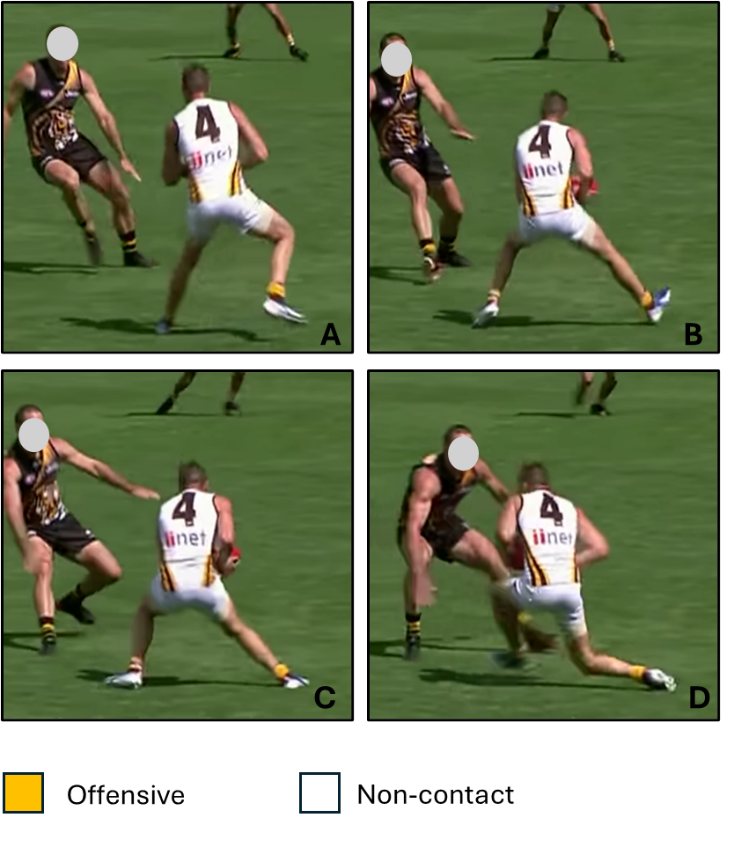
**

1. The ball carrier performs a wide cutting maneuver from the left leg to the right leg. No contact from opponent.
2. Initial contact, right foot. Heel strike and wide foot plant (large hip abduction angle).
3. Injury frame, right knee ACL injury. Upper body moment to the right side, right hip in abduction, straight knee and foot planted in external rotation and eversion.

D. Dynamic knee valgus and loss of balance.

***
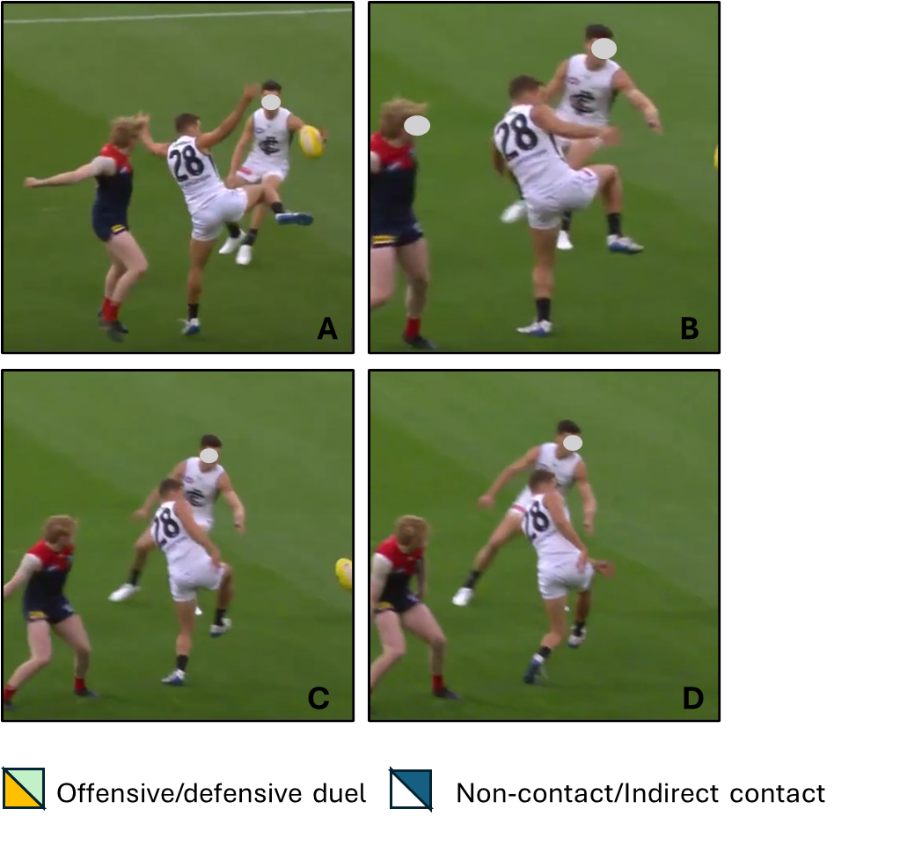
Landing***

1. Aerial duel with mechanical perturbation to arms/chest from the right side.
2. Initial contact left foot. Stiff single-leg landing on mid-foot in external rotation. Upper body in lateral flexion to the left and right leg swing in abduction to counterbalance.
3. Injury frame, left knee ACL injury. Axial compression with shallow knee flexion, foot in external rotation and eversion.
4. Dynamic knee valgus and loss of balance after injury.

***Defensive pressing***

**
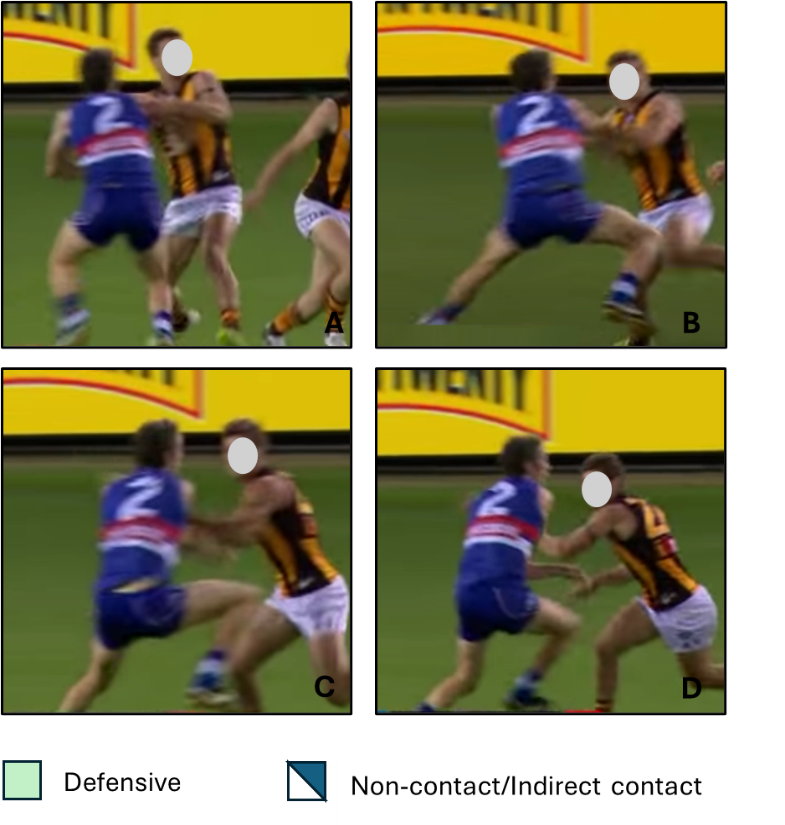
**

1. The defensive player approaches the ball receiver at medium speed. Upper body contact to opponent.
2. Initial contact, left foot. The player decelerates and performs a sidestep cut in an effort to reach the ball or to tackle the opponent. Wide foot plant with ipsilateral trunk lean to maintain balance, rotates the trunk towards his right side and puts the entire load on his left leg.
3. Injury frame, left knee ACL injury. Left hip in abduction and the ankle in eversion.
4. Reaction and loss of balance after injury.

All images created by the authors from publicly available video footage (Youtube.com)

**Volleyball**

***
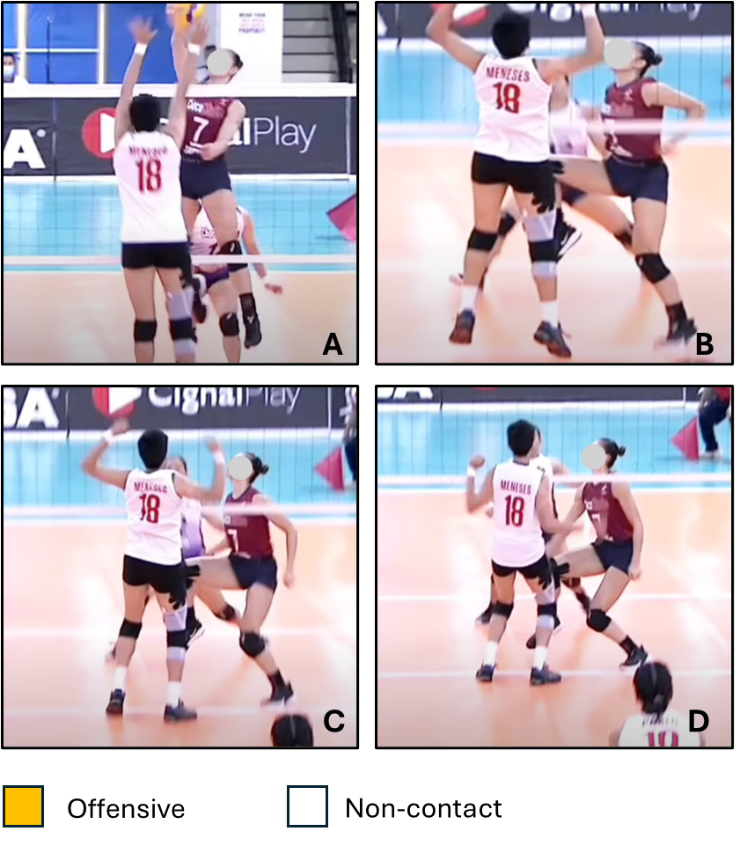
Spiking***

1. The offensive player (red jersey) performs a jump attack close to the net with visual distraction from a defensive blocker (white jersey).
2. Initial contact. Stick landing (feet parallel), left foot touches the ground just before the right foot with high vertical ground reaction force. Hip abducted and contralateral trunk rotation.
3. Injury frame, left knee ACL injury. High axial compression force, shallow knee flexion and valgus.
4. Dynamic knee valgus.

All images created by the authors from publicly available video footage (Youtube.com)

**Netball**

***
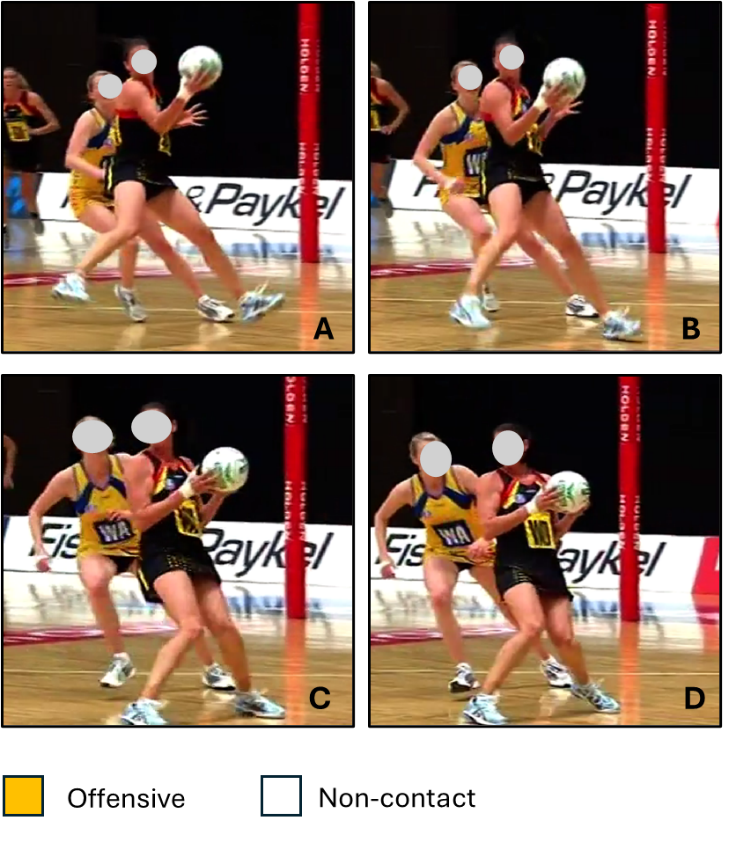
Jump stop***

1. Offensive player performs a bilateral jump stop task when receiving the ball from the left.
2. Initial ground contact. Wide stance-position, upright upper body rotated to the right, heel-strike landing with the left leg just before the right leg landing.
3. Injury frame, left knee ACL injury. Knee valgus and internal rotation motion.
4. Dynamic knee valgus.

All images created by the authors from publicly available video footage (Youtube.com)

**Badminton**

***Single leg landing***

1. ***
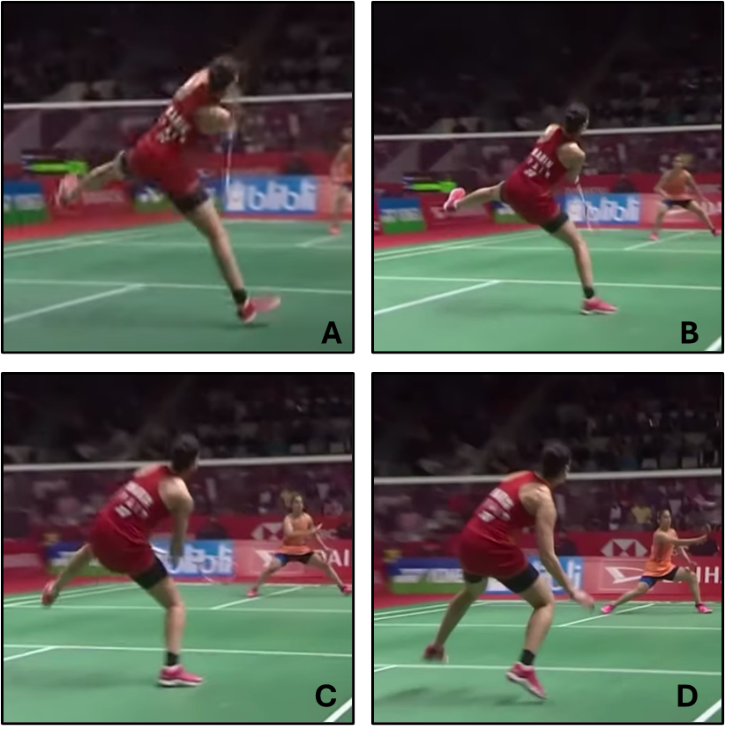
***A left-handed player moves to the right side of the rear court to perform an overhead stroke. Right leg heel-strike landing with ipsilateral trunk flexion at initial ground contact.
2. Stiff landing, shallow knee flexion, and hip abduction, left leg swing to counterbalance.
3. Injury frame, right knee ACL injury. Hip internal rotation and knee valgus.
4. Loss of balance after injury.

***Change of direction***

**
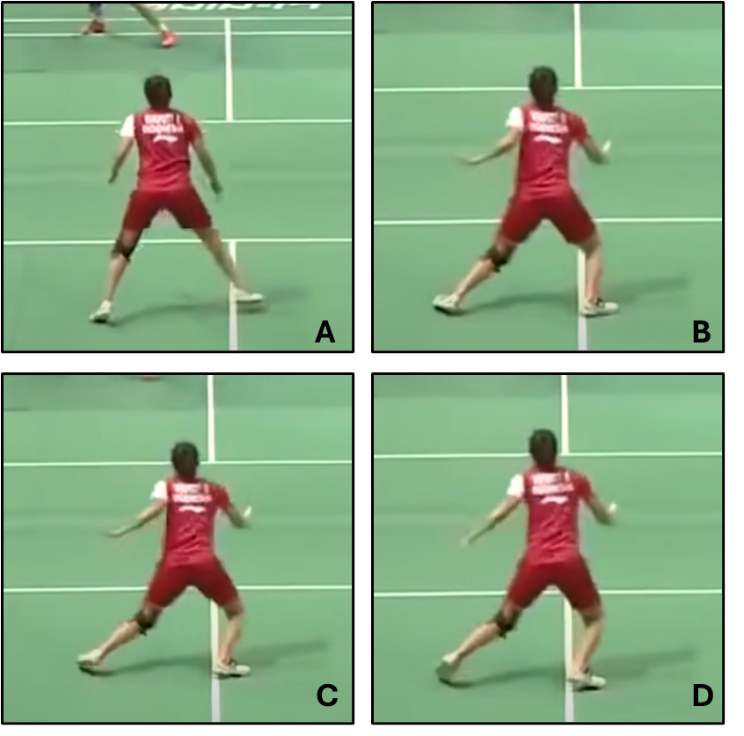
**A. Right-handed player prepares for a return, stepping from the right to the left foot for change of direction to the right.

B. Initial ground contact with the left foot. Hip in abduction, externally rotated foot and subsequent knee valgus.

C. Injury frame, left knee ACL injury. Shallow knee flexion and dynamic valgus.

D. Loss of balance after injury.

All images created by the authors from publicly available video footage (Youtube.com)

**Alpine skiing**


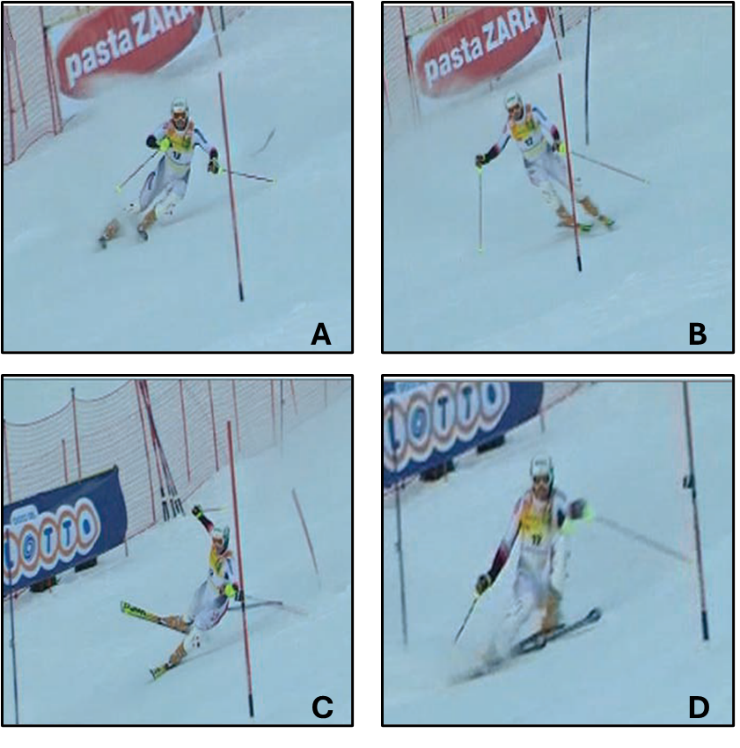
***Slip and catch***

1. The skier falls behind the line due to a sudden change in the course's rhythm.
2. Subsequently, the skier enters a hairpin turn too directly in a backward-weighted position.
3. In an attempt to navigate the next gate, at the inner ski turn he loses snow contact with the outer (right) ski.
4. Injury frame, right knee ACL injury. The skier trying to recover from the unbalanced position, the outer ski unexpectedly catches the inside edge, resulting in a forceful inward twist of the right knee into valgus and internal rotation.

***Landing back weighted***

1.
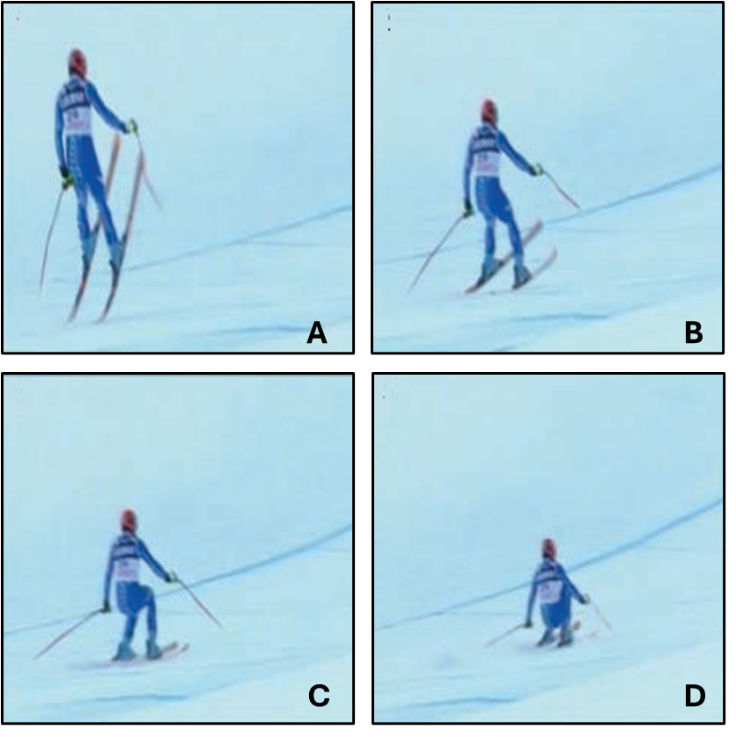
The skier is out of balance backward and lands from a jump on the ski tails with a large clap angle.
2. The right ski hits the snow surface slightly before the left ski.
3. Injury frame, left knee ACL injury. The skier tries to recover from the back weighted landing position, which forces the quadriceps to contract aggressively and likely induces high anterior shear forces on the ACL.
4. The skier falls backward to the left.

***Dynamic snowplow***


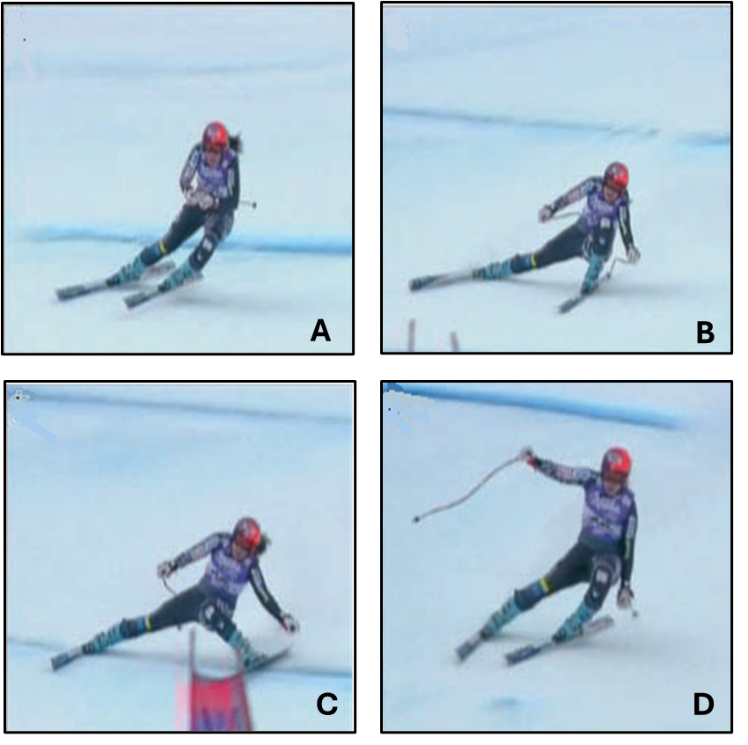
A. The skier loses ground contact with the inner (left) ski and gets out of balance backward and inward.

1. The right ski drifts away from the body’s center of mass, while the inner ski rolls from the outside edge to the inside edge.

C. Injury frame, left knee ACL injury. The inside edge on the inner ski engages the snow surface, forcing the left knee into valgus and internal rotation.

D. The skier regains balance from a snowplow position and eventually makes a complete stop.

All images reproduced with permission from the publisher of the original article by Bere et al. [73]

**Javelin throwing**

**
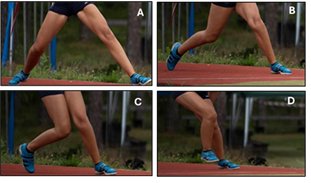
** A. At planting for delivery, the thrower lands on a stiff knee (shallow flexion angle and low angular velocity of knee flexion).

B. Injury frame, left knee ACL injury. Anterior shear force upon stiff landing with vertical ground reaction forces

C. Subsequent increase in knee valgus and internal rotation.

D. Reaction and unloading after injury.

Images created by the authors from original video material.
